# Supplementary material for: A probabilistic modeling framework for genomic networks incorporating sample heterogeneity
Source: Cell Rep Methods. 2025 Feb 14;5(2):100984. doi: 10.1016/j.crmeth.2025.100984 (PMC11955270; doi:10.1016/j.crmeth.2025.100984)
Supplement: Document S1. Figures S1–S5, Table S1, Algorithm S1, Methods S1 and S2 [file mmc1.pdf]

Cell Reports Methods, Volume *5*

Supplemental information

A probabilistic modeling framework for genomic networks incorporating sample heterogeneity

Liying Chen, Satwik Acharyya, Chunyu Luo, Yang Ni, and Veerabhadran Baladandayuthapani

GraphR: a probabilistic modeling framework for genomic networks incorporating sample heterogeneity

Liying Chen[1,*], Satwik Acharyya[2,*], Chunyu Luo[3],
Yang Ni[4], Veerabhadran Baladandayuthapani[1,†]

[1]Department of Biostatistics, University of Michigan, Ann Arbor, MI

[2]Department of Biostatistics, University of Alabama at Birmingham, Birmingham, AL

[3]Division of Biostatistics, University of Pennsylvania, Philadelphia, PA

[4]Department of Statistics, Texas A&M University, College Station, TX

January 13, 2025

[*]These authors contributed equally.

[†]Corresponding author: veerab@umich.edu.

Methods S1: Derivation of GraphR, related to Figure 1

The likelihood is expressed as

$$
\begin{aligned}
p(\boldsymbol{\theta}, \boldsymbol{Y}, \boldsymbol{X}) \propto \prod_{i=1}^{p} &\left\{ \left| \frac{1}{\omega_{ii}} I_n \right|^{-\frac{1}{2}} \exp \left[-\frac{\omega_{ii}}{2} \left\| Y_i - \sum_{j \neq i} \gamma_{ij}(X) \odot Y_j \right\|^2 \right] \right\} \times \\
&\prod_{i=1}^{p} \prod_{j \neq i}^{p} \prod_{l=1}^{q} \left\{ \left(\frac{1}{\tau_{il}} \right)^{-\frac{1}{2}} \exp \left[-\frac{\tau_{il}}{2} (b_{ijl})^2 \right] (\pi_{ijl})^{s_{ijl}} (1 - \pi_{ijl})^{1 - s_{ijl}} \right\} \times \\
&\prod_{i=1}^{p} \prod_{l=1}^{q} \left\{ (\tau_{il})^{a_\tau - 1} \exp \left[-b_\tau \tau_{il} \right] \right\} \times \\
&\prod_{i=1}^{p} \prod_{j \neq i}^{p} \prod_{l=1}^{q} \left\{ (\pi_{ijl})^{a_\pi - 1} (1 - \pi_{ijl})^{b_\pi - 1} \right\}
\end{aligned}
\tag{A.5}
$$

$$
\begin{aligned}
\log p(\boldsymbol{\theta}, \boldsymbol{Y}, \boldsymbol{X}) = &\text{Const} + \sum_{i=1}^{p} \left\{ \frac{n}{2} \log(\omega_{ii}) - \frac{\omega_{ii}}{2} \left\| Y_i + \frac{1}{\omega_{ii}} \sum_{j \neq i}^{p} \sum_{l=1}^{q} b_{ijl} s_{ijl} X_l \odot Y_j \right\|^2 \right\} \\
&+ \sum_{i=1}^{p} \sum_{l=1}^{q} \left\{ \left(\frac{p-1}{2} + a_\tau - 1 \right) \log(\tau_{il}) - \left(b_\tau + \frac{1}{2} \sum_{j \neq i}^{p} (b_{ijl})^2 \right) \tau_{il} \right\} \\
&+ \sum_{i=1}^{p} \sum_{j \neq i}^{p} \sum_{l=1}^{q} \left\{ (s_{ijl} + a_\pi - 1) \log(\pi_{ijl}) + (b_\pi - s_{ijl}) \log(1 - \pi_{ijl}) \right\}.
\end{aligned}
\tag{A.6}
$$

Due to the dependence between $\boldsymbol{b}$ and $\boldsymbol{s}$[14], the mean-field assumption is considered as:

$$q_{\text{vb}}(\boldsymbol{b}, \boldsymbol{s}, \boldsymbol{\omega}, \boldsymbol{\pi}, \boldsymbol{\tau}) = q_{\text{vb}}(\boldsymbol{b}, \boldsymbol{s}) q_{\text{vb}}(\boldsymbol{\omega}) q_{\text{vb}}(\boldsymbol{\pi}) q_{\text{vb}}(\boldsymbol{\tau}).$$

We can obtain the update equation for each parameter as:

$$q_{\text{vb}}^{k}(\boldsymbol{\theta_k}) \propto \exp \left[\mathbb{E}_{-k}(\log p(\boldsymbol{\theta}, \boldsymbol{Y}, \boldsymbol{X})) \right].$$

a. Update of τ_{il}:

$$
\begin{aligned}
\log q_{\text{vb}}(\tau_{il}) &= \mathbb{E}_{-\tau_{il}}(l) \\
&= C + \left[\frac{p-1}{2} + a_\tau - 1 \right] \log \tau_{il} + \left[b_\tau + \frac{1}{2} \sum_{j \neq i}^{p} \mathbb{E}_{-\tau_{il}} (b_{ijl})^2 \right] \tau_{il}.
\end{aligned}
\tag{A.7}
$$

$$q_{\text{vb}}(\tau_{il}) \sim \Gamma \left(a_\tau + \frac{p-1}{2}, b_\tau + \frac{1}{2} \sum_{j \neq i}^{p} \mathbb{E}_{-\tau_{il}} (b_{ijl})^2 \right)$$

b. Update of π_{ijl}:

$$\begin{aligned}\log q_{\text{vb}}(\pi_{ijl}) &= \mathbb{E}_{-\pi_{ijl}}(l)\\ &= C + \left[\mathbb{E}_{-\pi_{ijl}}(s_{ijl}) + a_\pi - 1\right]\log(\pi_{ijl}) + \left[b_\pi - \mathbb{E}_{-\pi_{ijl}}(s_{ijl})\right]\log(1-\pi_{ijl}).\end{aligned} \tag{A.8}$$

$$q_{\text{vb}}(\pi_{ijl}) \sim \text{Beta}\left(\mathbb{E}_{-\pi_{ijl}}(s_{ijl}) + a_\pi, b_\pi - \mathbb{E}_{-\pi_{ijl}}(s_{ijl}) + 1\right)$$

c. Update of ω_{ii}:

$$\begin{aligned}\log q_{\text{vb}}(\omega_{ii}) &= \mathbb{E}_{-\omega_{ii}}(l)\\ &= C + \frac{n}{2}\log(\omega_{ii}) - \frac{\|Y_i\|^2}{2}\omega_{ii} - \frac{\mathbb{E}_{-\omega_{ii}}\|\sum_{j\neq i}^{p}\sum_{l=1}^{q} b_{ijl}s_{ijl}X_l\odot Y_j\|^2}{2}\left(\frac{1}{\omega_{ii}}\right).\end{aligned} \tag{A.9}$$

$$q_{\text{vb}}(\omega_{ii}) \sim \text{GIG}\left(\frac{n+2}{2}, \|Y_i\|^2, \mathbb{E}_{-\omega_{ii}}\|\sum_{j\neq i}^{p}\sum_{l=1}^{q} b_{ijl}s_{ijl}X_l\odot Y_j\|^2\right)$$

Here **GIG** represents generalized inverse Gaussian distribution.

d. Update of $\beta_{ijl} = b_{ijl}s_{ijl}$:
We Denote $M_{-(m,n)}^{-k} = \sum_{j\neq m}^{p}\sum_{l=1}^{q} b_{mjl}s_{mjl}X_l\odot Y_j - b_{mnk}s_{mnk}X_k\odot Y_n$ and

$$\begin{aligned}\log q_{\text{vb}}(b_{ijl}|s_{ijl}) &= \mathbb{E}_{-b_{ijl}|s_{ijl}}(l)\\ &= C - \frac{1}{2}\left[\mathbb{E}_{-b_{ijl}|s_{ijl}}(\tau_{il}) + \mathbb{E}_{-b_{ijl}|s_{ijl}}\left(\frac{1}{\omega_{ii}}\right)s_{ijl}\|X_l\odot Y_j\|^2\right](b_{ijl})^2\\ &\quad - \left[Y_i + \mathbb{E}_{-b_{ijl}|s_{ijl}}\left(\frac{1}{\omega_{ii}}\right)\mathbb{E}_{-b_{ijl}|s_{ijl}}M_{-(i,j)}^{-l}\right]^T [X_l\odot Y_j]\, s_{ijl}b_{ijl}.\end{aligned} \tag{A.10}$$

$$q_{\text{vb}}(b_{ijl}|s_{ijl}) \sim \mathbb{N}\left(\mu(s_{ijl}), \sigma^2(s_{ijl})\right)$$

$$\sigma^2(s_{ijl}) = \left[\mathbb{E}_{-b_{ijl}|s_{ijl}}(\frac{1}{\omega_{ii}})\|X_l\odot Y_j\|^2 s_{ijl} + \mathbb{E}_{-b_{ijl}|s_{ijl}}(\tau_{il})\right]^{-1}$$

$$\mu(s_{ijl}) = -\sigma^2(s_{ijl})\left\{\left[Y_i + \mathbb{E}_{-b_{ijl}|s_{ijl}}\left(\frac{1}{\omega_{ii}}M_{-(i,j)}^{-l}\right)\right]^T [X_l\odot Y_j]s_{ijl}\right\}$$

The mariginal density of $q_{\text{vb}}(s_{ijl})$ is obtained by integrating the joint density of $q_{\text{vb}}(b_{ijl}, s_{ijl})$

as

$$
\begin{aligned}
q_{\mathrm{vb}}(s_{ijl}) &= \int \exp\left\{\log\, q_{\mathrm{vb}}(b_{ijl}, s_{ijl})\right\} db_{ijl} \\
&= \exp\left\{s_{ijl}\ \mathbb{E}_{-s_{ijl}}\mathrm{logit}(\pi_{ijl})\right\}\ \int \mathbb{N}_{b_{ijl}}\left(\mu(s_{ijl}), \sigma^2(s_{ijl})\right)\sigma(s_{ijl})\mathrm{exp}\ \left(\frac{\mu^2(s_{ijl})}{2\sigma^2(s_{ijl})}\right) db_{ijl} \\
&= \sigma(s_{ijl})\mathrm{exp}\left\{s_{ijl}\ \mathbb{E}_{-s_{ijl}}\mathrm{logit}(\pi_{ijl}) + \left(\frac{\mu^2(s_{ijl})}{2\sigma^2(s_{ijl})}\right)\right\}.
\end{aligned}
$$

$$
\log[q_{\mathrm{vb}}(s_{ijl})] = C + \log(\sigma(s_{ijl})) + s_{ijl}\ \mathbb{E}_{-s_{ijl}}\mathrm{logit}(\pi_{ijl}) + \frac{\mu^2(s_{ijl})}{2\sigma^2(s_{ijl})}
$$

$$
\log[q_{\mathrm{vb}}(s_{ijl}=0)] = C - \frac{1}{2}\log\mathbb{E}_{-s_{ijl}}\tau_{ijl}
$$

$$
\begin{aligned}
\log[q_{\mathrm{vb}}(s_{ijl}=1)] &= C + \mathbb{E}_{-s_{ijl}}\mathrm{logit}(\pi_{ijl}) - \frac{1}{2}\log\left[\mathbb{E}_{-s_{ijl}}(\frac{1}{\omega_{ii}})\|X_l \odot Y_j\|^2 + \mathbb{E}_{-s_{ijl}}(\tau_{il})\right] \\
&+ \frac{1}{2}\left[\mathbb{E}_{-s_{ijl}}(\frac{1}{\omega_{ii}})\|X_l \odot Y_j\|^2 + \mathbb{E}_{-s_{ijl}}(\tau_{il})\right]^{-1}\left[(X_l \odot Y_j)^T(Y_i + \mathbb{E}_{-s_{ijl}}\left(\frac{1}{\omega_{ii}}\right)\mathbb{E}_{-s_{ijl}}M^{-l}_{-(i,j)})\right]^2
\end{aligned}
$$

$$
\begin{aligned}
& s_{ijl} \sim \mathrm{Ber}(\psi_{ijl}) \\
& \log\, q_{\mathrm{vb}}(s_{ijl}) = C + s_{ijl}\mathrm{logit}(\psi_{ijl}) \\
& \psi_{ijl} = \mathbb{E}_{-s_{ijl}}\mathrm{logit}(\pi_{ijl}) - \frac{1}{2}log\left[\mathbb{E}_{-s_{ijl}}(\frac{1}{\omega_{ii}})\|X_l \odot Y_j\|^2 + \mathbb{E}_{-s_{ijl}}(\tau_{il})\right] + \frac{1}{2}log\mathbb{E}_{-s_{ijl}}\tau_{il} \\
& + \frac{1}{2}\left[\mathbb{E}_{-s_{ijl}}(\frac{1}{\omega_{ii}})\|X_l \odot Y_j\|^2 + \mathbb{E}_{-s_{ijl}}(\tau_{il})\right]^{-1}\left[(X_l \odot Y_j)^T(Y_i + \mathbb{E}_{-s_{ijl}}\left[\frac{1}{\omega_{ii}}\right]\mathbb{E}_{-s_{ijl}}M^{-l}_{-(i,j)})\right]^2
\end{aligned}
$$

The evidence lower bound is defined as:

$$
\begin{aligned}
L[q_{\mathrm{vb}}(\boldsymbol{\theta})] &= \int q_{\mathrm{vb}}(\boldsymbol{\theta})\log\left(p(\boldsymbol{\theta}, \boldsymbol{Y}, \boldsymbol{X})/q_{\mathrm{vb}}(\boldsymbol{\theta})\right) d\boldsymbol{\theta} \\
&= \mathbb{E}_{q_{\mathrm{vb}}(\boldsymbol{\theta})}\ \log(p(\boldsymbol{\theta}, \boldsymbol{Y}, \boldsymbol{X})) - \mathbb{E}_{q_{\mathrm{vb}}(\boldsymbol{\theta})}[q_{\mathrm{vb}}(\boldsymbol{\theta})] \\
&= \mathbb{E}_{q_{\mathrm{vb}}(\boldsymbol{\theta})}\ \log(p(\boldsymbol{\theta}, \boldsymbol{Y}, \boldsymbol{X})) - \sum_{i=1}^{p}\mathbb{E}_{q_{\mathrm{vb}}(\boldsymbol{\theta})}[q_{\mathrm{vb}}(\omega_{ii})] - \sum_{i=1}^{p}\sum_{l=1}^{q}\mathbb{E}_{q_{\mathrm{vb}}(\boldsymbol{\theta})}[q_{\mathrm{vb}}(\tau_{il})] \\
&- \sum_{i=1}^{p}\sum_{j\neq i}^{p}\sum_{l=1}^{q}\left\{\mathbb{E}_{q_{\mathrm{vb}}(\boldsymbol{\theta})}[q_{\mathrm{vb}}(b_{ijl}, s_{ijl})] + \mathbb{E}_{q_{\mathrm{vb}}(\boldsymbol{\theta})}[q_{\mathrm{vb}}(\pi_{ijl})]\right\}.
\end{aligned}
$$

Notably, $-\mathbb{E}_{q_{\mathrm{vb}}(\boldsymbol{\theta})}[q_{\mathrm{vb}}(\omega_{ii})]$, $-\mathbb{E}_{q_{\mathrm{vb}}(\boldsymbol{\theta})}[q_{\mathrm{vb}}(\tau_{il})]$, $-\mathbb{E}_{q_{\mathrm{vb}}(\boldsymbol{\theta})}[q_{\mathrm{vb}}(b_{ijl}, s_{ijl})]$, $-\mathbb{E}_{q_{\mathrm{vb}}(\boldsymbol{\theta})}[q_{\mathrm{vb}}(\pi_{ijl})]$ are entropies of GIG, Gamma, Normal, Bernoulli and Beta distributions, which have a close form. We denote entropy as $H(\cdot)$ and all the expectations below are taken w.r.t $q_{\mathrm{vb}}(\boldsymbol{\theta})$.

a. Derivation of$\mathbb{E}\ \log(p(\boldsymbol{\theta}, \boldsymbol{Y}, \boldsymbol{X}))$:

$$
\begin{aligned}
\mathbb{E}\ \log(p(\boldsymbol{\theta}, \boldsymbol{Y}, \boldsymbol{X})) = & \sum_{i=1}^{p} \Bigg\{ -\frac{n+(p-1)q}{2}\log 2\pi + q\left[a_\tau \log b_\tau - \log\Gamma(a_\tau)\right] \\
& + (p-1)q\left[\log\Gamma(a_\pi + b_\pi) - \log\Gamma(a_\pi b_\pi)\right] \\
& + \frac{n}{2}\mathbb{E}(\log\omega_{ii}) - \frac{\|Y_i\|^2}{2}\mathbb{E}\omega_{ii} - \mathbb{E}(\omega_{ii}^{-1})\mathbb{E}\left\|\sum_{j\neq i}^{p}\sum_{l=1}^{q}\beta_{ijl}Z_l \odot Y_j\right\|^2 \\
& - Y_i^T\left(\sum_{j\neq i}^{p}\sum_{l=1}^{q}\mathbb{E}\beta_{ijl}Z_l \odot Y_j\right) \\
& + \sum_{l=1}^{q}\left[\left(\frac{p-1}{2} + a_\tau - 1\right)\mathbb{E}\left(\log\tau_{il}\right) - \left(b_\tau + \frac{\sum_{j\neq i}^{p}\mathbb{E}b_{ijl}^2}{2}\right)\mathbb{E}\tau_{il}\right] \\
& + \sum_{j\neq i}^{p}\sum_{l=1}^{q}\left[\left(\mathbb{E}s_{ijl} + a_\pi - 1\right)\mathbb{E}(\log\pi_{ijl}) + (b_\pi - \mathbb{E}s_{ijl})\right]\mathbb{E}\left(\log(1-\pi_{ijl})\right)\Bigg\}
\end{aligned}
$$

b. Derivation of $H(\tau_{il})$

$$
\begin{aligned}
H(\tau_{il}) = & -\left[a_\tau + \frac{p-1}{2}\right]\log\left[b_\tau + \frac{1}{2}\sum_{j\neq i}^{p}\mathbb{E}b_{ijl}^2\right] + \log\,\Gamma(a_\tau + \frac{p-1}{2}) \\
& - \left[a_\tau + \frac{p-1}{2} - 1\right]\mathbb{E}(\log\,\tau_{il}) + \left[b_\tau + \frac{1}{2}\sum_{j\neq i}^{p}\mathbb{E}b_{ijl}^2\right]\mathbb{E}\tau_{il}
\end{aligned}
$$

c. Derivation of $H(\pi_{ijl})$

$$
\begin{aligned}
H(\pi_{ijl}) = & -\log\,\Gamma(a_\pi + b_\pi + 1) + \log\,\Gamma(\mathbb{E}s_{ijl} + a_\pi) + \log\,\Gamma(b_\pi + 1 - \mathbb{E}s_{ijl}) \\
& - (\mathbb{E}s_{ijl} + a_\pi - 1)\mathbb{E}\log\,\pi_{ijl} - (b_\pi - \mathbb{E}s_{ijl})\mathbb{E}\log\,(1-\pi_{ijl})
\end{aligned}
$$

d. Derivation of $H(\omega_{ii})$

Denote $a_{\omega_{ii}} = \|Y_i\|^2$ and $b_{\omega_{ii}} = \mathbb{E}\|\sum_{j\neq i}^{p}\sum_{l=1}^{q}\beta_{ijl}Z_l \odot Y_j\|^2$

$$
H(\omega_{ii}) = -\frac{n+2}{4}\log\frac{a_{\omega_{ii}}}{b_{\omega_{ii}}} + \log(2K_{(n+2)/2}\sqrt{a_{\omega_{ii}}b_{\omega_{ii}}}) - \frac{n}{2}\mathbb{E}(\log\omega_{ii}) + \frac{a_{\omega_{ii}}}{2}\mathbb{E}\omega_{ii} + \frac{b_{\omega_{ii}}}{2}\mathbb{E}\omega_{ii}^{-1}
$$

e. Derivation of $H(b_{ijl}, s_{ijl})$

$$
H(b_{ijl}, s_{ijl}) = H(b_{ijl}|s_{ijl}) + H(s_{ijl})
$$

where

$$H(b_{ijl}|s_{ijl}) = \frac{1}{2}\log\left[2\pi\sigma^2(s_{ijl})\right] + \frac{1}{2}$$

$$H(s_{ijl}) = -\psi_{ijl}\,\log(\psi_{ijl}) - (1-\psi_{ijl})\,\log(1-\psi_{ijl})$$

Combining all the previous entropy derivations, we have the following expression of evidence lower bound (ELBO) as

$$\begin{aligned}
L[q_{\text{vb}}(\boldsymbol{\theta})] =& -\frac{np + p(p-1)q}{2}\log 2\pi \\
&+ pq\left[a_\tau \log b_\tau - \log\Gamma(a_\tau) + \log\Gamma(a_\tau + \frac{p-1}{2})\right] \\
&+ p(p-1)q\left[\log\Gamma(a_\pi + b_\pi) - \log\Gamma(a_\pi b_\pi) - \log\Gamma(a_\pi + b_\pi + 1)\right] \\
&- \sum_{i=1}^{p}\left\{Y_i^T\left(\sum_{j\neq i}^{p}\sum_{l=1}^{q}\mathbb{E}\beta_{ijl}Z_l \odot Y_j\right) - \frac{n+2}{4}\log\frac{a_{\omega_{ii}}}{b_{\omega_{ii}}} + \log(2K_{(n+2)/2}\sqrt{a_{\omega_{ii}}b_{\omega_{ii}}})\right\} \\
&- \left[a_\tau + \frac{p-1}{2}\right]\sum_{i=1}^{p}\sum_{l=1}^{q}\left\{\log\left[b_\tau + \frac{1}{2}\sum_{j\neq i}^{p}\mathbb{E}b_{ijl}^2\right]\right\} \\
&+ \sum_{i=1}^{p}\sum_{j\neq i}^{p}\sum_{l=1}^{q}\Bigg\{\log\,\Gamma(\mathbb{E}s_{ijl} + a_\pi) + \log\,\Gamma(b_\pi + 1 - \mathbb{E}s_{ijl}) \\
&+ \frac{1}{2}\log\left[2\pi\sigma^2(s_{ijl})\right] + \frac{1}{2} - \psi_{ijl}\,\log(\psi_{ijl}) - (1-\psi_{ijl})\,\log(1-\psi_{ijl})\Bigg\}.
\end{aligned}$$

Methods S2: Implementation details of GraphR, FGL, GGL, LASICH, jointGHS, Bayesian Edge Regression, BGGM, GLASSO, k-GLASSO, related to Figure 3.

- **FGL and GGL**: Tuning parameters λ_1 and λ_2 are selected from a 20×20 grid, evenly spaced between 0.05 to 0.5 for λ_1 and 0.001 to 0.01 for λ_2, using the approximated Akaike Information Criterion (AIC). The ROC curve is generated by varying the tuning parameters λ_1 and λ_2.

- **LASICH**: Tuning parameters are selected from a 50×50 grid, evenly spaced between 1 to 50 for both λ_1 and λ_2, using the Bayesian Information Criterion (BIC). The ROC curve is generated by varying the tuning parameters λ_1 and λ_2.

- **jointGHS**: The algorithm is implemented using the `jointGHS` package, with 'epsilon' = 0.001 and 'AIC_eps' = 0.001. The ROC curve is generated by varying the tuning parameter τ^2, with 100 evenly spaced values ranging from 0.001 to 20.

- **Bayesian Edge Regression**: MCMC is run for 20,000 iterations after a burn-in of 10,000, with thinning applied to retain every 10th sample.

- **BGGM**: The model is run for 10,000 iterations after a burn-in of 5,000. The ROC curve is obtained based on different thresholds of posterior probabilities for edge inclusion.

- **GLASSO**: The tuning parameter λ is selected using the stability approach[6]. The ROC curve is generated by varying the tuning parameter λ.

- **k-GLASSO**: Tuning parameters λ_i and h_i for each subject are selected using AIC, based on a 20×20 grid evenly spaced between 0.1 to 1. The ROC curve is generated based on varying the tuning parameters λ_i and h_i for each subject.

- **GraphR**: We are interested in the parameter selection on both intrinsic factors and edges level. Point estimators of each model parameters are obtained by using expectation with respect to the approximation of posterior distribution, for example the point estimates $\hat{\pi}_{ijl} = \int \pi_{ijl}\ \hat{q}(\pi_{ijl})d\pi_{ijl} = E_{\hat{q}}(\pi_{ijl})$. With respect to selection of intrinsic factors, we use posterior inclusion probability (PIP) of intrinsic factors, which are defined as $\hat{\boldsymbol{p}}_{\boldsymbol{ij}} := [\hat{p}_{ij1}, ...\hat{p}_{ijq}]^T = [E_{\hat{q}}(s_{ij1}), ...E_{\hat{q}}(s_{ijq})]^T$. For the edge selection between Y_i and Y_j, we take both the magnitude of external coefficient $E_{\hat{q}}(\boldsymbol{b}_{ij})$ and PIP of intrinsic factors $E_{\hat{q}}(\boldsymbol{s}_{ij})$ into consideration, and thus define PIP of edges as $\phi_{ij} := \sum_{l=1}^{q}\left[\frac{\|E_{\hat{q}}(b_{ijl}s_{ijl})X_l\|^2}{\sum_{m=1}^{q}\left(\|E_{\hat{q}}(b_{ijm}s_{ijm})X_m\|^2\right)}\hat{p}_{ijl}\right]$. In the undirected graphical models, we have $\omega_{ij}(\mathbf{X}) = \omega_{ji}(\mathbf{X})$ due to the symmetric property of precision matrix, implying that s_{ijl} and s_{jil} should be 1 or 0 simultaneously. However we parallelize the regressions which leads to significant increase in time-efficacy and also breaks the dependence between s_{ijl} and s_{jil}. Thus $s_{ijl} = s_{jil}$ are not necessarily held during the estimation procedure. To ensure the symmetry of selection indicator, namely $\boldsymbol{s_{ij}} = \boldsymbol{s_{ji}}$, we use a

post-processing based approach following the steps from[7]. We denote κ_{ex} and κ_{edge} as the threshold of intrinsic factors and edge detection, where intrinsic factors will be selected or conditional dependence exists between edges if the corresponding PIP is larger than κ_{ex} and κ_{edge}. β_{ijl} is non-zero if and only if $\hat{p}_{ijl}^{min} := min(\hat{p}_{ijl}, \hat{p}_{jil}) > \kappa_{ex}$. Similarly, in the edge detection, $\phi_{ij}^{min} := min(\phi_{ij}, \phi_{ji}) > \kappa_{edge} \Leftrightarrow$ edge $\{i, j\}$ exists for $1 \leq i \neq j \leq p$. In terms of the selection of threshold on both external covariate level and edge level, we considered a Bayesian local FDR-based inference approach aiming to control the average Bayesian FDR at some level α[1,8]. Denote the threshold on external covariates or on edges at level α as $\kappa_{ex,\alpha}$ and $\kappa_{edge,\alpha}$ respectively, and denote $q_{ijl} = 1 - \hat{p}_{ijl}^{min}$ and $\tilde{q}_{ij} = 1 - \phi_{ij}^{min}$. Notably, $q_{ijl} = q_{jil}$ and $\tilde{q}_{ij} = \tilde{q}_{ji}$ due to the definition of $\hat{p}_{ijl}^{min}$ and ϕ_{ij}^{min}. PIPs of external covariates and edges represented that posterior probability that the corresponding external covariates or edges were presented from the model, then q_{ijl} and $\tilde{q}_{ij}$ can be regard as estimates of the local FDR for external covariates and edges, which are also known as Bayesian q-values[13]. This statement holds even if we have correlated data[3,1]. Based on q-values of external covariates, $\kappa_{ex,\alpha}$ is determined using follow procedure: (1) sort $\{q_{ijl}\}_{1\leq i<j\leq p, 1\leq l\leq q}$ in an ascending order and notated the ordered set as $\{q^{(t)}\}_{1\leq t\leq p(p-1)q/2}$ (2) Calculate the cumulative mean of $\{q^{(t)}\}$, and find the maximum t^* such that the cumulative mean of $q^{(t^*)}$ is smaller than α, a given significance level. (3) Set $\kappa_{ex,\alpha} = 1 - q^{(t^*)}$. Similar procedure were proposed when choosing $\kappa_{edge,\alpha}$ by replacing $\{q_{ijl}\}_{1\leq i<j\leq p, 1\leq l\leq q}$ with $\{\tilde{q}_{ij}\}_{1\leq i<j\leq p}$.

Algorithm 1 GraphR algorithm

Input: $\mathbf{Y}, \mathbf{X}$, tolerance

Output: Covariate dependent edges

while $\zeta >$ tolerance **do**

  for i in $1:p$ **do**

    for l in $1:q$ **do**

      Set $q_{\text{vb}}(\tau_{il}) \sim \Gamma\left(a_\tau + \frac{p-1}{2}, b_\tau + \frac{1}{2}\sum_{j\neq i}^{p} \mathbb{E}_{-\tau_{il}}\left(b_{ijl}\right)^2\right)$

    end for

    for j in $1:p$ and $j \neq i; l$ in $1:q$ **do**

      Set $q_{\text{vb}}(\pi_{ijl}) \sim \text{Beta}\left(\mathbb{E}_{-\pi_{ijl}}(s_{ijl}) + a_\pi, b_\pi - \mathbb{E}_{-\pi_{ijl}}(s_{ijl}) + 1\right)$

    end for

    Set $q_{\text{vb}}(\omega_{ii}) \sim \text{GIG}\left(\frac{n+2}{2}, \|Y_i\|^2, \mathbb{E}_{-\omega_{ii}}\|\sum_{j\neq i}^{p}\sum_{l=1}^{q} b_{ijl}s_{ijl}X_l \odot Y_j\|^2\right)$

    for j in $1:p$ and $j \neq i; l$ in $1:q$ **do**

      $\beta_{ijl} = b_{ijl}s_{ijl}$

      Set $q_{\text{vb}}(b_{ijl}|s_{ijl}) \sim \mathbb{N}\left(\mu(s_{ijl}), \sigma^2(s_{ijl})\right)$ where

      $\sigma^2(s_{ijl}) = \left[\mathbb{E}_{-b_{ijl}|s_{ijl}}(\frac{1}{\omega_{ii}})\|X_l \odot Y_j\|^2 s_{ijl} + \mathbb{E}_{-b_{ijl}|s_{ijl}}(\tau_{il})\right]^{-1}$

      $\mu(s_{ijl}) = -\sigma^2(s_{ijl})\left\{\left[Y_i + \mathbb{E}_{-b_{ijl}|s_{ijl}}\left(\frac{1}{\omega_{ii}}M_{-(i,j)}^{-l}\right)\right]^T [X_l \odot Y_j]s_{ijl}\right\}$

      Set $q_{vb}(s_{ijl}) \sim \text{Ber}(\psi_{ijl})$ where:

      $\psi_{ijl} = \mathbb{E}_{-s_{ijl}}\text{logit}(\pi_{ijl}) + \frac{1}{2}log\mathbb{E}_{-s_{ijl}}\tau_{il} -$
      $\frac{1}{2}log\left[\mathbb{E}_{-s_{ijl}}(\frac{1}{\omega_{ii}})\|X_l \odot Y_j\|^2 + \mathbb{E}_{-s_{ijl}}(\tau_{il})\right] +$
      $\frac{1}{2}\left[\mathbb{E}_{-s_{ijl}}(\frac{1}{\omega_{ii}})\|X_l \odot Y_j\|^2 + \mathbb{E}_{-s_{ijl}}(\tau_{il})\right]^{-1} \times$
      $\left[(X_l \odot Y_j)^T(Y_i + \mathbb{E}_{-s_{ijl}}\left[\frac{1}{\omega_{ii}}\right]\mathbb{E}_{-s_{ijl}}M_{-(i,j)}^{-l})\right]^2$

    end for

  end for

  ζ: maximum value of expectation difference for parameters before and after updates.

  Note: ζ can also be defined as improvement in evidence lower bound (ELBO), calculated as:

  $ELBO[q_{\text{vb}}(\boldsymbol{\theta})] = -\frac{np+p(p-1)q}{2}\text{log}2\pi + pq\left[a_\tau\text{log}b_\tau - \text{log}\Gamma(a_\tau) + \text{log}\Gamma(a_\tau + \frac{p-1}{2})\right] +$
  $p(p-1)q\left[\text{log}\Gamma(a_\pi + b_\pi) - \text{log}\Gamma(a_\pi b_\pi) - \text{log}\Gamma(a_\pi + b_\pi + 1)\right] -$
  $\sum_{i=1}^{p}\left\{Y_i^T\left(\sum_{j\neq i}^{p}\sum_{l=1}^{q}\mathbb{E}\beta_{ijl}Z_l \odot Y_j\right) - \frac{n+2}{4}\text{log}\frac{a_{\omega_{ii}}}{b_{\omega_{ii}}} + \text{log}(2K_{(n+2)/2}\sqrt{a_{\omega_{ii}}b_{\omega_{ii}}})\right\} -$
  $\left[a_\tau + \frac{p-1}{2}\right]\sum_{i=1}^{p}\sum_{l=1}^{q}\left\{\text{log}\left[b_\tau + \frac{1}{2}\sum_{j\neq i}^{p}\mathbb{E}b_{ijl}^2\right]\right\} +$
  $\sum_{i=1}^{p}\sum_{j\neq i}^{p}\sum_{l=1}^{q}\left\{\text{log }\Gamma(\mathbb{E}s_{ijl} + a_\pi) + \text{log }\Gamma(b_\pi + 1 - \mathbb{E}s_{ijl}) + \right.$
  $\left.\frac{1}{2}\text{log}\left[2\pi\sigma^2(s_{ijl})\right] + \frac{1}{2} - \psi_{ijl}\text{ log}(\psi_{ijl}) - (1-\psi_{ijl})\text{ log}(1-\psi_{ijl})\right\}$

end while

Algorithm S1. GraphR algorithm, related to Figure 1.

	Estimation strategy	Multiple-group graphs	Continuously-varying graphs	Extension to spatial covariates	Scalability	Uncertainty quantification	Positive definite
GraphR	Bayesian	✓	✓	✓	✓	✓	X
FGL, GGL [2]	Frequentist	✓	X	X	✓	X	✓
LASICH [12]	Frequentist	✓	X	X	✓	X	✓
Bayesian Joint Spike-and-Slab Graphical Lasso [4]	Bayesian	✓	X	X	✓	✓	✓
Gembag[16]	Bayesian	✓	X	X	✓	✓	✓
Joint graphical horseshoe [5]	Bayesian	✓	X	X	✓	X	✓
GGMReg[17]	Frequentist	✓	✓	X	✓	X	X
Multiple GGMs [11]	Bayesian	✓	X	X	X	✓	✓
Bayesian graphical regression [9]	Bayesian	✓	✓	X	X	X	Not required
Bayesian edge regression [15]	Bayesian	✓	✓	X	X	✓	X
GGMx[10]	Bayesian	✓	✓	X	X	X	✓

Table S1: Comparative overview of GraphR and other heterogeneous graphical modeling methods, related to Figure 3.

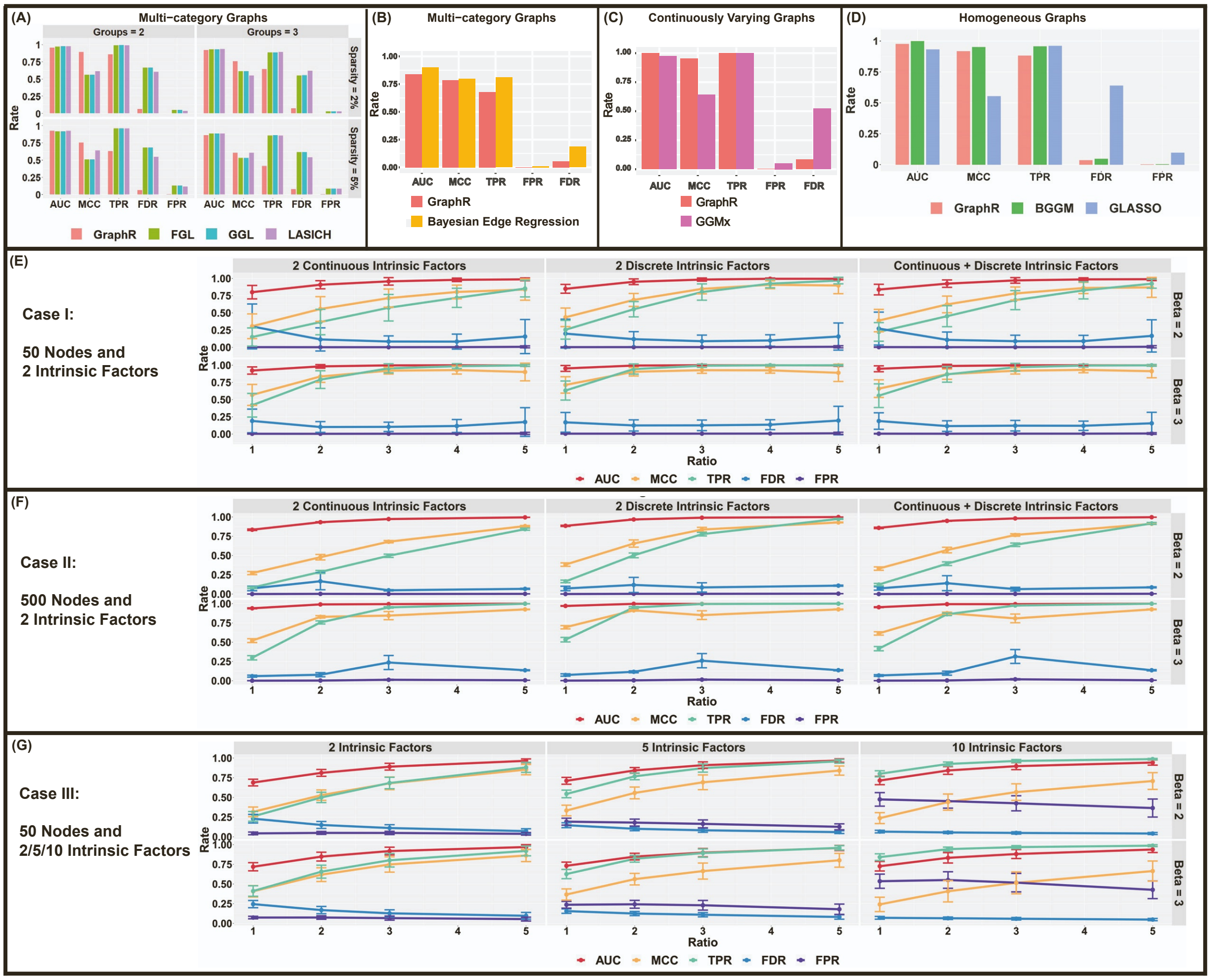

Figure S1: **Supplementary results on simulation study, related to Figure 3.** **(A)** shows selection performance in multi-category graphs setting with varying number of groups and sparsity. **(B)** summarizes the simulation results in multi-category graphs setting. GraphR is compared with Bayesian edge regression [15], a MCMC-based method in a low-dimensional scenario where $n = 100$, $p = 20$, $q = 2$ and sparsity level at 5%. **(C)** shows selection performance in homogenuous setting for 5% sparsity level. **(D)** summarizes the simulation results in continuously-varying graphs setting. GraphR is compared with GGMx [10], a MCMC-based method in a low-dimensional scenario where $n = 100$, $p = 20$, $q = 1$ and sparsity level at 5%. **E** illustrates the selection performance of edges in directed acyclic graphs with $p = 50, q = 2$, and varying intrinsic factor types and n/pq ratios. **E** illustrates the selection performance of edges in directed acyclic graphs with $p = 500, q = 2$, and varying intrinsic factor types and n/pq ratios. **F** illustrates the selection performance of edges in directed acyclic graphs with $p = 50$, and varying number of intrinsic factor and n/pq ratios.

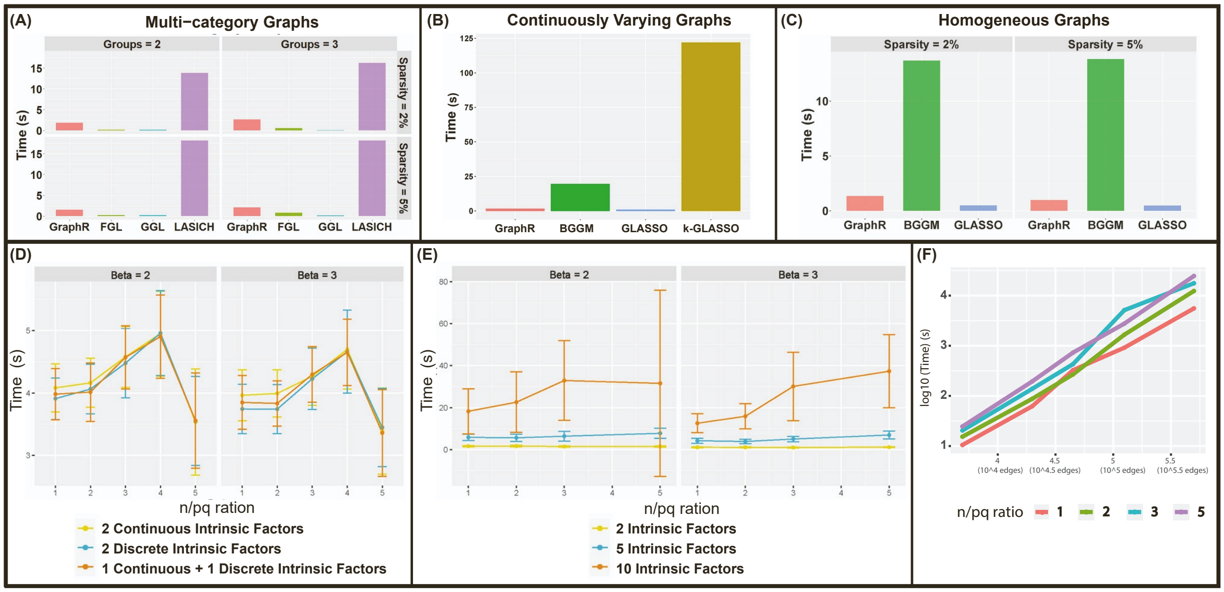

Figure S2: **Supplementary results on computation time under simulation settings, related to Figure 3.** **(A)** shows computation time in multi-category graphs setting with varying number of groups and sparsity. **(B)** shows computation time in continuously-varying graphs setting. **(C)** shows computation time in homogenuous setting for varying sparsity level. **(D)** illustrates computation time for directed acyclic graphs with $p = 50, q = 2$, and varying intrinsic factor types and n/pq ratios, and effect size. **(E)** illustrates computation time for directed acyclic graphs with $p = 50$, and varying number of intrinsic factor and n/pq ratios, and effect size. **(F)** shows computation times in the directed acyclic graph setting varying with the number of edges and n/pq ratios. The x-axis and y-axis represent log10 of the number of edges and computation time respectively, with different colors representing various n/pq ratios.

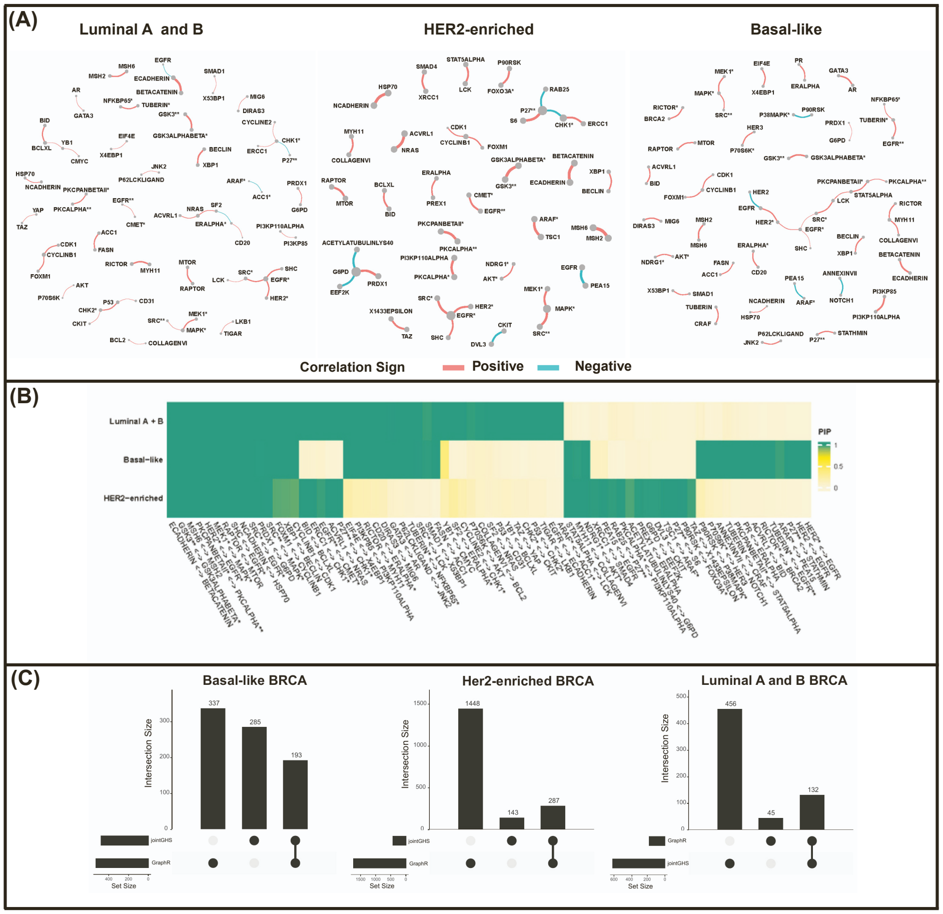

Figure S3: **Supplemental analysis of proteomic network-based characterization of intrinsic subtypes of breast cancer, related to Figure 4. (A)** shows networks of selected protein pairs in Luminal A+B, Her2-enriched and Basal-like BRCA respectively. The widths of edges are proportional to partial correlations. The sizes of nodes are proportional to connectivity degrees which are defined as the sum of magnitudes of the partial correlations. Sign of partial correlations are represented by color with red being positive and blue being negative. **(B)** shows heatmap of posterior inclusion probability (PIP) of selected edges in each PAM50 subtype of BRCA. We have compared with jointGHS which demonstrates the highest MCC other than GraphR in the multi-category graphs simulation setting. The upset plot in **(C)** summarizes the finding from intrinsic sybtypes of BRCA data between `GraphR` and jointGHS. For basal-like breast cancer, `GraphR` and jointGHS identify 193 edges in common. `GraphR` uniquely identifies 337 edges that are not selected by jointGHS, while jointGHS uniquely identifies 285 edges that are not selected by `GraphR`. For the Luminal A and B BRCA subtypes, both methods agree on 132 edges. `GraphR` uniquely identifies 45 edges, whereas jointGHS identifies 456 edges that are not found by `GraphR`. In the HER2-enriched BRCA subtype, `GraphR` and jointGHS agree on 287 edges. `GraphR` uniquely identifies 1448 edges, while jointGHS uniquely identifies 143 edges.

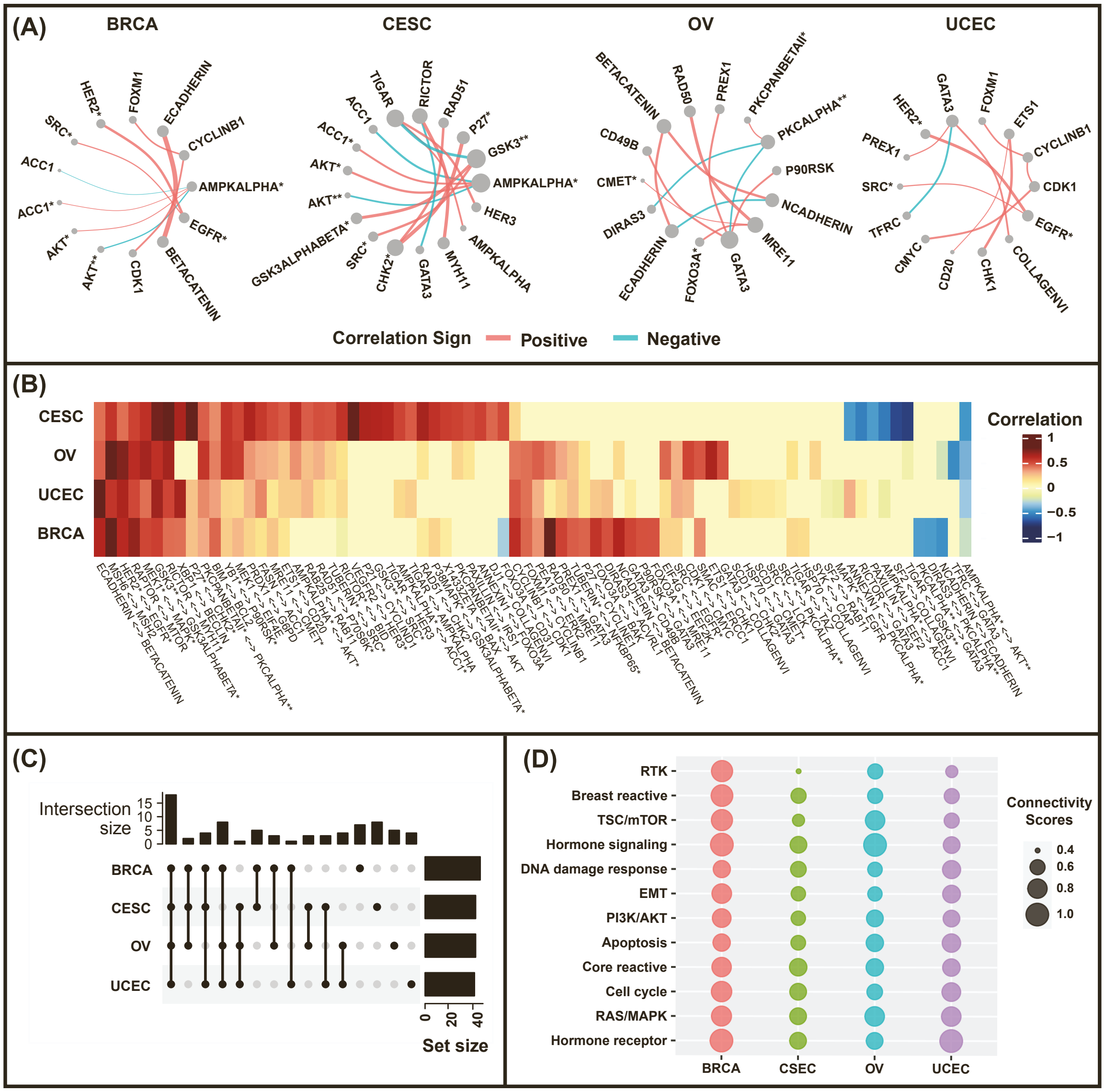

Figure S4: **Pan-Cancer Proteomic Network-based Characterization of Breast and Gynecologic Cancers, related to Figure 4.** The results are based on BRCA and three types of gynecologic cancers including cervical squamous cell carcinoma and endocervical adenocarcinoma (CESC), ovarian serous cystadenocarcinoma (OV) and uterine corpus endometrial carcinoma (UCEC). **(A)** Cancer-specific networks for proteins with top five connectivity degrees. **(B)** Heatmap of partial correlations between protein pairs for each cancer type. We only include significant protein pairs which are highly correlated with FDR based p-values < 0.01 and $|\text{partial correlation}| > 0.45$ for at least one type of cancer. **(C)** Upset plot for numbers of significant connections within or across cancer types based on the protein pairs included in (B). **(D)** Connectivity scores of pathways for each cancer type. Pathways are ordered based on the clustering of connectivity scores.

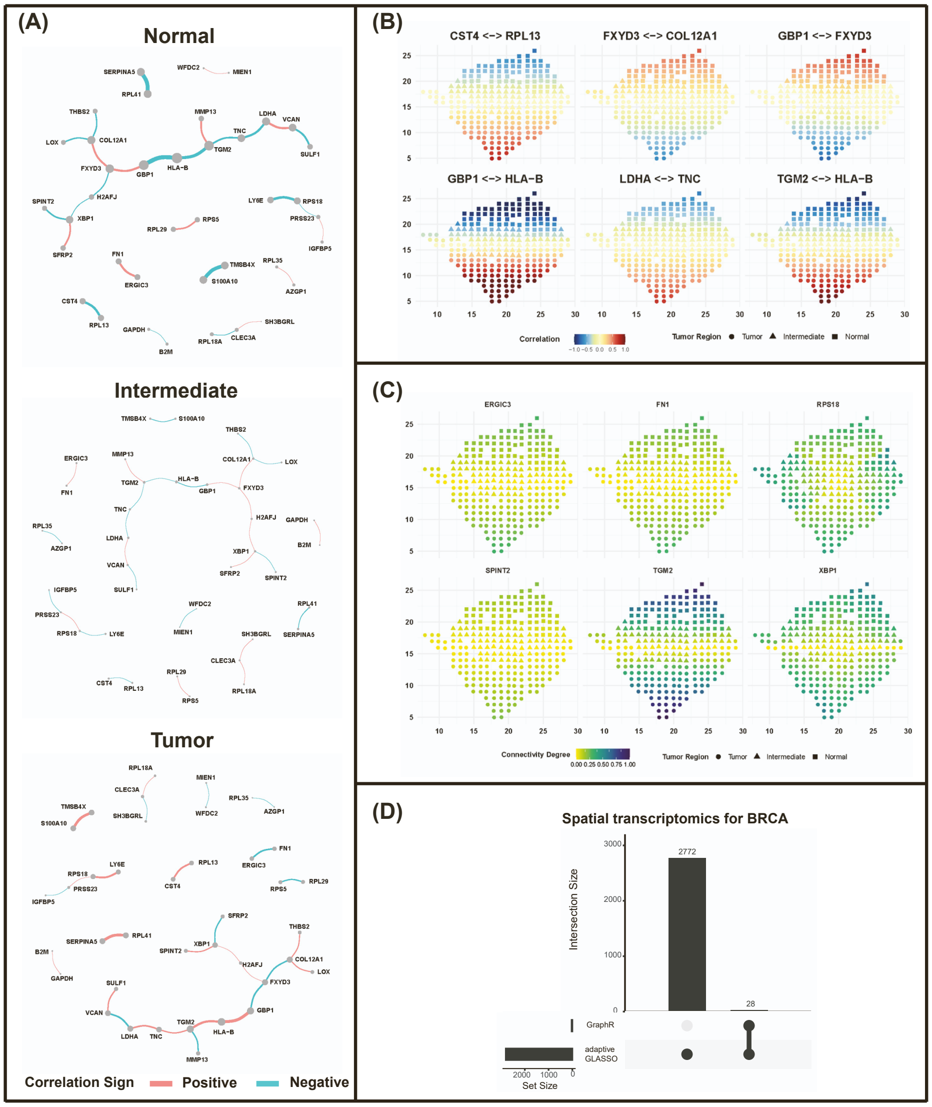

Figure S5: **Additional results for analysis of spatial transcriptomics data, related to Figure 6.** **(A)** shows the whole networks w.r.t. each spatial regions. The edges are proportional to the weighted average of partial correlations and nodes are proportional to the weighted connectivity degrees. We also display more spatial patterns of partial correlations**(B)** and connectivity degrees **(C)** for gene and gene pairs. The color bar indicates the values of correlations and connectivity degrees while shapes of point representing the spatial region. We implemented an adaptive GLASSO based approach for comparative study of findings from sptial transcriptomics data. For each spot, neighboring spots within a pre-specified radius of 1 are included, and we apply GLASSO within that neighborhood. `GraphR` identifies 28 edges, all of which are also detected by the adaptive GLASSO. However, the adaptive GLASSO identifies an addition 2,772 edges as significant, indicating a potential high rate of false positives. We summarize these results through the upset plot in **(D)**.

References

[1] Baladandayuthapani, V., Ji, Y., Talluri, R., Nieto-Barajas, L. E., and Morris, J. S. (2010). Bayesian random segmentation models to identify shared copy number aberrations for array cgh data. *Journal of the american statistical association*, 105(492):1358–1375.

[2] Danaher, P., Wang, P., and Witten, D. M. (2013). The Joint Graphical Lasso for Inverse Covariance Estimation Across Multiple Classes. *Journal of the Royal Statistical Society Series B: Statistical Methodology*, 76(2):373–397.

[3] Ji, Y., Yin, G., Tsui, K.-W., Kolonin, M. G., Sun, J., Arap, W., Pasqualini, R., and Do, K.-A. (2007). Bayesian mixture models for complex high dimensional count data in phage display experiments. *Journal of the Royal Statistical Society: Series C (Applied Statistics)*, 56(2):139–152.

[4] Li, Z., Mccormick, T., and Clark, S. (2019). Bayesian joint spike-and-slab graphical lasso. In *International Conference on Machine Learning*, pages 3877–3885. PMLR.

[5] Lingjærde, C., Fairfax, B. P., Richardson, S., and Ruffieux, H. (2022). Scalable multiple network inference with the joint graphical horseshoe. *arXiv preprint arXiv:2206.11820*.

[6] Liu, H., Roeder, K., and Wasserman, L. (2010). Stability approach to regularization selection (stars) for high dimensional graphical models. *Advances in neural information processing systems*, 23.

[7] Meinshausen, N. and Bühlmann, P. (2010). Stability selection. *Journal of the Royal Statistical Society: Series B (Statistical Methodology)*, 72(4):417–473.

[8] Morris, J. S., Brown, P. J., Herrick, R. C., Baggerly, K. A., and Coombes, K. R. (2008). Bayesian analysis of mass spectrometry proteomic data using wavelet-based functional mixed models. *Biometrics*, 64(2):479–489.

[9] Ni, Y., Stingo, F. C., and Baladandayuthapani, V. (2019). Bayesian Graphical Regression. *Journal of the American Statistical Association*, 114(525):184–197.

[10] Ni, Y., Stingo, F. C., and Baladandayuthapani, V. (2022). Bayesian Covariate-Dependent Gaussian Graphical Models with Varying Structure. *J. Mach. Learn. Res.*, 23(1).

[11] Peterson, C., Stingo, F. C., and Vannucci, M. (2015). Bayesian inference of multiple gaussian graphical models. *Journal of the American Statistical Association*, 110(509):159–174.

[12] Saegusa, T. and Shojaie, A. (2016). Joint estimation of precision matrices in heterogeneous populations. *Electronic Journal of Statistics*, 10(1):1341 – 1392.

[13] Storey, J. D. (2003). The positive false discovery rate: a bayesian interpretation and the q-value. *The Annals of Statistics*, 31(6):2013–2035.

[14] Titsias, M. and Lázaro-Gredilla, M. (2011). Spike and slab variational inference for multi-task and multiple kernel learning. *Advances in neural information processing systems*, 24.

[15] Wang, Z., Baladandayuthapani, V., Kaseb, A. O., Amin, H. M., Hassan, M. M., Wang, W., and Morris, J. S. (2022). Bayesian Edge Regression in Undirected Graphical Models to Characterize Interpatient Heterogeneity in Cancer. *Journal of the American Statistical Association*, 117(538):533–546.

[16] Yang, X., Gan, L., Narisetty, N. N., and Liang, F. (2021). Gembag: Group estimation of multiple bayesian graphical models. *Journal of machine learning research*, 22(54):1–48.

[17] Zhang, J. and Li, Y. (2022). High-Dimensional Gaussian Graphical Regression Models with Covariates. *Journal of the American Statistical Association*, 0(0):1–13.
