# Supplementary material for: A probabilistic modeling framework for genomic networks incorporating sample heterogeneity
Source: Cell Rep Methods. 2025 Feb 14;5(2):100984. doi: 10.1016/j.crmeth.2025.100984 (PMC11955270; doi:10.1016/j.crmeth.2025.100984)
Supplement: Document S2. Article plus supplemental information [file mmc2.pdf]

Cell Reports Methods

Article

A probabilistic modeling framework for genomic networks incorporating sample heterogeneity

Graphical abstract

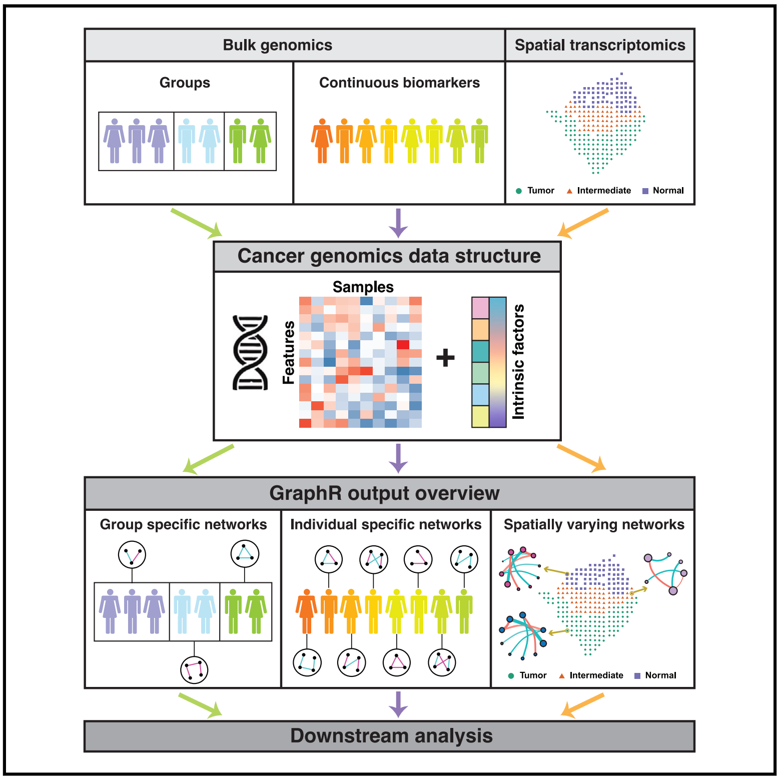

Highlights

- A Bayesian modeling framework that incorporates heterogeneity in network estimation
- GraphR infers subtype- or individual-specific genomic and spatial genomics networks
- GraphR accurately recovers network structure and outperforms competing methods
- GraphR employs scalable algorithms, implemented in an R package and a Shiny App

Authors

Liying Chen, Satwik Acharyya, Chunyu Luo, Yang Ni, Veerabhadran Baladandayuthapani

Correspondence

veerab@umich.edu

In brief

Chen et al. develop a graphical regression model (GraphR), which is a supervised Bayesian modeling framework for network inference that accounts for sample heterogeneity at different scales. GraphR is a computationally efficient method that estimates spatial and genomic networks, revealing inter- and intra-sample network structures.

Chen et al., 2025, Cell Reports Methods 5, 100984
February 24, 2025 © 2025 The Author(s). Published by Elsevier Inc.
https://doi.org/10.1016/j.crmeth.2025.100984

CellPress

Cell Reports Methods

CellPress
OPEN ACCESS

Article

A probabilistic modeling framework for genomic networks incorporating sample heterogeneity

Liying Chen,[1,5] Satwik Acharyya,[2,5] Chunyu Luo,[3] Yang Ni,[4] and Veerabhadran Baladandayuthapani[1,6,*]
[1]Department of Biostatistics, University of Michigan, Ann Arbor, MI, USA
[2]Department of Biostatistics, University of Alabama at Birmingham, Birmingham, AL, USA
[3]Division of Biostatistics, University of Pennsylvania, Philadelphia, PA, USA
[4]Department of Statistics, Texas A&M University, College Station, TX, USA
[5]These authors contributed equally
[6]Lead contact
*Correspondence: veerab@umich.edu
https://doi.org/10.1016/j.crmeth.2025.100984

MOTIVATION Genomic networks aim to decipher the intricate relationships between molecular entities in many disease systems. However, most existing network modeling approaches assume homogeneity within and across samples, limiting their broad applicability in heterogeneous disease contexts. For example, it is well-established that cancer is a heterogeneous disease, characterized by inter- and intra-tumoral as well as spatial heterogeneity in the tumor microenvironment. We reason and show that ignoring the potential impact of this heterogeneity can lead to biased results in network estimation via Simpson's paradox. To this end, we present a method, GraphR, that enables accurate genomic network estimation while incorporating intrinsic structural heterogeneity at a sample-specific level.

SUMMARY

Probabilistic graphical models are powerful tools to quantify, visualize, and interpret network dependencies in complex biological systems such as high-throughput -omics. However, many graphical models assume sample homogeneity, limiting their effectiveness. We propose a flexible Bayesian approach called graphical regression (GraphR), which (1) incorporates sample heterogeneity at different scales through a regression-based formulation, (2) enables sparse sample-specific network estimation, (3) identifies and quantifies potential effects of heterogeneity on network structures, and (4) achieves computational efficiency via variational Bayes algorithms. We illustrate the comparative efficiency of GraphR against existing state-of-the-art methods in terms of network structure recovery and computational cost across multiple settings. We use GraphR to analyze three multi-omic and spatial transcriptomic datasets to investigate inter- and intra-sample molecular networks and delineate biological discoveries that otherwise cannot be revealed by existing approaches. We have developed a GraphR R package along with an accompanying Shiny App that provides comprehensive analysis and dynamic visualization functions.

INTRODUCTION

Network analyses are powerful tools for deciphering and predicting underlying structures and dynamics in complex biological systems. Typically, complex biological systems consist of a high-dimensional array of features including genes, proteins, and other molecules that exhibit intricate interactions.[32,37,65] The global structures and cumulative behavior of the systems cannot be inferred solely from partial factors of the system.[8] The increasing availability of high-throughput molecular data has fueled an explosive interest in network-based approaches over the past few decades.[9,26] Specifically, biological networks capture the complex interactions between and within genomic molecular entities, enabling a holistic understanding of cellular functioning, organization, and underlying etiology of disease; examples include protein-protein interaction,[32] gene co-expression networks,[65] and gene regulatory networks.[37] Such networks, synonymous with graphs, are compact representations of genomic interactions where features are nodes interconnected by edges (links) between them. These graphs provide a conceptual and intuitive framework to study interactions

This is an open access article under the CC BY license (http://creativecommons.org/licenses/by/4.0/).

across multiple omics and spatial omics fields such as genomics, transcriptomics, and proteomics.[87]

Probabilistic graphical models (PGMs) are a common class of approaches to infer networks of complex systems that allow the measurement of uncertainty and provide probabilistic reasoning.[40] A PGM can be represented by a joint probability distribution over a high-dimensional space based on a graph that compactly encodes dependence structures among a set of nodes (variables), and the associated conditional distributions.[40] Furthermore, the associated probability distributions over networks formalize uncertain knowledge regarding the quantitative dependencies between variables while also enabling the incorporation of prior knowledge, which further facilitates the decision-making process.[62] Undirected graphical models are a widely used subclass of PGMs, with edges in the graph representing conditional independence between two nodes. Specifically, if nodes follow a Gaussian distribution, one obtains Gaussian graphical models (GGMs). GGMs represent multivariate Gaussian distributions with a sparse precision matrix, where a zero entry signifies conditional independence.[21] This framework extends conditional independence to a partially uncorrelated structure, which measures the association between two random variables after adjusting for the effects of all other variables.[42,69]

GGMs have undergone intense methodological and computational developments in recent years.[4,25,26] This progress has facilitated their widespread use across various biomedical domains, especially in genomics research.[22,44] Despite their widespread use, most of the GGM-based methods make a fundamental assumption of *homogeneity*, i.e., samples arise from a common population implying that the samples are independent and identically distributed.[25,55,57] This assumption may not be valid in many modern applications that generate heterogeneous data. One of the canonical examples that arise in cancer research is *tumor heterogeneity*.[95] Tumors usually present substantial inter- and intra-tumor heterogeneity, such as distinct genotypes and phenotypes of different regions within a single tumor,[43] across the same type (or subtype) of cancers[3] or spatial heterogeneity.[93] Tumor heterogeneity plays a critical role in oncology research and clinical practice, providing valuable insights into disease progression, therapeutic selection, and clinical outcomes of patients; for example, breast cancer subtype-based systemic therapy selection,[88] EGFR mutations and anti-EGFR therapies in lung cancer,[84] stem cell gene expression programs in leukemia,[23] and intra-tumor heterogeneity levels and cancer evolution in human glioblastoma.[78] Tumor heterogeneity manifests itself at multiple intrinsic levels such as sub-population or sample-specific levels. Ignoring the potential impact of these intrinsic heterogeneity factors may lead to biased results that fail to reflect the true nature of associations between molecular markers, primarily due to Simpson's paradox.[14] A simple illustration of Simpson's paradox in graphical models is provided in the results section where the assumption of homogeneity leads to spurious associations and appropriate accounting for intrinsic heterogeneity can reveal true biological signals. Hence graphical models that incorporate various types and scales of heterogeneity and allow for network inference at the sub-population or individual sample level have the potential to uncover the true biological structures that can considerably aid precision medicine endeavors.[75]

In recognition of this fact, a few methods have been developed over the last few years for multi-category or individualized network estimation. For multi-category network estimation, wherein samples are grouped based on discrete factors such as cancer types, and observations within each group are assumed to be independent and identically distributed (i.i.d.), a variety of approaches have been proposed that enable the analysis to account for heterogeneity among groups while leveraging shared information across them. From a frequentist standpoint,[20] introduce a penalized likelihood approach with fused lasso or group lasso penalties, and[73] employ a Laplacian shrinkage penalty and later extend this with a Laplacian penalty based on hierarchical clustering. In Bayesian settings,[46] extend the group and fused graphical lasso by incorporating a Gaussian spike-and-slab prior in their multiple-network model, while several other methodological frameworks[27,92] applied spike-and-slab priors with Laplace distributions[48] that propose a joint graphical horseshoe estimator for multiple-network inference. However, these methods can only incorporate discrete covariates and cannot handle continuous covariates, covariate combinations (discrete and continuous), and spatial covariates (see Table S1).

In terms of individualized network estimation methods, they can broadly be classified into two categories: unsupervised ("top-down") and supervised ("bottom-up") approaches. Unsupervised approaches in the top-down direction typically involve de-convolution of population-level networks to infer sample-specific networks, implemented using unsupervised algorithms.[32,41,50] However, the top-down approaches do not allow assessments of the direct impact of heterogeneity measures on network edges in a straightforward manner and thus fail to identify and quantify potential confounders, due to a lack of inferential procedures for individual networks. More recently, bottom-up approaches have been developed to alleviate this to some degree, as they allow a direct incorporation of heterogeneity measures and are implemented using supervised algorithms. Within a frequentist framework, sample-specific graphs are inferred based on penalty functions, which are not geared to provide uncertainty quantification.[49,94] Supervised Bayesian approaches allow the estimation and inference of individualized networks[63,64,89] primarily through computationally intensive sampling-based approaches, which stymies their application to high-dimensional genomic datasets. Furthermore, most, if not all, methods are not designed to incorporate multiple number and scales heterogeneous intrinsic factors and thus are not broadly applicable (see Table S1).

To address these challenges, we develop a general, flexible, and computationally scalable Bayesian framework for estimating network structures incorporating multiple types and scales of intrinsic heterogeneity, called graphical regression (GraphR). GraphR employs a regression-based formulation in the spirit of previous works[63,89] to estimate graph structures in GGMs where precision matrices are linear functions of heterogeneity features that are represented by intrinsic factors (as covariates in regression setting). GraphR takes input in the form of a feature matrix (e.g., genes or proteins expression) and samples along

with intrinsic factors in diverse combinations, allowing for incorporation of multiple types of heterogeneity (Figure 1). Additionally, GraphR can also identify and quantify the potential effects of intrinsic factors on graph structures, enhancing the understanding and interpretation of underlying mechanisms at a sample-specific level. To demonstrate the performance and versatility of GraphR, we present comprehensive benchmarking using synthetic simulated datasets under a range of scenarios and show that the GraphR method is robust and outperforms several existing network-based methods, in terms of structural recovery in the presence of heterogeneity. We further apply GraphR to four diverse breast cancer-related datasets that include proteogenomics and spatial transcriptomics data, with intrinsic heterogeneity factors being breast cancer (sub)types, dedifferentiation stemness levels, and spatial locations. Our key findings include (1) identification of key proteomic regulators and functional pathways in breast cancer subtypes, as well as across a spectrum of gynecological cancers; (2) detection of protein-protein interactions and functional pathways modulated by different levels of cellular dedifferentiation; and (3) spatially varying gene co-expression patterns across the breast cancer tumor microenvironment—highlighting the method's versatility.

Finally, we have developed an R-package and an accompanying Shiny App that is easily accessible to the scientific and research community to obtain, inspect, rerun, and more importantly apply the methods on their own datasets, without coding experience and implementations.

RESULTS

Our results are organized as follows. First, we motivate the need for GraphR through an illustration of Simpson's paradox in graphical models.

Second, we present the operating characteristics of GraphR using synthetic datasets and compare it with other related graphical modeling approaches across multiple scenarios. Finally, we apply GraphR to four diverse datasets in breast cancer (BRCA) to characterize different aspects of heterogeneity in proteogenomic networks. BRCA is the most common cancer among women in the United States, with around 3 million newly diagnosed cases and 43K deaths in 2022.[76] Accumulating evidence suggests that multiple factors affect the choice of treatment for breast cancer, such as subtypes and stages of cancer, as well as individual patient considerations.[19,88] To better dissect breast cancer heterogeneity, we utilize GraphR to explore the proteomic characterization of intrinsic subtypes of BRCA, assess differences and commonalities in proteomic networks across pan-gynecological cancers, and investigate effects of dedifferentiation levels (stemness indices) and the effect of the tumor microenvironment using BRCA spatial transcriptomic data.

Illustration of Simpson's paradox in graphical models

We motivate the development of GraphR using an illustration of Simpson's paradox in graphical models. Simpson's paradox broadly refers to a scenario where trends between two variables reverse or disappear after adjusting for some other variables. To illustrate the key principle, we present a simple example in a graphical model setting with three nodes. Assume we have samples from two heterogeneous groups with distinct conditional independence structures (Figure 2, left panel) where the partial correlation between nodes A and B (conditioned on node C) is negative (partial correlation = -0.61; shown in black) in the first group and positive (partial correlation = 0.56; shown in red) in the second group (the details of construction are presented STAR Methods). However, if we treat all samples as homogeneous (i.e., coming from a single group), the partial correlation between the two nodes is obfuscated (partial correlation = 0.20; Figure 2, right panel). This demonstrates that even for a simple three-node graph, ignoring discrete heterogeneity leads to erroneous conclusions. In more complex scenarios, such as existence of multiple confounders at different scales (categorical and continuous), the inference of graphical structures among multiple genomic features is more challenging and motivates the development of graphical models that incorporate this additional axis of information to construct heterogeneous networks (as shown in Figure 1).

GraphR accurately recovers network structures in presence of sample heterogeneity

To demonstrate the operating characteristics of GraphR, we provide simulation results under two broad scenarios: undirected graphs with unordered pairs of nodes and directed graphs where directions of edges are *a priori* known. Our core hypothesis is that accounting for different scales of intrinsic factor-based heterogeneity enables better network structural recovery for both undirected and directed graphs. For the undirected setting, we consider the following three cases: (1) multiple categories (discrete heterogeneity), (2) with intrinsic factors being continuous variables (continuous heterogeneity), and (3) a homogeneous population (positive control simulation). We compare our approach to several relevant methods: (1) Bayesian Gaussian graphical models (BGGM[57]), (2) graphical lasso (GLASSO[25]), (3) fused graphical lasso (FGL[20]), (4) group graphical lasso (GGL[20]), (5) Laplacian shrinkage for inverse covariance matrices from heterogeneous populations (LASICH[73]), (6) joint graphical horseshoe (jointGHS[48]), and (7) kernel graphical lasso (K-GLASSO[49]). An overview of the competing methods is provided in Table S1. We further illustrate the operating characteristics of GraphR with varying dimensions of features (50–500), intrinsic factors (2–10), and sample sizes (100–5000) through datasets generated from directed acyclic graphs (DAGs).

To evaluate the performance of network recovery in both settings, we give comparative metrics[1]: area under the receiver operating characteristic curve (ROC) curve (AUC),[2] true positive rate (TPR),[3] false positive rate (FPR),[4] Matthew's correlation coefficient (MCC), and[5] false discovery rate (FDR). MCC considers all four components of the recovery rates: true positives (TPs), true negatives (TNs), false positives (FP), and false negatives (FN) $\left[\mathrm{MCC} = (\mathrm{TP} \times \mathrm{TN} - \mathrm{FP} \times \mathrm{FN}) / \sqrt{(\mathrm{TP}+\mathrm{FP})(\mathrm{TP}+\mathrm{FN})(\mathrm{TN}+\mathrm{FP})(\mathrm{TN}+\mathrm{FN})}\right]$, and provides a comprehensive evaluation of the selection performance. Further details about simulation design are provided in the STAR Methods.

Undirected graphs

Under discrete heterogeneity, as shown in Figure 3A, GraphR outperforms all competitive methods in terms of MCC

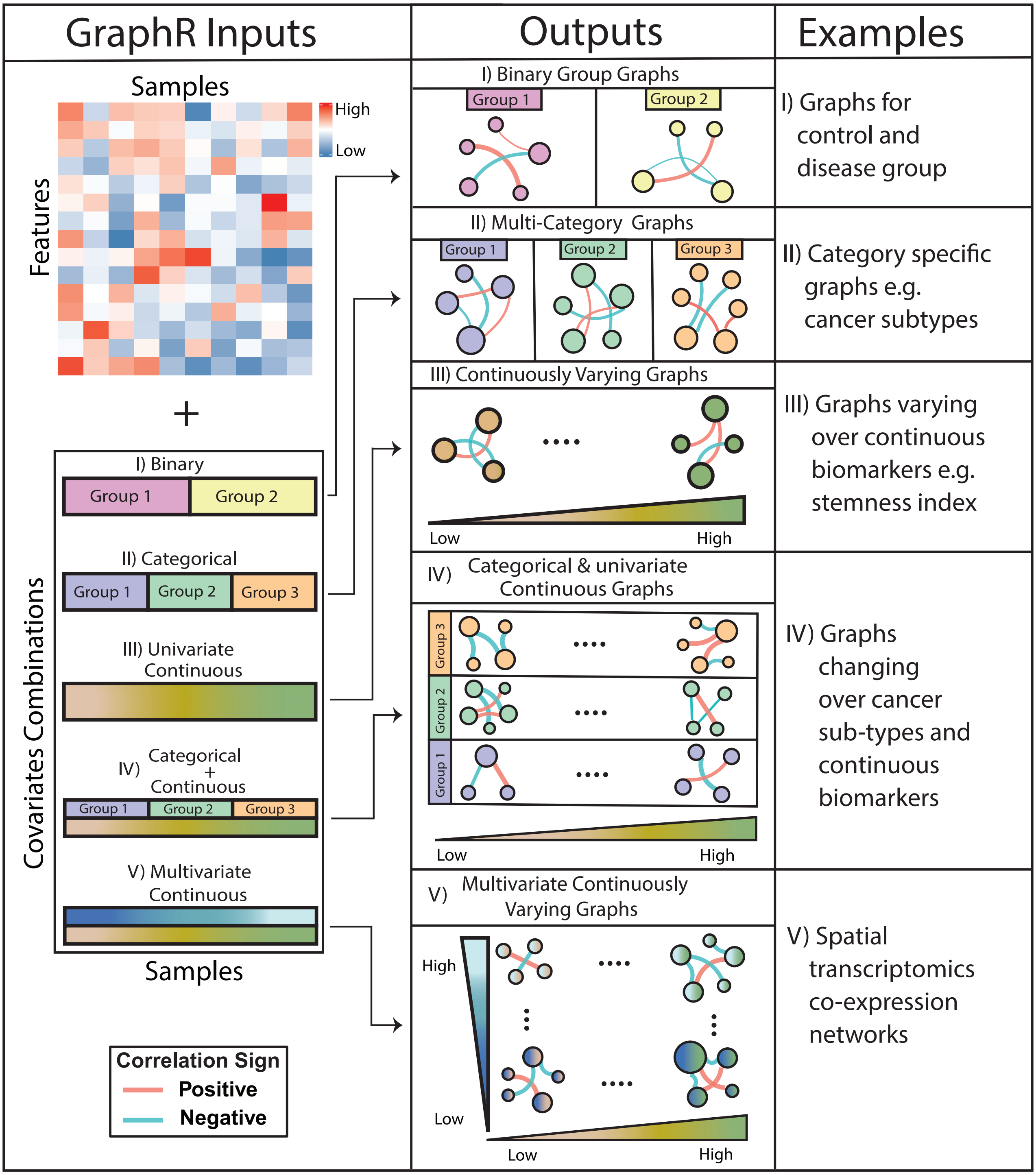

Figure 1. Overview of GraphR

The input for GraphR is a feature matrix (e.g., gene or protein expression) and intrinsic factors reflecting heterogeneous structure (e.g., cancer types, dedifferentiation levels, or spatial domains). The GraphR method can infer individualized graphs while accommodating different combinations of intrinsic factors: (1) binary intrinsic factors, such as control and disease groups leading to binary group graphs; (2) categorical intrinsic factors, e.g., multi-category graphs for cancer subtypes; (3) univariate continuous intrinsic factors, e.g., graphs varying over dedifferentiation levels; (4) combinations of categorical and continuous intrinsic factors, such as graphs changing over cancer subtypes and continuous biomarkers; and (5) multivariate continuous intrinsic factors such as application to spatial transcriptomics data resulting in graphs are changing over the whole spatial domain.

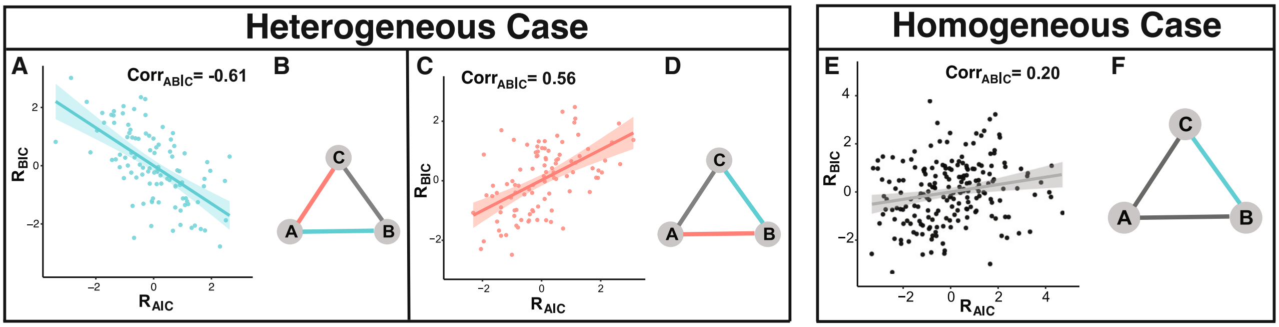

Figure 2. Illustration of Simpson's paradox in graphical models

Samples are drawn from two heterogeneous groups with distinct conditional independence structures.

The heterogeneous case considers heterogeneous group effects. (A) and (C) of the left panel denote point plots of residuals from linear regression of node A with node C and node B with node C, respectively, with linear regression as the smoothing function (green and red line respectively). The observed partial correlation is −0.61 and 0.55 in the two groups.

(B) and (D) of the left panel show estimated conditional independence structures from GraphR for two groups. Colors of edges represent signs of partial correlation, with green and red being negative and positive, respectively, and gray indicating no connection.

The homogeneous case shows results when treating all samples homogeneously.

(A)–(D) represent association between residuals from linear regression of node A with node C and node B with node C. The observed partial correlation is 0.20. All other settings are the same as the heterogeneous case. (E)–(F) in the right panel characterize the estimated conditional independence structures from GraphR and rest of the settings are same as the heterogeneous case.

(GraphR: 0.892; FGL: 0.559; GGL: 0.56; LASICH: 0.611, jointGHS: 0.76) and a comparable AUC (GraphR: 0.953; FGL: 0.765; GGL: 0.763; LASICH: 0.973; jointGHS: 0.94). In comparison with GraphR, FGL, GGL, and LASICH have high values of TPR at the expense of higher FDR and FPR, whereas jointGHS has a well-controlled FDR at the cost of a significantly lower TPR. This pattern is well captured by MCC, as it provides an overall assessment considering other discovery rates. Notably, GraphR performs best in case of individual-specific precision matrices (i.e., continuous intrinsic factor-based heterogeneity). Figure 3B shows that GraphR achieves the highest MCC (GraphR: 0.955, BGGM: 0.354, GLASSO: 0.316 and k-GLASSO: 0.333) and AUC (GraphR: 0.998, BGGM: 0.838, GLASSO: 0.824 and k-GLASSO: 0.878) among other methods. We further conduct simulations to compare GraphR with MCMC-based methods, such as Bayesian edge regression[89] and GGMx,[64] in both discrete and continuous intrinsic factor-based settings. The evaluation metrics and computational times are detailed in STAR Methods and Figures S1 and S2. In the end, we assess the performance of GraphR for homogeneous graphs in Figure 3C where GraphR attains the highest MCC (GraphR: 0.955; BGGM: 0.951; GLASSO: 0.644) and marginally lower AUC (GraphR: 0.989; BGGM: 1; GLASSO: 0.993). The homogeneous case serves as positive control with respect to other simulation settings. Detailed values of other discovery rates and results from different simulation settings are provided in the STAR Methods and Figures S1 and S2.

DAGs

We further demonstrate the operating characteristics of GraphR in directed graph scenarios with 50 nodes ($p = 50$), two intrinsic heterogeneity factors ($q = 2$), and varying sample sizes ($n = 100, 200, 300, 400, 500$). GraphR generally performs better with increasing ratio between n and pq and effect size β (Figures 3D–3F; Figure S1). However, FDR increases when n over pq ratio reaches 5 (continuous: 0.174; discrete: 0.196; continuous and discrete: 0.156) compared with the case when n over pq ratio 4 (continuous: 0.117; discrete: 0.136; continuous and discrete: 0.120), leading to moderate decrease in MCC from (0.929, 0.926, 0.932) to (0.901, 0.89, 0.913) with settings in Figures 3D–3F, respectively.

In summary, the GraphR method is robust and performs better in terms of structural recovery in presence of intrinsic factor-based heterogeneity along with well-controlled FDR across multiple scenarios such as undirected and directed graphs. Under heterogeneous structure, GraphR method achieves higher power than other methods with the lowest FDR and FPR, indicating the importance of incorporate heterogeneity in network estimation. Notably, GraphR performs better in terms of MCC, which indicates that GraphR can effectively balance the trade-off between both true and false recovery rates as compared with existing methods.

Proteomic network-based characterization of intrinsic subtypes of BRCA

We focus on characterization of the shared and subtype-specific proteomic networks of intrinsic subtypes of BRCA.[68] These subtypes have demonstrated distinct genomic features and have been established as crucial prognostic factors in treatment selection and clinical outcomes[70,90]; however, their proteomic networks have been under-explored. To this end, we apply GraphR to 190 proteins obtained from 626 BRCA patients from The Cancer Genome Atlas (TCGA[90]) across three subtypes (as discrete factors): Luminal A and B ($n = 393$), Her2-enriched ($n = 75$), and Basal-like ($n = 158$). Proteomics data are obtained and normalized from The Cancer Proteome Atlas[45] through reverse phase protein arrays (RPPAs) to quantify proteins or phosphoproteins that are involved in multiple functional and signaling pathways such as apoptosis, DNA damage response, and cell cycle.[2]

We aim to estimate the cancer subtype-specific networks and only include protein pairs belonging to different isoforms

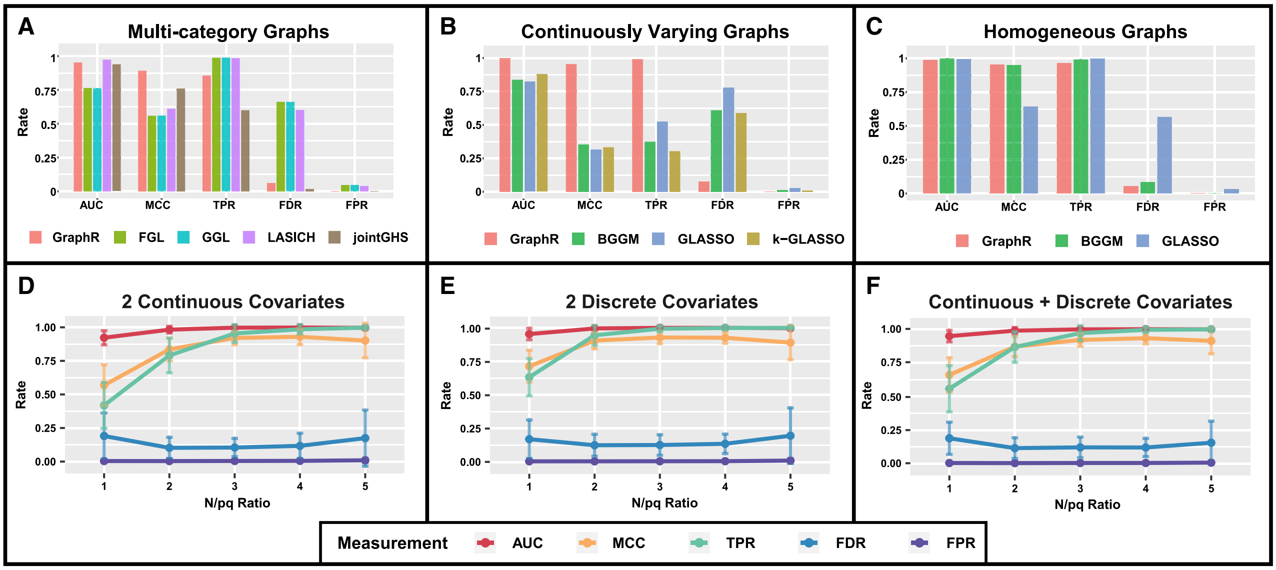

Figure 3. Simulation results

For each setting, we report the mean values of area under the ROC curve (AUC), Matthews correlation coefficient (MCC), true positive rate (TPR), false positive rate (FPR), and false discovery rate (FDR) based on 50 repetitions to evaluate the selection performance.

(A)–(C) represent comparison of selection (recovery) performance among GraphR and several other methods under the undirected graphical settings. We consider three cases here, where subjects are drawn from two different categories (A) or each subject is a heterogeneous (B) or a homogeneous population (C). Seven relevant methods are used for comparison, involving BGGM, GLASSO, FGL, GGL, LASICH, jointGHS, and K-GLASSO.

(D)–(F) show mean selection performance with ±1.96 × standard deviation (SD) for directed acyclic graphs with number of nodes $p = 50$ and number of intrinsic factors q = 2. The x axis is defined as the ratio between sample size n and p*q, ranging from 1 to 5. Effect size β is set to be 3 while the types of two intrinsic factors being continuous only (D), or discrete only (E), or continuous and discrete covariates (F).

while showing significantly strong partial correlation (FDR-based p-value $\langle$ 0.01 and $|$ partial correlation $| \geq 0.4$) for at least one group. Proteins with top 5 connectivity degrees are used to plot cancer subtype-specific networks in Figure 4A (the full networks across all proteins are shown in Figure S3). We find several proteins are conserved across groups such as phosphorylated EGFR, i.e., EGFR* (Note: Throughout this text, we denote phosphorylated forms of proteins with an asterisk [*]) for Luminal A + B and Her2-enriched with relatively high co-expression with Her2*, Shc, and Src*. Figure 4B shows the heatmap of partial correlations between selective protein pairs of three groups and the corresponding heatmap of posterior inclusion probabilities (PIP) is provided in Figure S3. The upset plot in Figure 4C demonstrates the numbers of significant connections within or across BRCA groups. Overall, 15 connections are identified as significant across all three groups while 13, 12, and 14 connections only showed significance in Basal-like, Her2-enriched, and Luminal A and B, respectively, indicating the similarities and differences for each group of BRCA. In the heatmap, we can observe high partial correlations of proteins belonging to the same family, e.g., MSH2 and MSH6. Furthermore, we also find that E-cadherin and β-catenin are positively correlated in all groups of BRCA with partial correlation being 0.69, 0.69, and 0.98 in Basal-like, Her2-enriched, and Luminal A and B BRCA. We observe negative partial correlations between p27*-Chk1* in Her2-enriched and Luminal A and B BRCA with moderate and weak magnitude respectively (Her2-enriched: $\rho = -0.42$; Luminal A and B: $\rho = -0.19$). Interestingly, we do not identify this negative partial correlation in Basal-like BRCA.

We further combine information of 12 established proteomics pathways[2] to transform connections between protein pairs into an integrated functional analysis where we group functional proteins belonging to the same pathway into the GraphR algorithm. In order to gain insight into pathway-level connectivities within or across each subtype, we use the connectivity score (CS), which is defined as the ratio of the connected edges to the total number of possible edges. Higher CS corresponds to denser connections in the pathway. The dot sizes of the bubble plot in Figure 4D are proportional to the CS, and clustering on the CS is conducted to decide the order of pathways in Figure 4D. Generally, CS for Luminal A and B tend to be the largest among the three groups and smallest for Her2-enriched BRCA, except for the PI3K/AKT pathway, TSC/mTOR pathway, hormone receptor pathway, and hormone signaling pathway. With respect to the hormone receptor pathway, CS for Basal-like BRCA is highest (CS = 1) and is equal to 0.67 and 0.33 in Luminal A and B and Her2-enriched cancer, partly due to the reason that all hormone receptors are likely to have a down-expression in Basal-like BRCA while the associations among hormone receptors are more complicated and diverse in the other two groups. Compared with Basal-like BRCA, Luminal A and B and Her2-enriched cancer had a slightly higher CS in terms of the TSC/mTOR pathway (Basal-like: 0.3; Luminal A and B: 0.6; Her2-enriched: 0.4).

CellPress
OPEN ACCESS

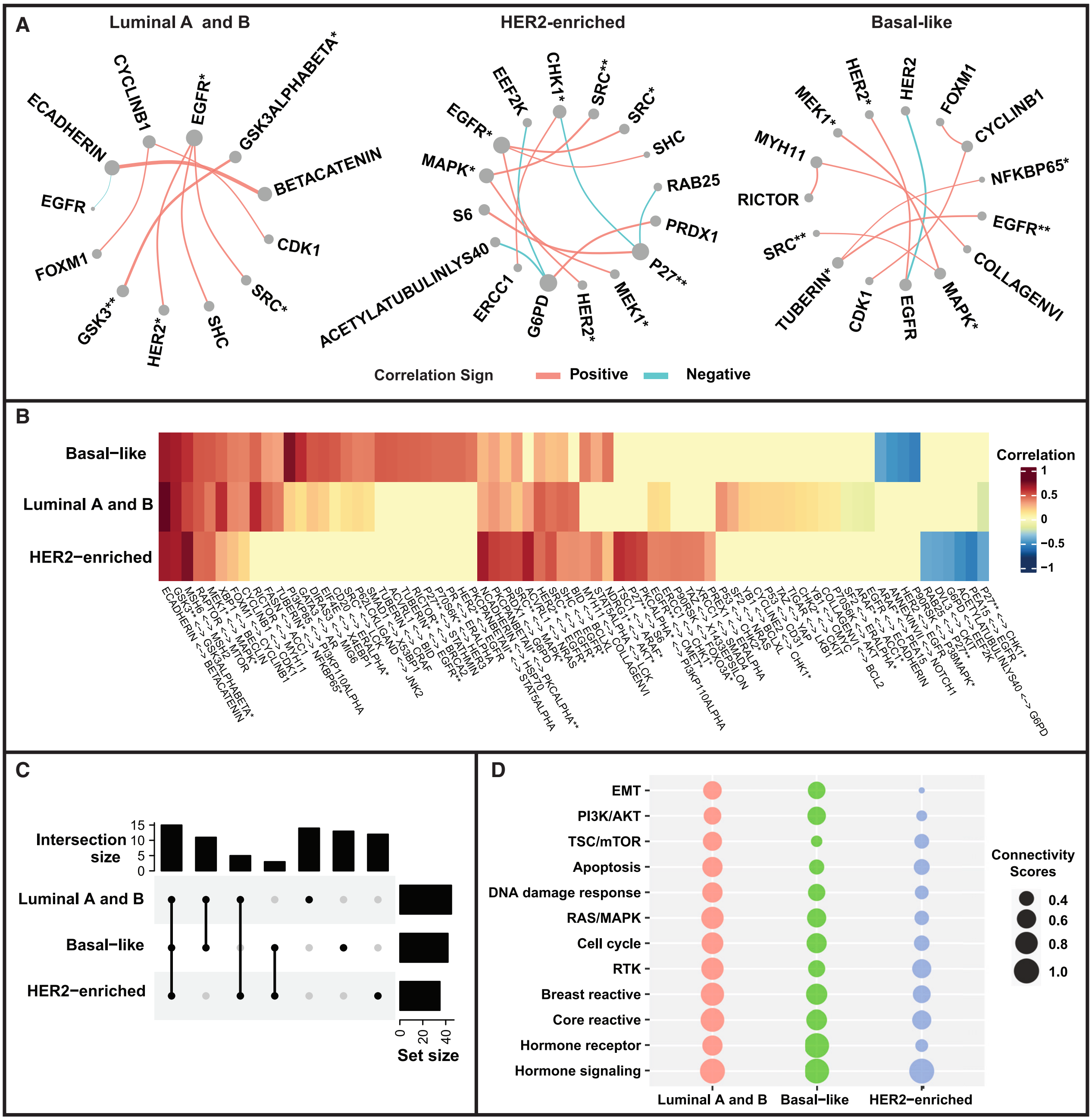

Figure 4. Proteomic network-based characterization of intrinsic subtypes of breast cancer
(A) PAM50 subtype-specific networks for proteins with top five connectivity degrees.
(B) Heatmap of partial correlations between protein pairs for each subtype of breast cancer. We include protein pairs with significantly strong partial correlations (FDR-based p values $<$ 0.01 and $|$partial correlation$| \geq 0.4$) for at least one subtype.
(C) Upset plot for sizes of significant connections within or across BRCA subtypes based on the protein pairs included in (B).
(D) Connectivity scores (CSs) of pathways for each breast cancer subtype. Pathways are ordered based on the clustering of CSs.

Pan-cancer analyses across gynecological cancers

Additionally, we have conducted pan-cancer analyses and have used GraphR to analyze proteomic data from BRCA along with three other gynecological cancers, namely cervical squamous cell carcinoma and endocervical adenocarcinoma (CESC), ovarian serous cystadenocarcinoma (OV), and uterine corpus endometrial carcinoma (UCEC). Our aim is to identify both shared and cancer-type-specific proteomic networks across gynecological cancers. We identify several hub proteins across cancer types, e.g., EGFR* in BRCA and UCEC. EGFR*, which

has been identified as hub protein in Luminal A, B, and Her2-enriched breast cancers, again plays a key role in BRCA and UCEC with the positive partial correlation with Her2* and Src*. Additional results are shown in Figure S4. Furthermore, we have applied jointGHS to infer subtype-specific proteomic networks of intrinsic subtypes of BRCA and cancer-type-specific proteomic networks across gynecological cancers.

Characterization of stemness-induced heterogeneity on proteomics networks in breast cancer

Our aim is to investigate changes in proteomic network patterns across various dedifferentiation levels of BRCA. Tumor cells exhibit dedifferentiation, a reversed growing process by which cells lose the specialized phenotype and gain properties of stem cells.[52] It is commonly accepted that the degrees of differentiation of tumor cells are significantly related to tumor behavior and thus have been widely used in the grading system for BRCA, which provides guidance for treatment and outcomes.[72] To this end, two independent stemness indices are obtained based on DNA methylation (mDNAsi) and mRNA expression (mRNAsi), which provide the degrees of dedifferentiation on epigenetic and gene expression levels, respectively, across BRCA tumors.[52] The mDNAsi and mRNAsi values range from 0 to 1 with lower values implying a tendency toward normal-like cells. We apply GraphR using the same proteomics dataset as in the previous example along with two stemness indices across 616 BRCA patients' data from TCGA,[90] treating mRNAsi, mDNAsi, and patients' ages (as a potential confounder) as three continuous intrinsic factors. Details of the preprocessing procedure can be found in STAR Methods.

We estimate proteomic networks across varying levels of mRNAsi, mDNAsi, and age. Figure 5A shows the networks for proteins with high degrees of connectivity across different quartiles of mRNAsi while setting mDNAsi and age at their median. The network connections are included based on the following three criteria[1]: protein pairs with different isoforms[2]; significant partial correlation (FDR-based p values $\langle$ 0.01) in more than half of the cases[3]; $|$ partial correlation $| \geq 0.45$ for at least one case. The median value of partial correlation between significant protein pairs are obtained for each quartile of mRNAsi. We identify shared hub proteins across all quartiles of mRNAsi such as ERCC1, and the connectivity degree for ERCC1 in the first to fourth quartiles are 1.37, 0.37, 0.43, and 1.33. In each quartile, there are also some specific hub proteins, such as P27* in the first and fourth quartiles and RAB11 in the second and third quartiles. Figure 5B shows the heatmap of partial correlations between specific protein pairs with varying mRNAsi. We observe distinct clusters of positive and negative associations on the right and left side of the heatmap respectively. In Figure 5C, we provide the line plots for five selected protein pairs to demonstrate their co-expression patterns with change in mRNAsi, e.g., partial correlations between GAPDH-FOXM1 tend to have a positive association with dedifferentiated tumor cells (high mRNAsi levels).

Finally, we conduct a proteomics pathway integrated analysis to show various patterns of CSs along with mRNAsi in Figure 5D, reflecting the heterogeneity of BRCA with the change of dedifferentiation levels. Three pathways—TSC/mTOR pathway, hormone receptor pathway, and hormone signaling pathway—are found to be insensitive to the changes of mRNAsi, maintaining a constant CS of one. We also find several pathways varying with mRNAsi, such as breast reactive pathway (CS = 0.87 when mRNAsi is around 0.21–0.5; otherwise CS = 0.93) and core reactive pathway (CS = 0.8 when mRNAsi is around 0.33–0.34; otherwise CS = 0.7).

GraphR enables network estimation incorporating spatial heterogeneity in the tumor microenvironment

We demonstrate the usage of GraphR in the estimation of gene expression networks incorporating spatial heterogeneity. We use a spatial transcriptomics dataset in BRCA, characterized by simultaneous measurements of gene expression and spatial coordinates that serve as intrinsic factors in the model. Our underlying hypothesis is that nearby spots/cells will share "closer" connectivity than farther ones. We utilize a human breast cancer dataset collected from a BRCA biopsy at a thickness of 16 μm.[79] Based on the hematoxylin and eosin staining image (Figure 6A top), we classify locations into three spatial regions: tumor, intermediate, and normal with the sizes 114, 67, and 69 spots, respectively (Figure 6A bottom). GraphR is implemented to the data that contain measurement of 100 spatially expressed genes at 250 spot locations to obtain spatially dependent networks (preprocessing steps are deferred to STAR Methods). All the results are explained with respect to the intermediate region, which serves as the baseline.

Figure 6B shows the networks of genes with the top five weighted connectivity degrees, defined as weighted mean of significant correlations (see STAR Methods) within each spatial region. Based on the networks, we find several hub genes shared across spatial regions, e.g., HLA-B (weighted connectivity degrees: 0.94 for tumor; 0.23 for intermediate; 1.35 for normal), which shows negative partial correlation with GBP-1 in the normal region and positive in the tumor region. In addition to the shared genes, we also find several genes specific to each region, e.g., COL12A1 in normal and tumor regions (weighted connectivity degrees: 0.54 for tumor; 0.76 for normal).

We present the spatially varying partial correlations of selected gene pairs in Figure 6C, demonstrating the ability of GraphR to incorporate spatial information and explicitly estimate spatially varying dependence structures. We observe negative partial correlations in the normal region and positive partial correlations in the tumor region for gene pairs THBS2-COL12A1 and XBP1-SPINT2 and the inverse partial correlation pattern for ERGIC3-FN1. We further show the overall connectivity degree patterns for selected genes via circular heatmap (Figure 6D left). Genes such as HLA-B, TGM2 and GBP1 tend to exhibit high connectivity degrees across most tumor and normal regions. Meanwhile, there are no clear patterns for some genes within each cluster, such as CLEC3A and H2AFJ, partly reflecting the spatial heterogeneity of cancers. The right panel of Figure 6D displays the spatial distribution of connectivity degrees with respect to COL12A1 (hub genes in normal and tumor regions) and HLA-B (hub genes in all regions). Networks containing all included proteins for each region and the spatial pattern of partial correlations and connectivity degrees for more gene pairs are shown in Figure S5. We have also implemented an adaptive GLASSO-based

CellPress
OPEN ACCESS

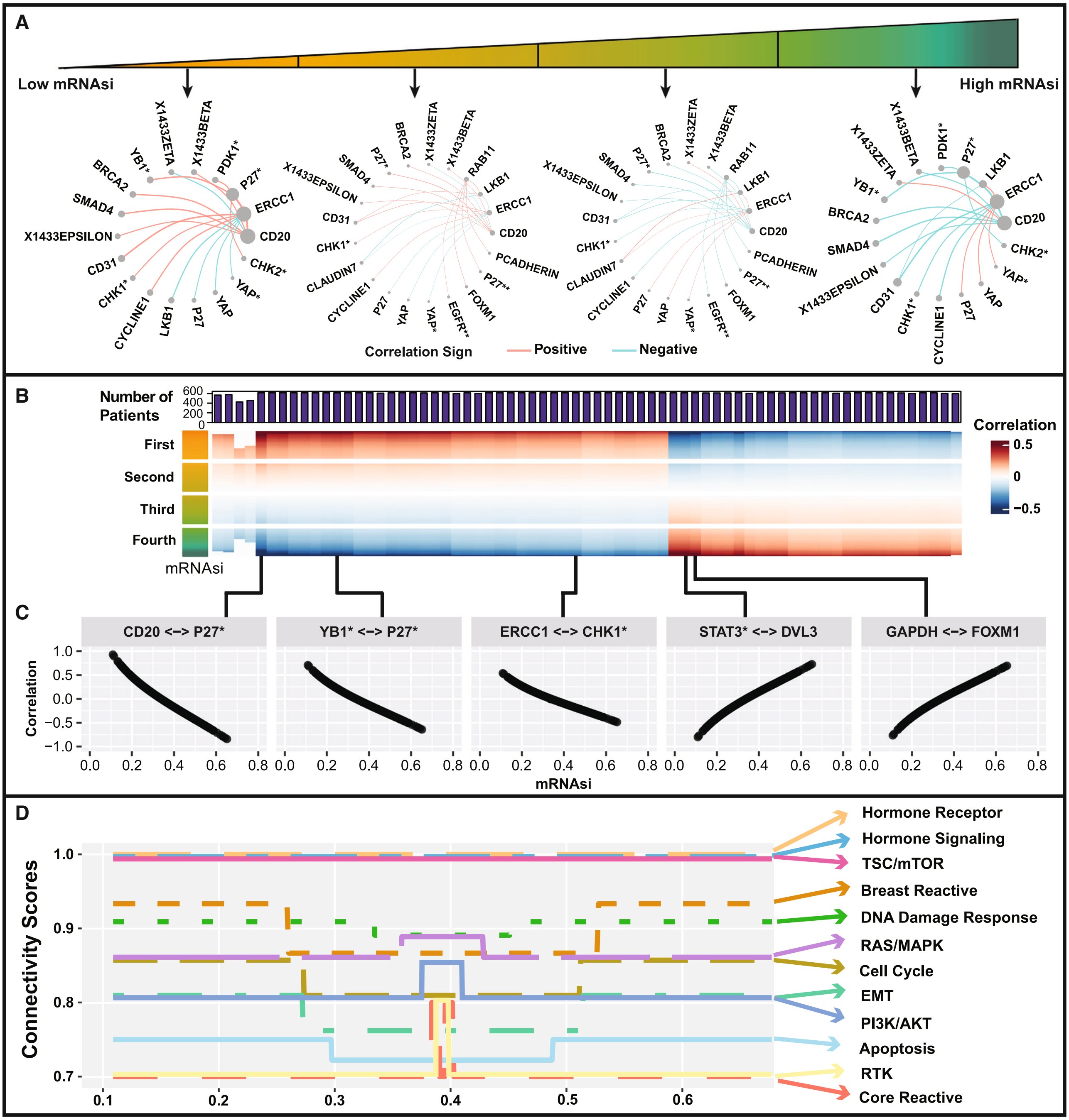

Figure 5. Characterization of stemness-induced heterogeneity on proteomics networks in breast cancer

We apply GraphR with three intrinsic factors including age, logit-transformed mRNA, and DNA methylation stemness indices (mRNAsi and mDNAsi, respectively). We only include protein pairs with significant strong partial correlation (FDR-based p values < 0.01) for more than 50% of cases and $|$partial correlation$| \geq 0.45$ for at least one case. The results are presented with varying mRNAsi, while age and mDNAsi are fixed at median.

(A) Networks for proteins with top five connectivity degrees corresponding to each quartile of mRNAsi.

(B) Heatmap of partial correlations between protein pairs with varying mRNAsi. The color bar on the left shows quarterly divided mRNAsi. The barplot on top represents the number of significant cases for the corresponding protein pair.

(C) Line plots showing association between partial correlation of selective protein pairs and mRNAsi of the original scale.

(D) CSs of pathways changing along mRNAsi. CS is calculated as the ratio of the observed number of edges between proteins belonging to the corresponding pathway to the total number of possible edges.

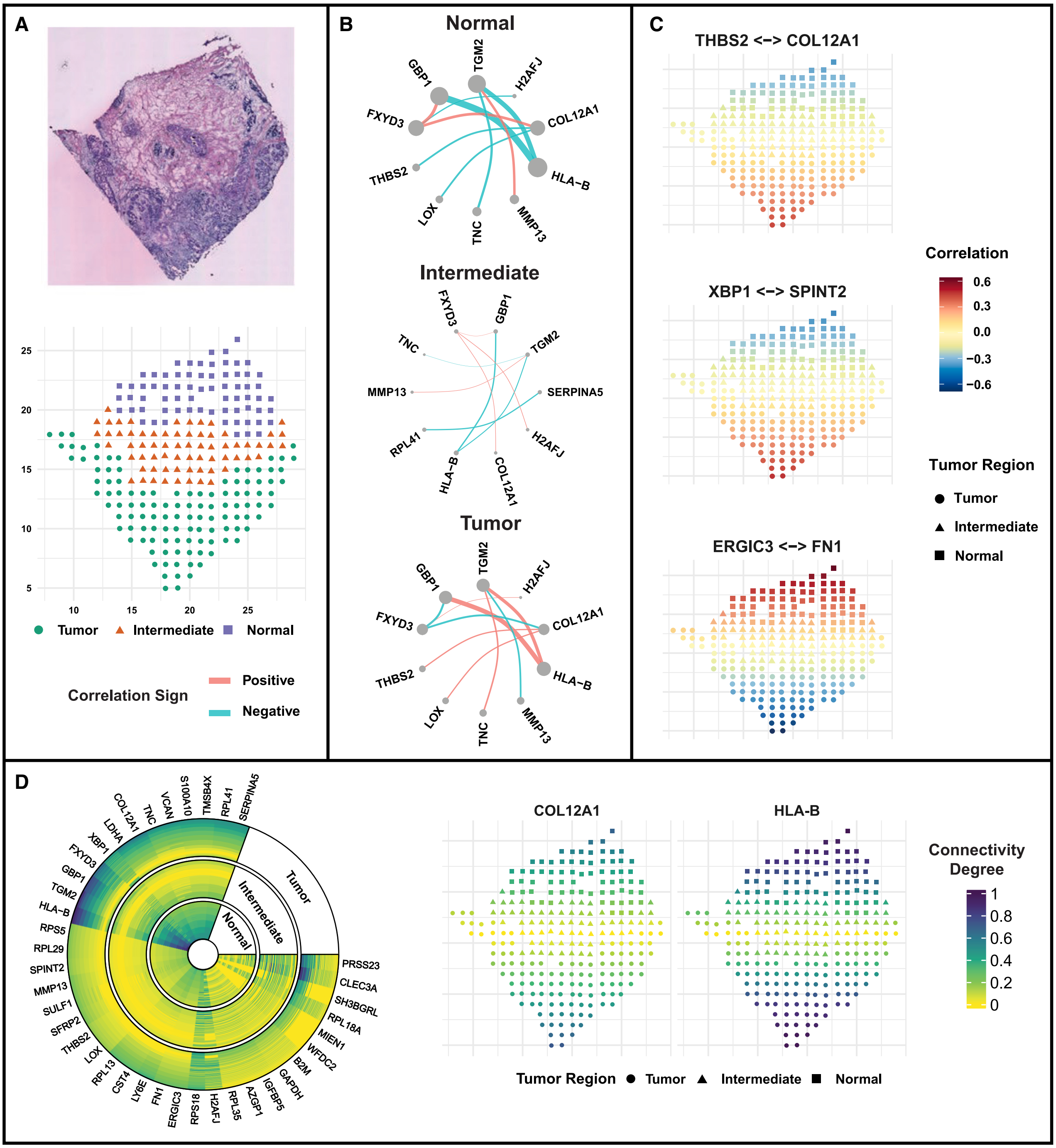

Figure 6. Gene expression network estimation incorporating spatial heterogeneity

(A) The hematoxylin and eosin-stained image (top) and the spatial distribution of measured spots (bottom) that are classified into tumor, intermediate, and normal regions.[79]

(B) The networks of genes with top five weighted connectivity degrees on the right with respect to each spatial region. Weighted connections of each spatial region are defined as the weighted mean of significant partial correlations (FDR-based p values > 0.01) for all spots in the corresponding region with weights being posterior inclusion probability of these connections. Width of edges represent weighted partial correlations and sizes of nodes represent weighted connectivity degrees.

(C) Spatial pattern of partial correlations for selective gene pairs (THBS2-COL12A1; XBP1-SPINT2; ERGIC3-FN1) where point shapes defer for each spatial region.

(legend continued on next page)

approach for comparative study of findings from spatial transcriptomics data (see Figure S5).

Computation cost

A major advantage of GraphR, over existing Bayesian approaches that allow for the estimation of sample-specific graphs,[64,89] is its computational efficiency. Instead of applying MCMC, one of the gold standards for posterior inference, we apply variational Bayes algorithm (Method S1), which significantly improves the scalability with high-dimensional settings without sacrificing accuracy. For example, in multi-category graph cases where $n = 100$, $p = 20$, $q = 2$ and sparsity level is at 5%, GraphR is significantly faster, completing computations in 0.373 s while Bayesian Edge Regression takes an average runtime of 1295.29 s. This makes GraphR approximately 3,500 times faster than Bayesian Edge Regression. In case of continuously varying graphs setting where $n = 100$, $p = 20$, $q = 1$ and 5% level of sparsity, GraphR is significantly faster, taking only 0.21 s to complete, which makes it approximately 5,168 times faster than GGMx, which takes 1085.29 s. This promising feature positions GraphR for routine use in biomedical research, especially when dealing with high-throughput multi-omics data.

In terms of computational complexity, the complexity of GraphR is $O(p^2qN)$, where p is the number of nodes, q is the number of intrinsic factors, and N is the sample size—hence scales quadratically with the number of features and linearly with intrinsic factors (covariates) and sample size. Figure S2F presents the relationship between mean computation time (log-transformed) and the number of edges to be detected (log-transformed) for various n/pq ratios, which shows it is roughly linear in the log-scale for the various ratios. For the real datasets the computational times are 2.62 min, 4.23 min, and 17.33 s for the intrinsic subtypes of the BRCA dataset, the stemness indices of the BRCA dataset, and the spatial transcriptomics dataset, respectively.

GraphR package and Shiny Application is a comprehensive and scalable tool for heterogeneous network estimation and visualization

GraphR is implemented in a fast and user-friendly R-package that implements a variety of functions for network estimation, prediction, and network visualizations. GraphR is a modular package that can incorporate different types and scales of intrinsic factors along with continuous feature matrix and can estimate heterogeneous networks that can be nimbly used for downstream interpretations and visualizations. We have also developed a stand-alone *GraphR* Shiny App that provides users with a dynamic platform for exploring, visualizing, and reproducing the heterogeneous networks using all the datasets in this paper. The Shiny App provides point-and-click visualizations and explorations of all the discrete (cancer subtype specific networks; Figure 4A), continuous (stemness indices; Figure 5A), and spatially varying networks (Figure 6). In addition, it provides users a seamless platform for implementing GraphR to their own datasets, all without the need for prior knowledge of R-coding.

DISCUSSION

Sample-specific heterogeneity exists across many disease contexts, with cancer being a prime example, wherein tumor heterogeneity manifests at inter- and intra-patient levels. In this article, we present GraphR, a supervised probabilistic network modeling approach for estimating sample-specific heterogeneous networks as opposed to standard graphical modeling methods that often assume homogeneity among samples and thus provide an "average" view of the entire cohort, which might lead to erroneous conclusions through confounding, as illustrated via the Simpson's paradox (Figure 2). To this end, GraphR enables seamless integration with various intrinsic factors of heterogeneity such as group-specific, sample-specific, or spatial heterogeneity—thus providing more precise and robust estimation of graph structures. Furthermore, GraphR employs sparsity-inducing priors facilitating efficient estimation with limited sample sizes along with providing uncertainty quantification (controls multiplicity) on the selection of both edges and intrinsic factors of heterogeneity. As we show through multiple simulation settings, this achieves the right balance between sensitivity and specificity—high accuracy in graph estimation along with low false positive rates—compared with multiple other competing methods.

GraphR offers several advantages. First, it provides greater flexibility through graphical regression approaches. Methods for multi-category graph inference are effective when samples can be divided into homogeneous subgroups. However, they are limited in their abilities to handle continuous variables, such as spatially varying structure or continuous measurements that do not fit into predefined categories. In contrast, GraphR not only demonstrates strong performance in multi-category graph inference (Figure 3) but is also well-suited for scenarios where variables are continuous, including spatial and other continuous measures, which makes it adaptable to a wider range of data settings. This flexibility allows for a more detailed exploration of the relationships among variables, regardless of whether they fall into discrete subgroups or vary continuously. Additionally, GraphR benefits from computational efficiency through its use of MFVB. MFVB offers computational efficiency and scalability for high-dimensional graphical model inference, achieving a balance between accuracy and speed. While fully Bayesian approaches provide precise estimations through exact inference, our empirical results demonstrate that variational Bayes (VB) significantly enhances the practical applicability of GraphR in real-world scenarios.

GraphR can enable a more systematic and unbiased understanding of the underlying biological mechanisms and their changes incorporating inter- and intra-tumor heterogeneity. In the context of subtype-specific proteomic networks, we observe

(D) Circular heatmap of connectivity degrees of genes for each cluster (left) and spatial pattern of connectivity degrees of selected genes COL12A1 and XBP1 (right). Connectivity degree is defined as the sum of magnitudes of significant edges (i.e., absolute values of partial correlations) between the corresponding node and others. Here, the connectivity degree is rescaled to a maximum of 1 by dividing by the maximum value.

a high correlation between phosphorylated EGFR and Her2 in Her2-enriched and a subset of Luminal A and B BRCA, consistent with previous findings,[39] where a strong correlation between phosphorylated EGFR and Her2 was observed and confirmed by multiple methods in most Her2-enriched and some Luminal A and B BRCA cases. Furthermore, Shc has been identified as a key factor in the EGFR downstream signaling pathway, with the interaction between Shc and EGFR being linked to MAPK pathway activation.[47,71] Additionally, the synergism between Src and EGFR is associated with accelerated oncogenesis, resulting in increased Shc levels.[12,56] Moreover, we also find that E-cadherin and β-catenin are positively correlated in all subtypes of BRCA. E-cadherin is regarded as one of the most important features in epithelial cells and it can combine with catenins to form a functional complex for the intercellular adhesion.[10,91] In terms of integrated functional analysis, Luminal A and B and Her2-enriched cancer exhibits a slightly higher CS for the TSC/mTOR pathway in comparison of Basal-like BRCA, aligning with the role of Her2 expression in activating the PI3K-mTOR signaling in BRCA.[33]

For the application of dedifferentiation-related proteomic networks, we find that partial correlations between GAPDH-FOXM1 tend to have a positive association with dedifferentiated tumor cells (high mRNAsi levels). FOXM1 is a pro-stemness transcription factor that is suggested to have a driving effect on cell dedifferentiation and proliferation in BRCA.[52] At the same time, GAPDH is also identified as a key factor with respect to proliferation and aggressiveness of breast cancer cells, which has a significant positive correlation with higher histoprognostic grades.[31] Our results demonstrate a positively increasing trend of correlation between GAPDH and FOXM1 for BRCA along with mRNAsi, which are consistent with previous findings and also suggest a potential interaction between two proteins in the cell dedifferentiation and proliferation process.

Additionally, the proteomic connectivity of the RAS/MAPK pathway is found to be relatively high in Luminal A and B and Basal-like BRCA. It has been shown that the RAS/MAPK pathway is closely associated with BRCA, and several Food and Drug Administration-approved prescription drugs that regulate or inhibit the MAPK pathway including Farnesyltransferase inhibitors and Sorafenib may reduce metastatic progression in BRCA,[38] especially for Basal-like BRCA.[30,51] Furthermore, the RAS/MAPK pathway is also shown as a critical signaling pathway involved in various cellular processes, including cell differentiation, proliferation, and apoptosis,[53] which partly explains the changing pattern of CS along with the stemness indices and thereby implies potential variations in behaviors among BRCA characterized by varying levels of differentiation. Beyond BRCA, we also find the RAS/MAPK pathway has relatively high CSs in UCEC and OV. In the case of UCEC, insulin has been linked to tumor development through the PI3K/Akt and RAS/MAPK pathways,[59] which further elaborates the potential anticancer effects of metformin, a common treatment for type 2 diabetes, through the RAS/MAPK pathway.[36,85] Furthermore, the elevated CS observed in OV aligns with previous research suggesting that inhibitors of the RAS/MAPK may improve patient survival.[35]

In the context of the tumor microenvironment, GraphR can be used to infer spatially varying networks and subsequently identify hub genes and connections that are specific to regions or cell types when applied to spatial transcriptomic data. In the spatial transcriptomic application, we observe negative partial correlations in the normal region and positive partial correlations in the tumor region for the gene pairs THBS2-COL12A1 and XBP1-SPINT2. COL12A1 and THBS2 are components of extracellular matrix (ECM) proteins that significantly upregulate in breast cancer compared with normal breast tissue.[83] Furthermore, the spatial pattern between XBP1 and SPINT2 aligns with previous findings,[1] which identify SPINT2 as a hub gene for tumor and intermediate regions, while XBP1 is a hub gene in the tumor region only. XBP1 induces cell invasion and metastasis in breast cancer cells, with high expression promoting tumor cell proliferation.[16] Additionally, we have found HLA-B as a hub gene across all regions and is negatively correlated with GBP1 in the normal region and positive partial correlation in the tumor region. HLA-B, a molecule in the MHC class I family, plays a critical role in the cell-mediated immune response by presenting intracellular proteins or neoantigens produced by cancer cells to effector $CD8^+$ T cells, thereby initiating an immune response.[66,77] It has been shown to be associated with favorable outcome in breast cancers through increased immune T cell activation.[18,66] Moreover, GBP-1, has also been identified as a regulator of T cell activation.[24,74] GBP-1 displays relatively strong expression in infiltrating cells while exhibiting weak expression in tumor cells,[5] which elucidates the spatial interaction pattern between HLA-B and GBP-1.

One challenge in the graphical model estimation is ensuring the positive definiteness (PD) of the estimated precision matrices. While GraphR guarantees symmetry in undirected settings, it does not inherently ensure PD. Achieving PD is particularly challenging in high-dimensional settings with multiple covariate combinations (discrete, continuous, and spatial) and often requires computationally intensive algorithms. Most graphical regression methods, such as those by Wang et al. (2021) and Zhang et al. (2022),[89,94] do not explicitly incorporate this constraint, with the exception of GGMx,[64] which ensures PD but struggles with high-dimensional scaling. GraphR focuses on edge selection and, as shown in our simulations (Figure 3), accurately captures graph structures despite not enforcing the PD constraint, achieving high performance metrics, including an MCC of 0.892 and 0.955 in multi-category and continuously varying graph estimation. This suggests that the absence of a PD constraint does not significantly impact edge selection accuracy.

Limitations of the study

The GraphR method, while robust, has certain limitations that could benefit from further generalizations. First, GraphR primarily relies on linear assumptions between the partial correlations and intrinsic heterogeneity factors, which although interpretable and efficient to compute, can be generalized to capture non-linear associations. An effective extension to overcome this limitation could involve the incorporation of Gaussian processes or other non-parametric models, which will allow for a more flexible modeling of network structures, but at an added

computational cost. Second, GraphR assumes multivariate normally distributed data, which may not hold for certain 'omics data types such as mutation data. To address this, an extension to a mixed graphical model could be considered,[11] allowing the method to handle a wider variety of data types and distributional characteristics. Finally, GraphR only considers complete cases, which means that any samples with missing data are excluded from the analysis. Missing data strategies can be potentially employed as a precursor to fitting GraphR and the methodological framework of GraphR can also be extended to incorporate missing data imputation. We leave these tasks for future explorations.

RESOURCES AVAILABILITY

Lead contact

Further information and requests for resources and code should be directed to and will be fulfilled by the lead contact, Prof. Veera Baladandayuthapani (veerab@umich.edu).

Materials availability

This study did not generate new unique reagents.

Data and code availability

- This paper analyzes existing, publicly available data. These accession numbers for the datasets are listed in the key resources table.
- All codes have been deposited at Zenodo (https://zenodo.org/records/11287580). GraphR's open-source R-package is publicly available on Github (github.com/bayesrx/GraphR). The Shiny App is available on bayesrx.shinyapps.io/GraphR/.
- Any additional information required to reanalyze the data reported in this paper is available from the lead contact upon request.

ACKNOWLEDGMENTS

We like to thank Mike Kleinsasser for valuable guidance in Shiny App development.

V.B. was supported by NIH grants R01-CA160736, R01CA244845-01A1, and P30 CA46592; NSF grant 1463233; and start-up funds from the U-M Rogel Cancer Center and School of Public Health. S.A. was supported by the Postdoctoral Fellows Grant of the University of Michigan Rogel Cancer Center, which helped us to host the Shiny App server. Y.N. was supported by NIH grant 1R01GM148974-01 and NSF grant DMS-2112943.

AUTHOR CONTRIBUTIONS

V.B. conceived the idea and provided funding support. L.C., S.A., and V.B. designed the experiments. L.C. and S.A. developed the method, implemented the software, performed simulations, and analyzed real data. C.L. developed the accompanying Shiny App. L.C., S.A., Y.N., and V.B. wrote the manuscript.

DECLARATION OF INTERESTS

The authors declare no competing interests.

DECLARATION OF GENERATIVE AI AND AI-ASSISTED TECHNOLOGIES IN THE WRITING PROCESS

During the preparation of this work, the authors used GPT-4 to enhance the writing of the manuscript. After utilizing this tool, the authors carefully reviewed and revised the content as necessary and take full responsibility for the final version of the publication.

STAR★METHODS

Detailed methods are provided in the online version of this paper and include the following:

- KEY RESOURCES TABLE
- METHOD DETAILS
  - Heterogeneous graphical regression models
  - Priors for GraphR
  - Model fitting via variational Bayes
  - Network construction
  - Simulated data
  - Simulation design for Simpon's paradox
  - Preprocessing for application
  - Network summaries
- QUANTIFICATION AND STATISTICAL ANALYSIS

SUPPLEMENTAL INFORMATION

Supplemental information can be found online at https://doi.org/10.1016/j.crmeth.2025.100984.

Received: May 24, 2024
Revised: October 28, 2024
Accepted: January 24, 2025
Published: February 14, 2025

REFERENCES

1. Acharyya, S., Zhou, X., and Baladandayuthapani, V. (2022). SpaceX: gene co-expression network estimation for spatial transcriptomics. Bioinformatics *38*, 5033–5041.
2. Akbani, R., Ng, P.K.S., Werner, H.M.J., Shahmoradgoli, M., Zhang, F., Ju, Z., Liu, W., Yang, J.-Y., Yoshihara, K., Li, J., et al. (2014). A pan-cancer proteomic perspective on The Cancer Genome Atlas. Nat. Commun. *5*, 3887.
3. Alizadeh, A.A., Aranda, V., Bardelli, A., Blanpain, C., Bock, C., Borowski, C., Caldas, C., Califano, A., Doherty, M., Elsner, M., et al. (2015). Toward understanding and exploiting tumor heterogeneity. Nat. Med. *21*, 846–853.
4. Altenbuchinger, M., Weihs, A., Quackenbush, J., Grabe, H.J., and Zacharias, H.U. (2020). Gaussian and Mixed Graphical Models as (multi-)omics data analysis tools. Biochim. Biophys. Acta. Gene Regul. Mech. *1863*, 194418. Transcriptional Profiles and Regulatory Gene Networks.
5. Ascierto, M.L., Kmieciak, M., Idowu, M.O., Manjili, R., Zhao, Y., Grimes, M., Dumur, C., Wang, E., Ramakrishnan, V., Wang, X.-Y., et al. (2012). A signature of immune function genes associated with recurrence-free survival in breast cancer patients. Breast Cancer Res. Treat. *131*, 871–880.
6. Attias, H., Platt, J., Acero, A., and Deng, L. (2000). Speech denoising and dereverberation using probabilistic models. Adv. Neural. Inf. Process. Syst. *13*.
7. Baladandayuthapani, V., Ji, Y., Talluri, R., Nieto-Barajas, L.E., and Morris, J.S. (2010). Bayesian random segmentation models to identify shared copy number aberrations for array cgh data. J. Am. Stat. Assoc. *105*, 1358–1375.
8. Barabási, A.-L. (2013). Network science. Phil. Trans. R. Soc. A. *371*, 20120375.

9. Barabási, A.-L., and Oltvai, Z.N. (2004). Network biology: understanding the cell's functional organization. Nat. Rev. Genet. *5*, 101–113.

10. Beavon, I.R. (2000). The e-cadherin–catenin complex in tumour metastasis: structure, function and regulation. Eur. J. Cancer *36*, 1607–1620.

11. Bhadra, A., Rao, A., and Baladandayuthapani, V. (2018). Inferring network structure in non-normal and mixed discrete-continuous genomic data. Biometrics *74*, 185–195.

12. Biscardi, J.S., Ishizawar, R.C., Silva, C.M., and Parsons, S.J. (2000). Tyrosine kinase signalling in breast cancer: epidermal growth factor receptor and c-Src interactions in breast cancer. Breast Cancer Res. *2*, 203.

13. Bishop, C.M., and Nasrabadi, N.M. (2006). Pattern Recognition and Machine Learning, *volume 4* (Springer).

14. Blyth, C.R. (1972). On simpson's paradox and the sure-thing principle. J. Am. Stat. Assoc. *67*, 364–366.

15. Castillo, I., and van der Vaart, A. (2012). Needles and straw in a haystack: Posterior concentration for possibly sparse sequences. Ann. Stat. *40*, 2069–2101.

16. Chen, S., Chen, J., Hua, X., Sun, Y., Cui, R., Sha, J., and Zhu, X. (2020). The emerging role of XBP1 in cancer. Biomed. Pharmacother. *127*, 110069.

17. Colaprico, A., Silva, T.C., Olsen, C., Garofano, L., Cava, C., Garolini, D., Sabedot, T.S., Malta, T.M., Pagnotta, S.M., Castiglioni, I., et al. (2016). Tcgabiolinks: an r/bioconductor package for integrative analysis of tcga data. Nucleic Acids Res. *44*, e71.

18. Concha, A., Esteban, F., Cabrera, T., Ruiz-Cabello, F., and Garrido, F. (1991). Tumor aggressiveness and MHC class I and II antigens in laryngeal and breast cancer. Semin. Cancer Biol. *2*, 47–54.

19. Dai, X., Li, T., Bai, Z., Yang, Y., Liu, X., Zhan, J., and Shi, B. (2015). Breast cancer intrinsic subtype classification, clinical use and future trends. Am. J. Cancer Res. *5*, 2929–2943.

20. Danaher, P., Wang, P., and Witten, D.M. (2014). The Joint Graphical Lasso for Inverse Covariance Estimation Across Multiple Classes. J. R. Stat. Soc. Series B Stat. Methodol. *76*, 373–397.

21. Dempster, A.P. (1972). Covariance Selection. Biometrics *28*, 157–175.

22. Dobra, A., Hans, C., Jones, B., Nevins, J.R., Yao, G., and West, M. (2004). Sparse graphical models for exploring gene expression data. J. Multivariate Anal. *90*, 196–212. Special Issue on Multivariate Methods in Genomic Data Analysis.

23. Eppert, K., Takenaka, K., Lechman, E.R., Waldron, L., Nilsson, B., van Galen, P., Metzeler, K.H., Poeppl, A., Ling, V., Beyene, J., et al. (2011). Stem cell gene expression programs influence clinical outcome in human leukemia. Nat. Med. *17*, 1086–1093.

24. Forster, F., Paster, W., Supper, V., Schatzlmaier, P., Sunzenauer, S., Ostler, N., Saliba, A., Eckerstorfer, P., Britzen-Laurent, N., Schütz, G., et al. (2014). Guanylate binding protein 1–mediated interaction of T cell antigen receptor signaling with the cytoskeleton. J. Immunol. *192*, 771–781.

25. Friedman, J., Hastie, T., and Tibshirani, R. (2008). Sparse inverse covariance estimation with the graphical lasso. Biostatistics *9*, 432–441.

26. Friedman, N. (2004). Inferring Cellular Networks Using Probabilistic Graphical Models. Science *303*, 799–805.

27. Gan, L., Narisetty, N.N., and Liang, F. (2019). Bayesian regularization for graphical models with unequal shrinkage. J. Am. Stat. Assoc. *114*, 1218–1231.

28. Gelfand, A.E., and Smith, A.F.M. (1990). Sampling-based approaches to calculating marginal densities. J. Am. Stat. Assoc. *85*, 398–409.

29. George, E.I., and McCulloch, R.E. (1993). Variable selection via Gibbs sampling. J. Am. Stat. Assoc. *88*, 881–889.

30. Giltnane, J.M., and Balko, J.M. (2014). Rationale for targeting the Ras/MAPK pathway in triple-negative breast cancer. Discov. Med. *17*, 275–283.

31. Guo, C., Liu, S., and Sun, M.-Z. (2013). Novel insight into the role of gapdh playing in tumor. Clin. Transl. Oncol. *15*, 167–172.

32. Ha, M.J., Banerjee, S., Akbani, R., Liang, H., Mills, G.B., Do, K.-A., and Baladandayuthapani, V. (2018). Personalized Integrated Network Modeling of the Cancer Proteome Atlas. Sci. Rep. *8*, 14924.

33. Hare, S.H., and Harvey, A.J. (2017). mtor function and therapeutic targeting in breast cancer. Am. J. Cancer Res. *7*, 383–404.

34. Hastings, W.K. (1970). Monte Carlo Sampling Methods Using Markov Chains and Their Applications. Biometrika *57*, 97–109.

35. Hollis, R.L., Thomson, J.P., Stanley, B., Churchman, M., Meynert, A.M., Rye, T., Bartos, C., Iida, Y., Croy, I., Mackean, M., et al. (2020). Molecular stratification of endometrioid ovarian carcinoma predicts clinical outcome. Nat. Commun. *11*, 4995.

36. Iglesias, D.A., Yates, M.S., Van Der Hoeven, D., Rodkey, T.L., Zhang, Q., Co, N.N., Burzawa, J., Chigurupati, S., Celestino, J., Bowser, J., et al. (2013). Another surprise from metformin: novel mechanism of action via K-Ras influences endometrial cancer response to therapy. Mol. Cancer Therapeut. *12*, 2847–2856.

37. Joung, J.-G., and Fei, Z. (2009). Identification of microRNA regulatory modules in Arabidopsis via a probabilistic graphical model. Bioinformatics *25*, 387–393.

38. Khojasteh Poor, F., Keivan, M., Ramazii, M., Ghaedrahmati, F., Anbiyaiee, A., Panahandeh, S., Khoshnam, S.E., and Farzaneh, M. (2021). Mini review: The FDA-approved prescription drugs that target the MAPK signaling pathway in women with breast cancer. Breast Dis. *40*, 51–62.

39. Cancer Genome Atlas Network; Koboldt, D.C., Fulton, R.S., McLellan, M.D., Schmidt, H., Kalicki-Veizer, J., and McMichael, J.F. (2012). Comprehensive molecular portraits of human breast tumours. Nature *490*, 61–70.

40. Koller, D., and Friedman, N. (2009). Probabilistic Graphical Models: Principles and Techniques (MIT press).

41. Kuijjer, M.L., Tung, M.G., Yuan, G., Quackenbush, J., and Glass, K. (2019). Estimating Sample-Specific Regulatory Networks. iScience *14*, 226–240.

42. Lauritzen, S.L. (1996). Graphical Models, *volume 17* (Clarendon Press).

43. Lee, W.-C., Diao, L., Wang, J., Zhang, J., Roarty, E.B., Varghese, S., Chow, C.-W., Fujimoto, J., Behrens, C., Cascone, T., et al. (2018). Multiregion gene expression profiling reveals heterogeneity in molecular subtypes and immunotherapy response signatures in lung cancer. Mod. Pathol. *31*, 947–955.

44. Li, B., Chuns, H., and Zhao, H. (2012). Sparse Estimation of Conditional Graphical Models with Application to Gene Networks. J. Am. Stat. Assoc. *107*, 152–167.

45. Li, J., Lu, Y., Akbani, R., Ju, Z., Roebuck, P.L., Liu, W., Yang, J.-Y., Broom, B.M., Verhaak, R.G.W., Kane, D.W., et al. (2013). TCPA: a resource for cancer functional proteomics data. Nat. Methods *10*, 1046–1047.

46. Li, Z., Mccormick, T., and Clark, S. (2019). Bayesian joint spike-and-slab graphical lasso. In Conference on Machine Learning, K. Chaudhuri and R. Salakhutdinov, eds. (PMLR), pp. 3877–3885.

47. Lin, C.-C., Suen, K.M., Stainthorp, A., Wieteska, L., Biggs, G.S., Leitão, A., Montanari, C.A., and Ladbury, J.E. (2019). Targeting the Shc-EGFR interaction with indomethacin inhibits MAP kinase pathway signalling. Cancer Lett. *457*, 86–97.

48. Lingjærde, C., Fairfax, B.P., Richardson, S., and Ruffieux, H. (2022). Scalable multiple network inference with the joint graphical horseshoe. Ann. Appl. Stat. *18*, 1899–1923.

49. Liu, H., Chen, X., Wasserman, L., and Lafferty, J. (2010). Graph-Valued Regression. Adv. Neural Inf. Process. Syst. *23*.

50. Liu, X., Wang, Y., Ji, H., Aihara, K., and Chen, L. (2016). Personalized characterization of diseases using sample-specific networks. Nucleic Acids Res. *44*, e164.

51. Loi, S., Dushyanthen, S., Beavis, P.A., Salgado, R., Denkert, C., Savas, P., Combs, S., Rimm, D.L., Giltnane, J.M., Estrada, M.V., et al. (2016). RAS/MAPK activation is associated with reduced tumor-infiltrating lymphocytes in triple-negative breast cancer: therapeutic cooperation between MEK and PD-1/PD-L1 immune checkpoint inhibitors. Clin. Cancer Res. *22*, 1499–1509.

52. Malta, T.M., Sokolov, A., Gentles, A.J., Burzykowski, T., Poisson, L., Weinstein, J.N., Kamińska, B., Huelsken, J., Omberg, L., Gevaert, O., et al. (2018). Machine learning identifies stemness features associated with oncogenic dedifferentiation. Cellule *173*, 338–354.e15.

53. Masliah-Planchon, J., Garinet, S., and Pasmant, E. (2016). RAS-MAPK pathway epigenetic activation in cancer: miRNAs in action. Oncotarget *7*, 38892–38907.

54. Matthews, B.W. (1975). Comparison of the predicted and observed secondary structure of t4 phage lysozyme. Biochim. Biophys. Acta *405*, 442–451.

55. Meinshausen, N., and Bühlmann, P. (2006). High-dimensional graphs and variable selection with the Lasso. Ann. Stat. *34*, 1436–1462.

56. Miyake, T., and Parsons, S.J. (2012). Functional interactions between Choline kinase α, epidermal growth factor receptor and c-Src in breast cancer cell proliferation. Oncogene *31*, 1431–1441.

57. Mohammadi, A., and Wit, E.C. (2015). Bayesian Structure Learning in Sparse Gaussian Graphical Models. Bayesian Anal. *10*, 109–138.

58. Morris, J.S., Brown, P.J., Herrick, R.C., Baggerly, K.A., and Coombes, K.R. (2008). Bayesian analysis of mass spectrometry proteomic data using wavelet-based functional mixed models. Biometrics *64*, 479–489.

59. Mu, N., Zhu, Y., Wang, Y., Zhang, H., and Xue, F. (2012). Insulin resistance: a significant risk factor of endometrial cancer. Gynecol. Oncol. *125*, 751–757.

60. Narisetty, N.N., and He, X. (2014). Bayesian variable selection with shrinking and diffusing priors. Ann. Stat. *42*, 789–817.

61. Ni, Y., Baladandayuthapani, V., Vannucci, M., and Stingo, F.C. (2021). Bayesian graphical models for modern biological applications. Stat. Methods Appl. *31*, 197–225.

62. Ni, Y., Baladandayuthapani, V., Vannucci, M., and Stingo, F.C. (2022a). Bayesian graphical models for modern biological applications. Stat. Methods Appt. *31*, 197–225.

63. Ni, Y., Stingo, F.C., and Baladandayuthapani, V. (2019). Bayesian Graphical Regression. J. Am. Stat. Assoc. *114*, 184–197.

64. Ni, Y., Stingo, F.C., and Baladandayuthapani, V. (2022b). Bayesian Covariate-Dependent Gaussian Graphical Models with Varying Structure. J. Mach. Learn. Res. *23*.

65. Niemira, M., Collin, F., Szalkowska, A., Bielska, A., Chwialkowska, K., Reszec, J., Niklinski, J., Kwasniewski, M., and Kretowski, A. (2019). Molecular Signature of Subtypes of Non-Small-Cell Lung Cancer by Large-Scale Transcriptional Profiling: Identification of Key Modules and Genes by Weighted Gene Co-Expression Network Analysis (WGCNA). Cancer *12*, 37.

66. Noblejas-López, M.d.M., Nieto-Jiménez, C., Morcillo García, S., Pérez-Peña, J., Nuncia-Cantarero, M., Andrés-Pretel, F., Galán-Moya, E.M., Amir, E., Pandiella, A., Győrffy, B., and Ocana, A. (2019). Expression of MHC class I, HLA-A and HLA-B identifies immune-activated breast tumors with favorable outcome. OncoImmunology *8*, e1629780.

67. Ormerod, J.T., and Wand, M.P. (2010). Explaining variational approximations. Am. Statistician *64*, 140–153.

68. Parker, J.S., Mullins, M., Cheang, M.C.U., Leung, S., Voduc, D., Vickery, T., Davies, S., Fauron, C., He, X., Hu, Z., et al. (2009). Supervised risk predictor of breast cancer based on intrinsic subtypes. J. Clin. Oncol. *27*, 1160–1167.

69. Peng, J., Wang, P., Zhou, N., and Zhu, J. (2009). Partial Correlation Estimation by Joint Sparse Regression Models. J. Am. Stat. Assoc. *104*, 735–746.

70. Prat, A., Bianchini, G., Thomas, M., Belousov, A., Cheang, M.C.U., Koehler, A., Gómez, P., Semiglazov, V., Eiermann, W., Tjulandin, S., et al. (2014). Research-based pam50 subtype predictor identifies higher responses and improved survival outcomes in her2-positive breast cancer in the noah study. Clin. Cancer Res. *20*, 511–521.

71. Prenzel, N., Fischer, O.M., Streit, S., Hart, S., and Ullrich, A. (2001). The epidermal growth factor receptor family as a central element for cellular signal transduction and diversification. Endocr. Relat. Cancer *8*, 11–31.

72. Rakha, E.A., Reis-Filho, J.S., Baehner, F., Dabbs, D.J., Decker, T., Eusebi, V., Fox, S.B., Ichihara, S., Jacquemier, J., Lakhani, S.R., et al. (2010). Breast cancer prognostic classification in the molecular era: the role of histological grade. Breast Cancer Res. *12*, 207.

73. Saegusa, T., and Shojaie, A. (2016). Joint estimation of precision matrices in heterogeneous populations. Electron. J. Stat. *10*, 1341–1392.

74. Selleck, E.M., Fentress, S.J., Beatty, W.L., Degrandi, D., Pfeffer, K., Virgin, H.W., 4th, MacMicking, J.D., and Sibley, L.D. (2013). Guanylate-binding protein 1 (Gbp1) contributes to cell-autonomous immunity against Toxoplasma gondii. PLoS Pathog. *9*, e1003320.

75. Seoane, J., and De Mattos-Arruda, L. (2014). The challenge of intratumour heterogeneity in precision medicine. J. Intern. Med. *276*, 41–51.

76. Siegel, R.L., Miller, K.D., Wagle, N.S., and Jemal, A. (2023). Cancer statistics, 2023. CA A Cancer J. Clin. *73*, 17–48.

77. da Silva, G.B.R.F., Silva, T.G.A., Duarte, R.A., Neto, N.L., Carrara, H.H.A., Donadi, E.A., Gonçalves, M.A.G., Soares, E.G., and Soares, C.P. (2013). Expression of the classical and nonclassical hla molecules in breast cancer. Int. J. Breast Cancer *2013*, 250435.

78. Sottoriva, A., Spiteri, I., Piccirillo, S.G.M., Touloumis, A., Collins, V.P., Marioni, J.C., Curtis, C., Watts, C., and Tavaré, S. (2013). Intratumor heterogeneity in human glioblastoma reflects cancer evolutionary dynamics. Proc. Natl. Acad. Sci. USA *110*, 4009–4014.

79. Ståhl, P.L., Salmén, F., Vickovic, S., Lundmark, A., Navarro, J.F., Magnusson, J., Giacomello, S., Asp, M., Westholm, J.O., Huss, M., et al. (2016). Visualization and analysis of gene expression in tissue sections by spatial transcriptomics. Science *353*, 78–82.

80. Sun, S., Zhu, J., Mozaffari, S., Ober, C., Chen, M., and Zhou, X. (2019). Heritability estimation and differential analysis of count data with generalized linear mixed models in genomic sequencing studies. Bioinformatics *35*, 487–496.

81. Sun, S., Zhu, J., and Zhou, X. (2020). Statistical analysis of spatial expression patterns for spatially resolved transcriptomic studies. Nat. Methods *17*, 193–200.

82. Titsias, M., and Lázaro-Gredilla, M. (2011). Spike and slab variational inference for multi-task and multiple kernel learning. Adv. Neural Inf. Process. Syst. *24*.

83. Tomko, L.A., Hill, R.C., Barrett, A., Szulczewski, J.M., Conklin, M.W., Eliceiri, K.W., Keely, P.J., Hansen, K.C., and Ponik, S.M. (2018). Targeted matrisome analysis identifies thrombospondin-2 and tenascin-c in aligned collagen stroma from invasive breast carcinoma. Sci. Rep. *8*, 12941.

84. Troiani, T., Napolitano, S., Della Corte, C.M., Martini, G., Martinelli, E., Morgillo, F., and Ciardiello, F. (2016). Therapeutic value of EGFR inhibition in CRC and NSCLC: 15 years of clinical evidence. ESMO Open *1*, e000088.

85. Urpilainen, E., Puistola, U., Boussios, S., and Karihtala, P. (2020). Metformin and ovarian cancer: the evidence. Ann. Transl. Med. *8*, 1711.

86. Velten, B., and Huber, W. (2021). Adaptive penalization in high-dimensional regression and classification with external covariates using variational Bayes. Biostatistics *22*, 348–364.

87. Vidal, M., Cusick, M.E., and Barabási, A.-L. (2011). Interactome Networks and Human Disease. Cell *144*, 986–998.

88. Waks, A.G., and Winer, E.P. (2019). Breast Cancer Treatment: A Review. J. Am. Med. Assoc. *321*, 288–300.

89. Wang, Z., Kaseb, A.O., Amin, H.M., Hassan, M.M., Wang, W., Morris, J.S., and Morris, J.S. (2022). Bayesian Edge Regression in Undirected Graphical Models to Characterize Interpatient Heterogeneity in Cancer. J. Am. Stat. Assoc. *117*, 533–546.

90. Cancer Genome Atlas Research Network; Weinstein, J.N., Collisson, E.A., Mills, G.B., Shaw, K.R.M., Ozenberger, B.A., Ellrott, K., Shmulevich, I.,

Sander, C., and Stuart, J.M. (2013). The cancer genome atlas pan-cancer analysis project. Nat. Genet. *45*, 1113–1120.

91. Wijnhoven, B.P., Dinjens, W.N., and Pignatelli, M. (2000). E-cadherin—catenin cell—cell adhesion complex and human cancer. Br. J. Surg. *87*, 992–1005.

92. Yang, X., Gan, L., Narisetty, N.N., and Liang, F. (2021). Gembag: Group estimation of multiple bayesian graphical models. Journal of machine learning research *22*, 1–48.

93. Yuan, Y. (2016). Spatial heterogeneity in the tumor microenvironment. Cold Spring Harb. Perspect. Med. *6*, a026583.

94. Zhang, J., and Li, Y. (2022). High-Dimensional Gaussian Graphical Regression Models with Covariates. J. Am. Stat. Assoc. *118*, 2088–2100.

95. Zhu, L., Jiang, M., Wang, H., Sun, H., Zhu, J., Zhao, W., Fang, Q., Yu, J., Chen, P., Wu, S., et al. (2021). A narrative review of tumor heterogeneity and challenges to tumor drug therapy. Ann. Transl. Med. *9*, 1351.

STAR★METHODS

KEY RESOURCES TABLE

REAGENT or RESOURCE	SOURCE	IDENTIFIER
Deposited data		
TCPA data	Li et al., 2013[45]	https://gdc.cancer.gov/about-data/publications/pancanatlas
TCGA data	NIH Genomic Data Commons (GDC)	https://gdc.cancer.gov/about-data/publications/PanCanStemness-2018
Stemness indices data	Malta et al., 2018[52]	https://gdc.cancer.gov/about-data/publications/PanCanStemness-2018
Human breast cancer spatial transcriptomics data	Ståhl et al., 2016[79]	https://www.spatialresearch.org/resources-published-datasets/
Software and algorithms		
GraphR	This paper	https://github.com/bayesrx/GraphR
TCGAbiolinks	Colaprico et al., 2016[17]	https://bioconductor.org/packages/release/bioc/html/TCGAbiolinks.html
BGGM	Mohammadi et al., 2015[57]	https://cran.r-project.org/web/packages/BDgraph/index.html
Huge	Friedman et al., 2008[25]	https://cran.r-project.org/web/packages/huge/index.html
FGL, GGL	Danaher et al., 2014[20]	https://cran.r-project.org/web/packages/JGL/index.html
LASICH	Saegusa et al., 2016[73]	https://github.com/asondhi/LASICH
kernel graphical lasso (K-GLASSO	Liu et al., 2010[49]	https://github.com/bayesrx/GraphR
jointGHS	Lingjærde et al., 2022[48]	https://github.com/Camiling/jointGHS

METHOD DETAILS

Heterogeneous graphical regression models

Let $\mathbf{Y}$ denote a $p-$ dimensional vector of genomic features (e.g., proteins/genes) and $\mathbf{X}$ denote a $q-$ dimensional vector of intrinsic factors driving heterogeneity (e.g., cancer subtypes, spatial locations) which are assessed on the same n samples. Standard GGMs for $\mathbf{Y}$ can be represented as $\mathbf{Y} \sim N(0,\Omega^{-1})$, where $N(\cdot)$ is the Gaussian distribution. Elements in the precision matrix Ω, written as ω_{ij}, encode the conditional dependence, with zero elements denoting conditional independence.[55,69] established a connection between ω_{ij} and the coefficients of a linear regression model where Y_i $(1 \le i \le p)$ is regressed on other nodes Y_{-i}, namely, $Y_i = \sum_{j\neq i}^{p}\gamma_{ij}Y_j + \epsilon_i$. ϵ_i is independent of Y_j if and only if $\gamma_{ij} = -\omega_{ij}/\omega_{ii}$ and $\epsilon_i \sim N(0,1/\omega_{ii})$.

In GraphR we generalize this construction to $\mathbf{Y}|\mathbf{X} \sim N[0,\Omega(\mathbf{X})^{-1}]$, where-in the conditional dependence now are functions of the intrinsic factors $\mathbf{X}$ which modulate the dependence patterns of the feature vector $\mathbf{Y}$. Denote $(i,j)th$ entry of $\Omega(\mathbf{X})$ as $\omega_{ij}(\mathbf{X})$ and we assume $\omega_{ii}(\mathbf{X}) = \omega_{ii}$ for any i. We regress Y_i $(1 \le i \le p)$ on other nodes Y_{-i} where regression coefficients are function of intrinsic factors, leading to

$$Y_i = \sum_{j\neq i}^{p}\gamma_{ij}(\mathbf{X})\odot Y_j + \epsilon_i \qquad \text{(Equation 1)}$$

Here $\odot$ represents the element-wise multiplication between vectors. Under the normality assumption, we have ϵ_i is independent of Y_j given X if and only if $\gamma_{ij}(\mathbf{X}) = -\omega_{ij}(\mathbf{X})/\omega_{ii}(\mathbf{X})$ and $\epsilon_i \sim N(0,1/\omega_{ii}(\mathbf{X}))$. Based on the restated problem, a non-zero $\gamma_{ij}(\mathbf{X})$ will lead to the existence of an edge. For simplicity and interpretation, we only consider the case where off-diagonal entries are linear function of intrinsic factors, we can further express the γ_{ij} as:

$$\gamma_{ij}(X) = -\frac{\omega_{ij}(\mathbf{X})}{\omega_{ii}} = -\frac{1}{\omega_{ii}}\mathbf{X}\boldsymbol{\beta}_{\mathbf{ij}} = -\frac{1}{\omega_{ii}}\left(\sum_{l=1}^{q}\beta_{ijl}X_l\right) \qquad \text{(Equation 2)}$$

Notably, the functional form of $\gamma_{ij}(\mathbf{X})$ enables the flexibility in our models (Figure 1), i.e., (I) a standard GGM with $\mathbf{X} = (1, \ldots, 1)^T$; (II) multiple/group-specific GGMs when $\mathbf{X}$ is categorical; and (III) a GGM varying over the domain of the continuous covariate with a continuous covariate. Moreover with prior knowledge on the directionality in the nodes, the methodology can be adapted to a directed acyclic graph (DAG) models along with the incorporation of sample heterogeneity (via $\mathbf{X}$).

Priors for GraphR

We assume a covariate dependent sparse precision matrices since genomic and proteomic networks are typically sparse in nature. To this end, note that the coefficients $\beta_{ijl} = 0$ for all $\ell = 1, \ldots, q$ implies the edge between random variable Y_i and Y_j doesn't exist which leads to a sparse linear regression problem. From a Bayesian perspective, the most widely used sparse regression technique is to impose a model selection prior, such as spike-and-slab prior, which assigns probabilistic weights to each variable and shrinks noise variables to exact 0.[29] It yields an automatic edge selection property for graphical models by providing exact zero estimates for several variables, and inherits some desirable theoretical aspects.[15,60] Therefore we employ spike-and-slab priors on the coefficients of intrinsic factors with different slab precision and mixing parameters, as follows:

$$\begin{aligned} \beta_{ijl} &= b_{ijl} s_{ijl}, \\ b_{ijl} | \tau_{il} &\sim N\left(0, \tau_{il}^{-1}\right), \\ s_{ijl} | \pi_{ijl} &\sim Ber(\pi_{ijl}). \end{aligned} \qquad \text{(Equation 3)}$$

With probability π_{ijl}, β_{ijl} are drawn from $N(0, \tau_{il}^{-1})$ i.e., "slab" density with precision parameter τ_{il}, and β_{ijl} is 0 with probability $1 - \pi_{ijl}$. More specifically for $\pi_{ijl} = 1$, we will have a normal prior on the coefficient of intrinsic factor, which is equivalent to ridge regression.[86] Conjugate priors for hyper-parameters τ and π are selected:

$$\begin{aligned} \tau_{il} &\sim \Gamma(a_\tau, b_\tau), \\ \pi_{ijl} &\sim Beta(a_\pi, b_\pi). \end{aligned} \qquad \text{(Equation 4)}$$

We choose a non-informative prior for variance parameter $\omega_{ii} \propto 1$[89].

Model fitting via variational Bayes

MCMC is a standard technique for posterior inference of model parameters.[28,34] However, since the posterior distribution contains high-dimensional integrals and $2^{p*(p-1)*q}$ potential models, MCMC will be computationally challenging for large, even moderate p and q. Throughout this article, we consider a variational Bayes (VB) method[13,67] with exchangeable priors and mean-field assumption. Mean-field VB (MFVB) aims to find an approximation $q_{\text{vb}}(\boldsymbol{\theta})$ belonging to a family of distributions Q and the posterior distribution $p(\boldsymbol{\theta}|\mathbf{Y}, \mathbf{X})$[6] by minimizing the reverse Kullback–Leibler (KL) divergence, defined as $KL(q_{\text{vb}}(\boldsymbol{\theta}) \| p(\boldsymbol{\theta}|\mathbf{Y}, \mathbf{X})) = \int q_{\text{vb}}(\boldsymbol{\theta}) \log\left(\frac{q_{\text{vb}}(\boldsymbol{\theta})}{p(\boldsymbol{\theta}|\mathbf{Y}, \mathbf{X})}\right) d\boldsymbol{\theta}$. A common choice of Q, known as mean-field approximation, assumes that $q_{\text{vb}}(\boldsymbol{\theta})$ can be expressed as $\prod_{k=1}^{K} q_{\text{vb}}^k(\boldsymbol{\theta}_\mathbf{k})$ for some partition of $\boldsymbol{\theta}$. Under the mean-field assumption, the optimal q_{vb}^k is given by

$$\log\left[q_{\text{vb}}^k(\boldsymbol{\theta}_\mathbf{k})\right] = \text{Const} + \mathbb{E}_{-k}(\log p(\boldsymbol{\theta}, \mathbf{Y}, \mathbf{X})) \qquad \text{(Equation 5)}$$

In our case, due to the strong dependencies between b and s[82], we factorize the distribution as

$$q_{\text{vb}}(\mathbf{b}, \mathbf{s}, \boldsymbol{\omega}, \boldsymbol{\pi}, \boldsymbol{\tau}) = q_{\text{vb}}(\mathbf{b}, \mathbf{s}) q_{\text{vb}}(\boldsymbol{\omega}) q_{\text{vb}}(\boldsymbol{\pi}) q_{\text{vb}}(\boldsymbol{\tau}) \qquad \text{(Equation 6)}$$

More details about implementation of the VB method and posterior inference are provided in Supplementary Section S1. The GraphR algorithm is also provided in Algorithm S1.

Network construction

Networks are constructed for each group/individual via[1]: posterior expectation of magnitude ($\mathbb{E}b$) and[2] posterior inclusion probability (PIP, $\mathbb{E}s$) for each coefficient and[3] expected precision parameter ($\mathbb{E}\omega_{ii}$) obtained from GraphR results. Considering an individual with intrinsic factor $(x_1, \ldots, x_q)$, we define PIP of the edge between node j and i as a weighted mean of $\{\mathbb{E}(s_{ijl})\}_{1 \le l \le q}$ with weights proportional to $(x_l \mathbb{E} b_{ijl})^2$. An (significant) edge is included in the network if the corresponding PIP is larger than an FDR-based cutoff.[7,58] Partial correlation is calculated as $-\left[\sum_{l=1}^{q} x_l \mathbb{E}(b_{ijl} s_{ijl})\right] / \omega_{ii}$, indicating the sign and strength of the corresponding edge.

Simulated data

We assess the performance of the GraphR method in different simulation settings from undirected and directed graphs. In the first scenario, we consider undirected graphs with unordered pairs of vertices, and directed graphs in the second scenario where directions of edges are decided by some prior knowledge. For both settings, the numbers of parameters to be estimated are $\mathcal{O}(p^2 q)$ which are significantly larger than sample sizes. We focus on graph structural recovery across different settings.

Comparative methods

We compare our approach to nine relevant methods: (I) BGGM[57] (II) GLASSO[25] (III) FGL[20] (IV) GGL[20] (V) LASICH[73] (VI) Joint Graphical Horseshoe estimator (GHS,[48]) (VII) K-GLASSO[49] (VIII) Bayesian Edge Regression[89] (IX) GGMx.[61] Implementation details can be found in Supplementary Section S2.

Under categorical intrinsic factors, the proposed GraphR is equivalent to multiple graphical model which assumes that samples are drawn from G distinct groups. Thus, we compare our method with the following three commonly used methods for multiple graphical models: FGL, GGL, LASICH, GHS and Bayesian Edge Regression. In case of continuously varying graph structures over the domain of continuous intrinsic factors, we compare our method with BGGM, GLASSO, k-GLASSO and GGMx. K-GLASSO integrates intrinsic factors by kernel smoothing function and is regarded as a fair comparison in terms of GLASSO and BGGM. A standard Gaussian graphical model can be treated as a special case of GraphR with intrinsic factors being intercept only. For comparison, we consider one Bayesian and one non-Bayesian approach (i.e., BGGM and GLASSO respectively), and both methods are designed for independent and identically distributed subjects from homogeneous populations. Notably, while both Bayesian Edge Regression and GGMx can be used to infer graph structures that vary with intrinsic factors, these approaches rely on MCMC algorithms, which limits their scalability for even moderately large n and p. As a result, we compare Bayesian Edge Regression and GGMx to GraphR only in scenarios where $n = 100$ and $p = 20$.

Comparative measures

We use the following comparative measures to evaluate the graph structural recovery performance across different settings. We calculate the true positive rate (TPR), false positive rate (FPR), false discovery rate (FDR) and Matthews correlation coefficient (MCC),

$$\mathrm{TPR} = \frac{\mathrm{TP}}{\mathrm{TP}+\mathrm{FN}},\ \mathrm{FPR} = \frac{\mathrm{FP}}{\mathrm{FP}+\mathrm{TN}},\ \mathrm{FDR} = \frac{\mathrm{FP}}{\mathrm{FP}+\mathrm{TP}},$$

$$\mathrm{MCC} = \frac{\mathrm{TP}\times\mathrm{TN} - \mathrm{FP}\times\mathrm{FN}}{\sqrt{(\mathrm{TP}+\mathrm{FP})(\mathrm{TP}+\mathrm{FN})(\mathrm{TN}+\mathrm{FP})(\mathrm{TN}+\mathrm{FN})}}$$

where TP, FP, TN, and FN denote true positives, false positives, true negatives, and false negatives. MCC[54] measures the quality of binary classification, ranging from +1 (perfect classification) to -1 (total mismatch). In addition to the previous measures, we construct the receiver operating characteristic (ROC) curve to compare the settings ability to recover significant edges. The sensitivity (TPR) and 1-specificity (FPR) for each setting are computed at each threshold parameter value $[\alpha \in (0,1)]$ with the area under the ROC curve (AUC) to compare the structural recovery in each setting. Higher AUC values imply better recovery of edges. Specially we calculate the measures for each subject in the continuous case and mean values of measures are reported. Next, we discuss the simulation design and performance of the GraphR method w.r.t. undirected and directed graphs.

Scenario I: Undirected graphs

We consider undirected graphs with unordered pairs of vertices in the first scenario which contain three simulation settings. In each setting, we fix sample size $N = 151$, number of nodes $p = 33$, and connection probability $\pi = 2\%$, with the types and numbers of intrinsic factors changing with different cases.

Case A. Multi-category Graphs: Samples are evenly classified into 2 or 3 groups, and each sample has 2 or 3 discrete intrinsic factors, indicating the group allocations. Graphs for different groups are generated as following.

(1) Graph of the first group G_1 is obtained from a Erdos-Renyi graphs with connection probability being 2%.
(2) If the edge $\{i,j\}$ are connected in G_1, then the corresponding off-diagonal entries $\omega_{1ij} = \omega_{1ji}$ are uniformly drawn from $[-1,-0.5]\cup[0.5,1]$ otherwise ω_{1ij} are set to be 0. The diagonal entries ω_{1ii} are set to be 1. The simulated $\Omega_1 = [\omega_{1ij}]_{p\times p}$ from (I) is not necessarily to be positively definite, and thus we will add $0.1I$ to Ω_1 until it is positively definite. Draw independent observations from -1$N(0,\Omega_1)$
(III) $G_q(q = 2,3)$ is constructed by randomly excluding three existing edges and including three new edges from G_{q-1} for $q \geq 2$. The precision matrices and observations for are generated in the same way as step (II).

We also consider a lower-dimensional scenario where $n = 100$, $p = 20$, $q = 2$ and $\pi = 5\%$. All other steps follow the same procedure as outlined in steps (I) - (III) above.

Case B. Continuously Varying Graphs: Data are generated based on the assumed model by introducing two continuous intrinsic factors. (I) For $i<j$, 2% of coefficients for continuous intrinsic factors β_{ijl} are uniformly chose from -1 or 1 while others are set to be 0. Let the corresponding β_{jil} equal to β_{ijl}. Two continuous intrinsic factors are drawn from Uniform $(-1,1)$. We construct an individual-specific precision matrix Ω_n by fixing the diagonal entries ω_{ii} to be 1 and calculating $\omega_{nij} = \sum_{l=1}^{2}\beta_{nijl}X_{nl}$. (II) Repeat step (I) until Ω_n is positive definite for all n. (III) Generate y_n from $N(0,\Omega_n^{-1})$.

Case C. Homogeneous Graphs: We assume that all individuals are homogeneous, implying $\Omega_n = \Omega,\ \forall\ n \in \{1,\ldots,151\}$. We only include one constant effect as the intrinsic factor, and generate data as following:

(1) Generate a Erdős–Rényi graph G by setting connection probability 2%. If the edge $\{i,j\}$ are connected in G, then the corresponding off-diagonal entries $\omega_{ij} = \omega_{ji}$ are uniformly drawn from $[-1,-0.5]\cup[0.5,1]$ otherwise ω_{ij} are set to be 0. The diagonal entries ω_{ii} are set to be 1.

(2) The simulated Ω from (I) is not necessarily to be positively definite, and thus we will add $0.1I_{33}$ to Ω until it is positively definite.
(3) Generate 151 independent observations from $N(0,\Omega^{-1})$ and set intrinsic factors to be intercept only.

Similarly, we include a lower-dimensional scenario where $n = 100$, $p = 20$, $q = 1$ and $\pi = 5\%$. All other steps follow the same procedure as outlined in steps (I) - (III) above.

Scenario II: Directed acyclic graphs

In scenario II, we consider a special case of our GraphR in which pre-knowledge based directions of edges are assumed, leading to a directed acyclic graph model. We consider different simulation settings to illustrate the performance of variable selection and scalability in case of moderate and/or large number of nodes (p) and intrinsic factors (q). Data is generated as following.

(1) Based on the proposed types of intrinsic factors, we generate continuous intrinsic factors from Uniform(0,1) and discrete intrinsic factors from Bernoulli(0.5). In the settings with large number of intrinsic factors, 70% of intrinsic factors are set to be continuous.
(2) For each intrinsic factor, 2% second layer of regression coefficients $\beta_{ij}^{(k)}$ are randomly selected to be non-zero and are set to be 3 when $ji>$.
(3) The first node $(i = 1)$ is generated from $N(0,1)$. In terms of ith node Y_i $(i \geq 2)$, we standardize Y_j for all $j<i$, and denote these standardized nodes as $\tilde{Y}_j$. Draw Y_i from Normal distribution with mean being $\sum_{j<i}[(\beta_{ij}^{(1)}Z^{(1)} + \beta_{ij}^{(2)}Z^{(2)}) \odot \tilde{Y}_j]$ and standard deviation being 1.

Simulation design for Simpon's paradox

We set overall sample size as 200, number of nodes as 3 and number of groups as 2. Samples are evenly drawn from two groups with distinct conditional independence structures. Generation of precision matrices $\Omega, \tilde{\Omega} \in \mathbb{R}^{3\times3}$ for the first and the second group is described as follow:

(1) Denote $\Omega = [\omega_{ij}]_{3\times3}$. Three diagonal entries are set as 1. For off-diagonal entries, $\omega_{12} = \omega_{21}$ are uniformly drawn from [0.5,1], $\omega_{13} = \omega_{31}$ are uniformly drawn from $[-1, -0.5]$, and $\omega_{23} = \omega_{32}$ are set to be 0.
(II) Denote $\tilde{\Omega} = [\tilde{\omega}_{ij}]_{3\times3}$. Three diagonal entries are set as 1. For off-diagonal entries, $\tilde{\omega}_{12} = \tilde{\omega}_{21}$ are uniformly drawn from $[-1, -0.5]$, $\tilde{\omega}_{23} = \tilde{\omega}_{32}$ are uniformly drawn from [0.5,1], and $\tilde{\omega}_{13} = \tilde{\omega}_{31}$ are set to be 0.

Repeat (I) and (II) until both $\Omega, \tilde{\Omega}$ are positive definite matrices.

(1) Generate 100 observations from $N(0,\Omega^{-1})$ and 100 observations from $N(0,\tilde{\Omega}^{-1})$, and set intrinsic factors to be intercept only for homogeneous case and to be group indicators for heterogeneous case.

Preprocessing for application

Intrinsic subtypes-specific proteomic network

Proteomics data is standardized before applying GraphR. The indicators for cancer subtype (Luminal A + B, Her2-enriched and Basal-like breast cancers) are used as intrinsic factors. Same preprocessing steps are applied for pan-cancer analyses across gynecological cancers.

Stemness-induced proteomic network

The mRNAsi, mDNAsi and patients' ages are treated as three intrinsic factors. Logit transformation is used to the stemness indices to ensure the same scale as age, and all three intrinsic factors and proteomics data are standardized before plugging into the model.

Genomic network incorporating spatial heterogeneity

: We only consider the 100 spatially expressed genes with the lowest Benjamini-Hochberg (BH) adjusted p-value by applying SPARK method.[81] Next, we apply the PQLseq[80] algorithm to adjust for the covariate effect and obtain the latent gene expressions which follow Normal distribution. Two coordinates are scaled and treated as intrinsic factors in the GraphR model.

Hyper-parameters are set as follows: $a_\tau = b_\tau = 0.005$, $a_\pi = 1$, $b_\pi = 4$. Notably, in case of integrated functional pathway analysis, we change the hyper-parameters: $a_\pi = b_\pi = 0.05$. This provides us potential to consider high density of connections between proteins in the same pathway.

Network summaries

Downstream analyses, e.g., hub node detection and pathway-based functional analysis, are conducted based on constructed networks. Hub genes are detected based on the *connectivity degree* which is defined as the sum of magnitudes of significant edges (i.e., absolute values of partial correlations) between the corresponding node and others. Aiming to evaluate the connectivity status of a pathway, we apply *connectivity score (CS)*, which is calculated as the ratio of the observed number of edges between proteins belonging to the corresponding pathway to the total number of possible edges.

For spatial-varying genomic networks, the weighted average of partial correlations between gene i and gene j in a region is defined as follow:

Assume the partial correlations between gene *i* and gene *j* and the corresponding posterior inclusion probabilities are vectors of length $n_1 + n_2 + n_3$ with n_1, n_2, n_3 representing number of spots in tumor, intermediate and normal region, namely

$$\rho_{ij} = \left[\rho_{ij}^{tumor}, \rho_{ij}^{inter}, \rho_{ij}^{normal}\right] \in \mathbb{R}^{n_1+n_2+n_3},$$
$$PIP_{ij} = \left[PIP_{ij}^{tumor}, PIP_{ij}^{inter}, PIP_{ij}^{normal}\right] \in \mathbb{R}^{n_1+n_2+n_3}. \quad \text{(Equation 7)}$$

We define weighted average of partial correlations between gene *i* and gene *j* in a region as $\widehat{\rho}_{ij}^{region} = \left(\sum_{region} \rho_{ij}^{region} * PIP_{ij}^{region}\right) \Big/ n_{region}$ where region can be tumor, intermediate or normal. Weighted connectivity degree of gene *i* is defined as the sum of $|\widehat{\rho}_{i\cdot}|$.

QUANTIFICATION AND STATISTICAL ANALYSIS

All statistics were conducted using R and are described in the appropriate sections in the result section and STAR Methods.

Cell Reports Methods, Volume *5*

Supplemental information

A probabilistic modeling framework for genomic networks incorporating sample heterogeneity

Liying Chen, Satwik Acharyya, Chunyu Luo, Yang Ni, and Veerabhadran Baladandayuthapani

GraphR: a probabilistic modeling framework for genomic networks incorporating sample heterogeneity

Liying Chen[1,*], Satwik Acharyya[2,*], Chunyu Luo[3],
Yang Ni[4], Veerabhadran Baladandayuthapani[1,†]

[1]Department of Biostatistics, University of Michigan, Ann Arbor, MI

[2]Department of Biostatistics, University of Alabama at Birmingham, Birmingham, AL

[3]Division of Biostatistics, University of Pennsylvania, Philadelphia, PA

[4]Department of Statistics, Texas A&M University, College Station, TX

January 13, 2025

*These authors contributed equally.

†Corresponding author: veerab@umich.edu.

Methods S1: Derivation of GraphR, related to Figure 1

The likelihood is expressed as

$$\begin{aligned}
p(\boldsymbol{\theta}, \boldsymbol{Y}, \boldsymbol{X}) \propto & \prod_{i=1}^{p} \left\{ \left| \frac{1}{\omega_{ii}} I_n \right|^{-\frac{1}{2}} \exp\left[-\frac{\omega_{ii}}{2} \left\| Y_i - \sum_{j \neq i} \gamma_{ij}(X) \odot Y_j \right\|^2 \right] \right\} \times \\
& \prod_{i=1}^{p} \prod_{j \neq i}^{p} \prod_{l=1}^{q} \left\{ \left(\frac{1}{\tau_{il}} \right)^{-\frac{1}{2}} \exp\left[-\frac{\tau_{il}}{2} (b_{ijl})^2 \right] (\pi_{ijl})^{s_{ijl}} (1-\pi_{ijl})^{1-s_{ijl}} \right\} \times \\
& \prod_{i=1}^{p} \prod_{l=1}^{q} \left\{ (\tau_{il})^{a_\tau - 1} \exp\left[-b_\tau \tau_{il} \right] \right\} \times \\
& \prod_{i=1}^{p} \prod_{j \neq i}^{p} \prod_{l=1}^{q} \left\{ (\pi_{ijl})^{a_\pi - 1} (1-\pi_{ijl})^{b_\pi - 1} \right\} \qquad \text{(A.5)}
\end{aligned}$$

$$\begin{aligned}
\log p(\boldsymbol{\theta}, \boldsymbol{Y}, \boldsymbol{X}) = & \text{Const} + \sum_{i=1}^{p} \left\{ \frac{n}{2} \log(\omega_{ii}) - \frac{\omega_{ii}}{2} \left\| Y_i + \frac{1}{\omega_{ii}} \sum_{j \neq i}^{p} \sum_{l=1}^{q} b_{ijl} s_{ijl} X_l \odot Y_j \right\|^2 \right\} \\
& + \sum_{i=1}^{p} \sum_{l=1}^{q} \left\{ \left(\frac{p-1}{2} + a_\tau - 1 \right) \log(\tau_{il}) - \left(b_\tau + \frac{1}{2} \sum_{j \neq i}^{p} (b_{ijl})^2 \right) \tau_{il} \right\} \\
& + \sum_{i=1}^{p} \sum_{j \neq i}^{p} \sum_{l=1}^{q} \left\{ (s_{ijl} + a_\pi - 1) \log(\pi_{ijl}) + (b_\pi - s_{ijl}) \log(1-\pi_{ijl}) \right\}. \qquad \text{(A.6)}
\end{aligned}$$

Due to the dependence between $\boldsymbol{b}$ and $\boldsymbol{s}$[14], the mean-field assumption is considered as:

$$q_{\text{vb}}(\boldsymbol{b}, \boldsymbol{s}, \boldsymbol{\omega}, \boldsymbol{\pi}, \boldsymbol{\tau}) = q_{\text{vb}}(\boldsymbol{b}, \boldsymbol{s}) q_{\text{vb}}(\boldsymbol{\omega}) q_{\text{vb}}(\boldsymbol{\pi}) q_{\text{vb}}(\boldsymbol{\tau}).$$

We can obtain the update equation for each parameter as:

$$q_{\text{vb}}^{k}(\boldsymbol{\theta}_{\boldsymbol{k}}) \propto \exp\left[\mathbb{E}_{-k}(\log p(\boldsymbol{\theta}, \boldsymbol{Y}, \boldsymbol{X})) \right].$$

a. Update of τ_{il}:

$$\begin{aligned}
\log q_{\text{vb}}(\tau_{il}) &= \mathbb{E}_{-\tau_{il}}(l) \\
&= C + \left[\frac{p-1}{2} + a_\tau - 1 \right] \log\tau_{il} + \left[b_\tau + \frac{1}{2} \sum_{j \neq i}^{p} \mathbb{E}_{-\tau_{il}} (b_{ijl})^2 \right] \tau_{il}. \qquad \text{(A.7)}
\end{aligned}$$

$$q_{\text{vb}}(\tau_{il}) \sim \Gamma\left(a_\tau + \frac{p-1}{2}, b_\tau + \frac{1}{2} \sum_{j \neq i}^{p} \mathbb{E}_{-\tau_{il}} (b_{ijl})^2 \right)$$

b. Update of π_{ijl}:

$$\begin{aligned}\log\, q_{\text{vb}}(\pi_{ijl}) &= \mathbb{E}_{-\pi_{ijl}}(l)\\ &= C + \left[\mathbb{E}_{-\pi_{ijl}}(s_{ijl}) + a_\pi - 1\right]\log(\pi_{ijl}) + \left[b_\pi - \mathbb{E}_{-\pi_{ijl}}(s_{ijl})\right]\log(1-\pi_{ijl}).\end{aligned} \quad \text{(A.8)}$$

$$q_{\text{vb}}(\pi_{ijl}) \sim \text{Beta}\left(\mathbb{E}_{-\pi_{ijl}}(s_{ijl}) + a_\pi, b_\pi - \mathbb{E}_{-\pi_{ijl}}(s_{ijl}) + 1\right)$$

c. Update of ω_{ii}:

$$\begin{aligned}\log\, q_{\text{vb}}(\omega_{ii}) &= \mathbb{E}_{-\omega_{ii}}(l)\\ &= C + \frac{n}{2}\log(\omega_{ii}) - \frac{\|Y_i\|^2}{2}\omega_{ii} - \frac{\mathbb{E}_{-\omega_{ii}}\|\sum_{j\neq i}^{p}\sum_{l=1}^{q} b_{ijl}s_{ijl}X_l\odot Y_j\|^2}{2}\left(\frac{1}{\omega_{ii}}\right).\end{aligned} \quad \text{(A.9)}$$

$$q_{\text{vb}}(\omega_{ii}) \sim \text{GIG}\left(\frac{n+2}{2}, \|Y_i\|^2, \mathbb{E}_{-\omega_{ii}}\|\sum_{j\neq i}^{p}\sum_{l=1}^{q} b_{ijl}s_{ijl}X_l\odot Y_j\|^2\right)$$

Here **GIG** represents generalized inverse Gaussian distribution.

d. Update of $\beta_{ijl} = b_{ijl}s_{ijl}$:
We Denote $M_{-(m,n)}^{-k} = \sum_{j\neq m}^{p}\sum_{l=1}^{q} b_{mjl}s_{mjl}X_l\odot Y_j - b_{mnk}s_{mnk}X_k\odot Y_n$ and

$$\begin{aligned}\log\, q_{\text{vb}}(b_{ijl}|s_{ijl}) &= \mathbb{E}_{-b_{ijl}|s_{ijl}}(l)\\ &= C - \frac{1}{2}\left[\mathbb{E}_{-b_{ijl}|s_{ijl}}(\tau_{il}) + \mathbb{E}_{-b_{ijl}|s_{ijl}}\left(\frac{1}{\omega_{ii}}\right)s_{ijl}\|X_l\odot Y_j\|^2\right](b_{ijl})^2\\ &\quad - \left[Y_i + \mathbb{E}_{-b_{ijl}|s_{ijl}}\left(\frac{1}{\omega_{ii}}\right)\mathbb{E}_{-b_{ijl}|s_{ijl}}M_{-(i,j)}^{-l}\right]^T [X_l\odot Y_j]\, s_{ijl}b_{ijl}.\end{aligned} \quad \text{(A.10)}$$

$$q_{\text{vb}}(b_{ijl}|s_{ijl}) \sim \mathbb{N}\left(\mu(s_{ijl}), \sigma^2(s_{ijl})\right)$$

$$\sigma^2(s_{ijl}) = \left[\mathbb{E}_{-b_{ijl}|s_{ijl}}(\frac{1}{\omega_{ii}})\|X_l\odot Y_j\|^2 s_{ijl} + \mathbb{E}_{-b_{ijl}|s_{ijl}}(\tau_{il})\right]^{-1}$$

$$\mu(s_{ijl}) = -\sigma^2(s_{ijl})\left\{\left[Y_i + \mathbb{E}_{-b_{ijl}|s_{ijl}}\left(\frac{1}{\omega_{ii}}M_{-(i,j)}^{-l}\right)\right]^T [X_l\odot Y_j]s_{ijl}\right\}$$

The mariginal density of $q_{\text{vb}}(s_{ijl})$ is obtained by integrating the joint density of $q_{\text{vb}}(b_{ijl}, s_{ijl})$

as

$$
\begin{aligned}
q_{\mathrm{vb}}(s_{ijl}) &= \int \exp\left\{\log\, q_{\mathrm{vb}}(b_{ijl}, s_{ijl})\right\} db_{ijl} \\
&= \exp\left\{s_{ijl}\ \mathbb{E}_{-s_{ijl}}\mathrm{logit}(\pi_{ijl})\right\} \int \mathbb{N}_{b_{ijl}}\left(\mu(s_{ijl}), \sigma^2(s_{ijl})\right)\sigma(s_{ijl})\mathrm{exp}\ \left(\frac{\mu^2(s_{ijl})}{2\sigma^2(s_{ijl})}\right) db_{ijl} \\
&= \sigma(s_{ijl})\mathrm{exp}\left\{s_{ijl}\ \mathbb{E}_{-s_{ijl}}\mathrm{logit}(\pi_{ijl}) + \left(\frac{\mu^2(s_{ijl})}{2\sigma^2(s_{ijl})}\right)\right\}.
\end{aligned}
$$

$$
\log[q_{\mathrm{vb}}(s_{ijl})] = C + \log(\sigma(s_{ijl})) + s_{ijl}\ \mathbb{E}_{-s_{ijl}}\mathrm{logit}(\pi_{ijl}) + \frac{\mu^2(s_{ijl})}{2\sigma^2(s_{ijl})}
$$

$$
\log[q_{\mathrm{vb}}(s_{ijl}=0)] = C - \frac{1}{2}\log\mathbb{E}_{-s_{ijl}}\tau_{ijl}
$$

$$
\begin{aligned}
\log[q_{\mathrm{vb}}(s_{ijl}=1)] &= C + \mathbb{E}_{-s_{ijl}}\mathrm{logit}(\pi_{ijl}) - \frac{1}{2}\log\left[\mathbb{E}_{-s_{ijl}}(\frac{1}{\omega_{ii}})\|X_l\odot Y_j\|^2 + \mathbb{E}_{-s_{ijl}}(\tau_{il})\right] \\
&+ \frac{1}{2}\left[\mathbb{E}_{-s_{ijl}}(\frac{1}{\omega_{ii}})\|X_l\odot Y_j\|^2 + \mathbb{E}_{-s_{ijl}}(\tau_{il})\right]^{-1}\left[(X_l\odot Y_j)^T(Y_i + \mathbb{E}_{-s_{ijl}}\left(\frac{1}{\omega_{ii}}\right)\mathbb{E}_{-s_{ijl}}M^{-l}_{-(i,j)})\right]^2
\end{aligned}
$$

$$
\begin{aligned}
& s_{ijl} \sim \mathrm{Ber}(\psi_{ijl}) \\
& \log\, q_{\mathrm{vb}}(s_{ijl}) = C + s_{ijl}\mathrm{logit}(\psi_{ijl}) \\
& \psi_{ijl} = \mathbb{E}_{-s_{ijl}}\mathrm{logit}(\pi_{ijl}) - \frac{1}{2}log\left[\mathbb{E}_{-s_{ijl}}(\frac{1}{\omega_{ii}})\|X_l\odot Y_j\|^2 + \mathbb{E}_{-s_{ijl}}(\tau_{il})\right] + \frac{1}{2}log\mathbb{E}_{-s_{ijl}}\tau_{il} \\
& + \frac{1}{2}\left[\mathbb{E}_{-s_{ijl}}(\frac{1}{\omega_{ii}})\|X_l\odot Y_j\|^2 + \mathbb{E}_{-s_{ijl}}(\tau_{il})\right]^{-1}\left[(X_l\odot Y_j)^T(Y_i + \mathbb{E}_{-s_{ijl}}\left[\frac{1}{\omega_{ii}}\right]\mathbb{E}_{-s_{ijl}}M^{-l}_{-(i,j)})\right]^2
\end{aligned}
$$

The evidence lower bound is defined as:

$$
\begin{aligned}
L[q_{\mathrm{vb}}(\boldsymbol{\theta})] &= \int q_{\mathrm{vb}}(\boldsymbol{\theta})\log\left(p(\boldsymbol{\theta}, \boldsymbol{Y}, \boldsymbol{X})/q_{\mathrm{vb}}(\boldsymbol{\theta})\right) d\boldsymbol{\theta} \\
&= \mathbb{E}_{q_{\mathrm{vb}}(\boldsymbol{\theta})}\ \log(p(\boldsymbol{\theta}, \boldsymbol{Y}, \boldsymbol{X})) - \mathbb{E}_{q_{\mathrm{vb}}(\boldsymbol{\theta})}[q_{\mathrm{vb}}(\boldsymbol{\theta})] \\
&= \mathbb{E}_{q_{\mathrm{vb}}(\boldsymbol{\theta})}\ \log(p(\boldsymbol{\theta}, \boldsymbol{Y}, \boldsymbol{X})) - \sum_{i=1}^{p}\mathbb{E}_{q_{\mathrm{vb}}(\boldsymbol{\theta})}[q_{\mathrm{vb}}(\omega_{ii})] - \sum_{i=1}^{p}\sum_{l=1}^{q}\mathbb{E}_{q_{\mathrm{vb}}(\boldsymbol{\theta})}[q_{\mathrm{vb}}(\tau_{il})] \\
&- \sum_{i=1}^{p}\sum_{j\neq i}^{p}\sum_{l=1}^{q}\left\{\mathbb{E}_{q_{\mathrm{vb}}(\boldsymbol{\theta})}[q_{\mathrm{vb}}(b_{ijl}, s_{ijl})] + \mathbb{E}_{q_{\mathrm{vb}}(\boldsymbol{\theta})}[q_{\mathrm{vb}}(\pi_{ijl})]\right\}.
\end{aligned}
$$

Notably, $-\mathbb{E}_{q_{\mathrm{vb}}(\boldsymbol{\theta})}[q_{\mathrm{vb}}(\omega_{ii})]$, $-\mathbb{E}_{q_{\mathrm{vb}}(\boldsymbol{\theta})}[q_{\mathrm{vb}}(\tau_{il})]$, $-\mathbb{E}_{q_{\mathrm{vb}}(\boldsymbol{\theta})}[q_{\mathrm{vb}}(b_{ijl}, s_{ijl})]$, $-\mathbb{E}_{q_{\mathrm{vb}}(\boldsymbol{\theta})}[q_{\mathrm{vb}}(\pi_{ijl})]$ are entropies of GIG, Gamma, Normal, Bernoulli and Beta distributions, which have a close form. We denote entropy as $H(\cdot)$ and all the expectations below are taken w.r.t $q_{\mathrm{vb}}(\boldsymbol{\theta})$.

a. Derivation of $\mathbb{E}\ \log(p(\boldsymbol{\theta}, \boldsymbol{Y}, \boldsymbol{X}))$:

$$
\begin{aligned}
\mathbb{E}\ \log(p(\boldsymbol{\theta}, \boldsymbol{Y}, \boldsymbol{X})) = & \sum_{i=1}^{p} \Bigg\{ -\frac{n+(p-1)q}{2}\log 2\pi + q\left[a_\tau \log b_\tau - \log\Gamma(a_\tau)\right] \\
& + (p-1)q\left[\log\Gamma(a_\pi + b_\pi) - \log\Gamma(a_\pi b_\pi)\right] \\
& + \frac{n}{2}\mathbb{E}(\log\omega_{ii}) - \frac{\|Y_i\|^2}{2}\mathbb{E}\omega_{ii} - \mathbb{E}(\omega_{ii}^{-1})\mathbb{E}\left\|\sum_{j\neq i}^{p}\sum_{l=1}^{q}\beta_{ijl}Z_l \odot Y_j\right\|^2 \\
& - Y_i^T\left(\sum_{j\neq i}^{p}\sum_{l=1}^{q}\mathbb{E}\beta_{ijl}Z_l \odot Y_j\right) \\
& + \sum_{l=1}^{q}\left[\left(\frac{p-1}{2} + a_\tau - 1\right)\mathbb{E}\left(\log\tau_{il}\right) - \left(b_\tau + \frac{\sum_{j\neq i}^{p}\mathbb{E}b_{ijl}^2}{2}\right)\mathbb{E}\tau_{il}\right] \\
& + \sum_{j\neq i}^{p}\sum_{l=1}^{q}\left[\left(\mathbb{E}s_{ijl} + a_\pi - 1\right)\mathbb{E}(\log\pi_{ijl}) + \left(b_\pi - \mathbb{E}s_{ijl}\right)\right]\mathbb{E}\left(\log(1-\pi_{ijl})\right)\Bigg\}
\end{aligned}
$$

b. Derivation of $H(\tau_{il})$

$$
\begin{aligned}
H(\tau_{il}) = & -\left[a_\tau + \frac{p-1}{2}\right]\log\left[b_\tau + \frac{1}{2}\sum_{j\neq i}^{p}\mathbb{E}b_{ijl}^2\right] + \log\,\Gamma(a_\tau + \frac{p-1}{2}) \\
& - \left[a_\tau + \frac{p-1}{2} - 1\right]\mathbb{E}(\log\,\tau_{il}) + \left[b_\tau + \frac{1}{2}\sum_{j\neq i}^{p}\mathbb{E}b_{ijl}^2\right]\mathbb{E}\tau_{il}
\end{aligned}
$$

c. Derivation of $H(\pi_{ijl})$

$$
\begin{aligned}
H(\pi_{ijl}) = & -\log\,\Gamma(a_\pi + b_\pi + 1) + \log\,\Gamma(\mathbb{E}s_{ijl} + a_\pi) + \log\,\Gamma(b_\pi + 1 - \mathbb{E}s_{ijl}) \\
& - (\mathbb{E}s_{ijl} + a_\pi - 1)\mathbb{E}\log\,\pi_{ijl} - (b_\pi - \mathbb{E}s_{ijl})\mathbb{E}\log\,(1-\pi_{ijl})
\end{aligned}
$$

d. Derivation of $H(\omega_{ii})$

Denote $a_{\omega_{ii}} = \|Y_i\|^2$ and $b_{\omega_{ii}} = \mathbb{E}\|\sum_{j\neq i}^{p}\sum_{l=1}^{q}\beta_{ijl}Z_l \odot Y_j\|^2$

$$
H(\omega_{ii}) = -\frac{n+2}{4}\log\frac{a_{\omega_{ii}}}{b_{\omega_{ii}}} + \log(2K_{(n+2)/2}\sqrt{a_{\omega_{ii}}b_{\omega_{ii}}}) - \frac{n}{2}\mathbb{E}(\log\omega_{ii}) + \frac{a_{\omega_{ii}}}{2}\mathbb{E}\omega_{ii} + \frac{b_{\omega_{ii}}}{2}\mathbb{E}\omega_{ii}^{-1}
$$

e. Derivation of $H(b_{ijl}, s_{ijl})$

$$
H(b_{ijl}, s_{ijl}) = H(b_{ijl}|s_{ijl}) + H(s_{ijl})
$$

where

$$H(b_{ijl}|s_{ijl}) = \frac{1}{2}\log\left[2\pi\sigma^2(s_{ijl})\right] + \frac{1}{2}$$

$$H(s_{ijl}) = -\psi_{ijl}\ \log(\psi_{ijl}) - (1-\psi_{ijl})\ \log(1-\psi_{ijl})$$

Combining all the previous entropy derivations, we have the following expression of evidence lower bound (ELBO) as

$$\begin{aligned}
L[q_{\text{vb}}(\boldsymbol{\theta})] =& -\frac{np+p(p-1)q}{2}\log 2\pi \\
&+ pq\left[a_\tau \log b_\tau - \log\Gamma(a_\tau) + \log\Gamma(a_\tau + \frac{p-1}{2})\right] \\
&+ p(p-1)q\left[\log\Gamma(a_\pi + b_\pi) - \log\Gamma(a_\pi b_\pi) - \log\Gamma(a_\pi + b_\pi + 1)\right] \\
&- \sum_{i=1}^{p}\left\{Y_i^T\left(\sum_{j\neq i}^{p}\sum_{l=1}^{q}\mathbb{E}\beta_{ijl}Z_l \odot Y_j\right) - \frac{n+2}{4}\log\frac{a_{\omega_{ii}}}{b_{\omega_{ii}}} + \log(2K_{(n+2)/2}\sqrt{a_{\omega_{ii}}b_{\omega_{ii}}})\right\} \\
&- \left[a_\tau + \frac{p-1}{2}\right]\sum_{i=1}^{p}\sum_{l=1}^{q}\left\{\log\left[b_\tau + \frac{1}{2}\sum_{j\neq i}^{p}\mathbb{E}b_{ijl}^2\right]\right\} \\
&+ \sum_{i=1}^{p}\sum_{j\neq i}^{p}\sum_{l=1}^{q}\Bigg\{\log\ \Gamma(\mathbb{E}s_{ijl} + a_\pi) + \log\ \Gamma(b_\pi + 1 - \mathbb{E}s_{ijl}) \\
&+ \frac{1}{2}\log\left[2\pi\sigma^2(s_{ijl})\right] + \frac{1}{2} - \psi_{ijl}\ \log(\psi_{ijl}) - (1-\psi_{ijl})\ \log(1-\psi_{ijl})\Bigg\}.
\end{aligned}$$

Methods S2: Implementation details of GraphR, FGL, GGL, LASICH, jointGHS, Bayesian Edge Regression, BGGM, GLASSO, k-GLASSO, related to Figure 3.

- **FGL and GGL**: Tuning parameters λ_1 and λ_2 are selected from a 20×20 grid, evenly spaced between 0.05 to 0.5 for λ_1 and 0.001 to 0.01 for λ_2, using the approximated Akaike Information Criterion (AIC). The ROC curve is generated by varying the tuning parameters λ_1 and λ_2.

- **LASICH**: Tuning parameters are selected from a 50×50 grid, evenly spaced between 1 to 50 for both λ_1 and λ_2, using the Bayesian Information Criterion (BIC). The ROC curve is generated by varying the tuning parameters λ_1 and λ_2.

- **jointGHS**: The algorithm is implemented using the `jointGHS` package, with 'epsilon' = 0.001 and 'AIC_eps' = 0.001. The ROC curve is generated by varying the tuning parameter τ^2, with 100 evenly spaced values ranging from 0.001 to 20.

- **Bayesian Edge Regression**: MCMC is run for 20,000 iterations after a burn-in of 10,000, with thinning applied to retain every 10th sample.

- **BGGM**: The model is run for 10,000 iterations after a burn-in of 5,000. The ROC curve is obtained based on different thresholds of posterior probabilities for edge inclusion.

- **GLASSO**: The tuning parameter λ is selected using the stability approach[6]. The ROC curve is generated by varying the tuning parameter λ.

- **k-GLASSO**: Tuning parameters λ_i and h_i for each subject are selected using AIC, based on a 20×20 grid evenly spaced between 0.1 to 1. The ROC curve is generated based on varying the tuning parameters λ_i and h_i for each subject.

- **GraphR**: We are interested in the parameter selection on both intrinsic factors and edges level. Point estimators of each model parameters are obtained by using expectation with respect to the approximation of posterior distribution, for example the point estimates $\hat{\pi}_{ijl} = \int \pi_{ijl}\ \hat{q}(\pi_{ijl})d\pi_{ijl} = E_{\hat{q}}(\pi_{ijl})$. With respect to selection of intrinsic factors, we use posterior inclusion probability (PIP) of intrinsic factors, which are defined as $\hat{\boldsymbol{p}}_{\boldsymbol{ij}} := [\hat{p}_{ij1}, ...\hat{p}_{ijq}]^T = [E_{\hat{q}}(s_{ij1}), ...E_{\hat{q}}(s_{ijq})]^T$. For the edge selection between Y_i and Y_j, we take both the magnitude of external coefficient $E_{\hat{q}}(\boldsymbol{b}_{ij})$ and PIP of intrinsic factors $E_{\hat{q}}(\boldsymbol{s}_{ij})$ into consideration, and thus define PIP of edges as $\phi_{ij} := \sum_{l=1}^{q}\left[\frac{\|E_{\hat{q}}(b_{ijl}s_{ijl})X_l\|^2}{\sum_{m=1}^{q}\left(\|E_{\hat{q}}(b_{ijm}s_{ijm})X_m\|^2\right)}\hat{p}_{ijl}\right]$. In the undirected graphical models, we have $\omega_{ij}(\mathbf{X}) = \omega_{ji}(\mathbf{X})$ due to the symmetric property of precision matrix, implying that s_{ijl} and s_{jil} should be 1 or 0 simultaneously. However we parallelize the regressions which leads to significant increase in time-efficacy and also breaks the dependence between s_{ijl} and s_{jil}. Thus $s_{ijl} = s_{jil}$ are not necessarily held during the estimation procedure. To ensure the symmetry of selection indicator, namely $\boldsymbol{s_{ij}} = \boldsymbol{s_{ji}}$, we use a

post-processing based approach following the steps from[7]. We denote κ_{ex} and κ_{edge} as the threshold of intrinsic factors and edge detection, where intrinsic factors will be selected or conditional dependence exists between edges if the corresponding PIP is larger than κ_{ex} and κ_{edge}. β_{ijl} is non-zero if and only if $\hat{p}_{ijl}^{min} := min(\hat{p}_{ijl}, \hat{p}_{jil}) > \kappa_{ex}$. Similarly, in the edge detection, $\phi_{ij}^{min} := min(\phi_{ij}, \phi_{ji}) > \kappa_{edge} \Leftrightarrow$ edge $\{i, j\}$ exists for $1 \leq i \neq j \leq p$. In terms of the selection of threshold on both external covariate level and edge level, we considered a Bayesian local FDR-based inference approach aiming to control the average Bayesian FDR at some level α[1,8]. Denote the threshold on external covariates or on edges at level α as $\kappa_{ex,\alpha}$ and $\kappa_{edge,\alpha}$ respectively, and denote $q_{ijl} = 1 - \hat{p}_{ijl}^{min}$ and $\tilde{q}_{ij} = 1 - \phi_{ij}^{min}$. Notably, $q_{ijl} = q_{jil}$ and $\tilde{q}_{ij} = \tilde{q}_{ji}$ due to the definition of $\hat{p}_{ijl}^{min}$ and ϕ_{ij}^{min}. PIPs of external covariates and edges represented that posterior probability that the corresponding external covariates or edges were presented from the model, then q_{ijl} and $\tilde{q}_{ij}$ can be regard as estimates of the local FDR for external covariates and edges, which are also known as Bayesian q-values[13]. This statement holds even if we have correlated data[3,1]. Based on q-values of external covariates, $\kappa_{ex,\alpha}$ is determined using follow procedure: (1) sort $\{q_{ijl}\}_{1\leq i<j\leq p, 1\leq l\leq q}$ in an ascending order and notated the ordered set as $\{q^{(t)}\}_{1\leq t\leq p(p-1)q/2}$ (2) Calculate the cumulative mean of $\{q^{(t)}\}$, and find the maximum t^* such that the cumulative mean of $q^{(t^*)}$ is smaller than α, a given significance level. (3) Set $\kappa_{ex,\alpha} = 1 - q^{(t^*)}$. Similar procedure were proposed when choosing $\kappa_{edge,\alpha}$ by replacing $\{q_{ijl}\}_{1\leq i<j\leq p, 1\leq l\leq q}$ with $\{\tilde{q}_{ij}\}_{1\leq i<j\leq p}$.

Algorithm 1 GraphR algorithm

Input: $\mathbf{Y}, \mathbf{X}$, tolerance
Output: Covariate dependent edges
while $\zeta >$ tolerance **do**
  for i in $1:p$ **do**
    for l in $1:q$ **do**
      Set $q_{\text{vb}}(\tau_{il}) \sim \Gamma\left(a_\tau + \frac{p-1}{2}, b_\tau + \frac{1}{2}\sum_{j\neq i}^{p} \mathbb{E}_{-\tau_{il}}\left(b_{ijl}\right)^2\right)$
    end for
    for j in $1:p$ and $j \neq i; l$ in $1:q$ **do**
      Set $q_{\text{vb}}(\pi_{ijl}) \sim \text{Beta}\left(\mathbb{E}_{-\pi_{ijl}}(s_{ijl}) + a_\pi, b_\pi - \mathbb{E}_{-\pi_{ijl}}(s_{ijl}) + 1\right)$
    end for
    Set $q_{\text{vb}}(\omega_{ii}) \sim \text{GIG}\left(\frac{n+2}{2}, \|Y_i\|^2, \mathbb{E}_{-\omega_{ii}}\|\sum_{j\neq i}^{p}\sum_{l=1}^{q} b_{ijl}s_{ijl}X_l \odot Y_j\|^2\right)$
    for j in $1:p$ and $j \neq i; l$ in $1:q$ **do**
      $\beta_{ijl} = b_{ijl}s_{ijl}$
      Set $q_{\text{vb}}(b_{ijl}|s_{ijl}) \sim \mathbb{N}\left(\mu(s_{ijl}), \sigma^2(s_{ijl})\right)$ where
      $\sigma^2(s_{ijl}) = \left[\mathbb{E}_{-b_{ijl}|s_{ijl}}(\frac{1}{\omega_{ii}})\|X_l \odot Y_j\|^2 s_{ijl} + \mathbb{E}_{-b_{ijl}|s_{ijl}}(\tau_{il})\right]^{-1}$
      $\mu(s_{ijl}) = -\sigma^2(s_{ijl})\left\{\left[Y_i + \mathbb{E}_{-b_{ijl}|s_{ijl}}\left(\frac{1}{\omega_{ii}}M_{-(i,j)}^{-l}\right)\right]^T [X_l \odot Y_j]s_{ijl}\right\}$
      Set $q_{vb}(s_{ijl}) \sim \text{Ber}(\psi_{ijl})$ where:
      $\psi_{ijl} = \mathbb{E}_{-s_{ijl}}\text{logit}(\pi_{ijl}) + \frac{1}{2}log\mathbb{E}_{-s_{ijl}}\tau_{il} -$
      $\frac{1}{2}log\left[\mathbb{E}_{-s_{ijl}}(\frac{1}{\omega_{ii}})\|X_l \odot Y_j\|^2 + \mathbb{E}_{-s_{ijl}}(\tau_{il})\right] +$
      $\frac{1}{2}\left[\mathbb{E}_{-s_{ijl}}(\frac{1}{\omega_{ii}})\|X_l \odot Y_j\|^2 + \mathbb{E}_{-s_{ijl}}(\tau_{il})\right]^{-1} \times$
      $\left[(X_l \odot Y_j)^T(Y_i + \mathbb{E}_{-s_{ijl}}\left[\frac{1}{\omega_{ii}}\right]\mathbb{E}_{-s_{ijl}}M_{-(i,j)}^{-l})\right]^2$
    end for
  end for
  ζ: maximum value of expectation difference for parameters before and after updates.
  Note: ζ can also be defined as improvement in evidence lower bound (ELBO), calculated as:

$$ELBO[q_{\text{vb}}(\boldsymbol{\theta})] = -\frac{np+p(p-1)q}{2}\text{log}2\pi + pq\left[a_\tau \text{log} b_\tau - \text{log}\Gamma(a_\tau) + \text{log}\Gamma(a_\tau + \frac{p-1}{2})\right] +$$
$$p(p-1)q\left[\text{log}\Gamma(a_\pi + b_\pi) - \text{log}\Gamma(a_\pi b_\pi) - \text{log}\Gamma(a_\pi + b_\pi + 1)\right] -$$
$$\sum_{i=1}^{p}\left\{Y_i^T\left(\sum_{j\neq i}^{p}\sum_{l=1}^{q}\mathbb{E}\beta_{ijl}Z_l \odot Y_j\right) - \frac{n+2}{4}\text{log}\frac{a_{\omega_{ii}}}{b_{\omega_{ii}}} + \text{log}(2K_{(n+2)/2}\sqrt{a_{\omega_{ii}}b_{\omega_{ii}}})\right\} -$$
$$\left[a_\tau + \frac{p-1}{2}\right]\sum_{i=1}^{p}\sum_{l=1}^{q}\left\{\text{log}\left[b_\tau + \frac{1}{2}\sum_{j\neq i}^{p}\mathbb{E}b_{ijl}^2\right]\right\} +$$
$$\sum_{i=1}^{p}\sum_{j\neq i}^{p}\sum_{l=1}^{q}\left\{\text{log }\Gamma(\mathbb{E}s_{ijl} + a_\pi) + \text{log }\Gamma(b_\pi + 1 - \mathbb{E}s_{ijl}) +\right.$$
$$\left.\frac{1}{2}\text{log}\left[2\pi\sigma^2(s_{ijl})\right] + \frac{1}{2} - \psi_{ijl}\text{ log}(\psi_{ijl}) - (1-\psi_{ijl})\text{ log}(1-\psi_{ijl})\right\}$$

end while

Algorithm S1. GraphR algorithm, related to Figure 1.

	Estimation strategy	Multiple-group graphs	Continuously-varying graphs	Extension to spatial covariates	Scalability	Uncertainty quantification	Positive definite
GraphR	Bayesian	✓	✓	✓	✓	✓	X
FGL, GGL [2]	Frequentist	✓	X	X	✓	X	✓
LASICH [12]	Frequentist	✓	X	X	✓	X	✓
Bayesian Joint Spike-and-Slab Graphical Lasso [4]	Bayesian	✓	X	X	✓	✓	✓
Gembag[16]	Bayesian	✓	X	X	✓	✓	✓
Joint graphical horseshoe [5]	Bayesian	✓	X	X	✓	X	✓
GGMReg[17]	Frequentist	✓	✓	X	✓	X	X
Multiple GGMs [11]	Bayesian	✓	X	X	X	✓	✓
Bayesian graphical regression [9]	Bayesian	✓	✓	X	X	X	Not required
Bayesian edge regression [15]	Bayesian	✓	✓	X	X	✓	X
GGMx[10]	Bayesian	✓	✓	X	X	X	✓

Table S1: Comparative overview of GraphR and other heterogeneous graphical modeling methods, related to Figure 3.

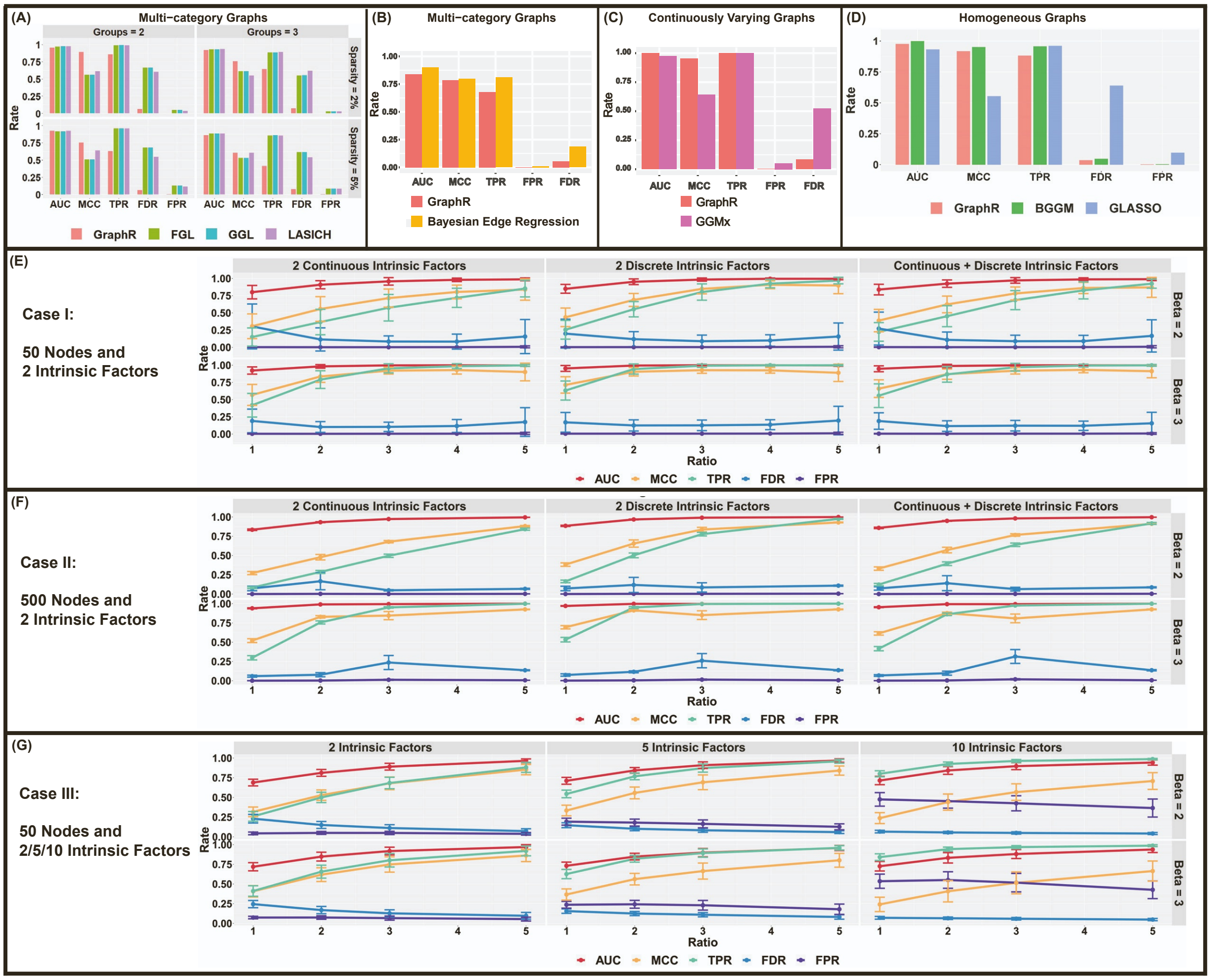

Figure S1: **Supplementary results on simulation study, related to Figure 3. (A)** shows selection performance in multi-category graphs setting with varying number of groups and sparsity. **(B)** summarizes the simulation results in multi-category graphs setting. GraphR is compared with Bayesian edge regression[15], a MCMC-based method in a low-dimensional scenario where $n = 100$, $p = 20$, $q = 2$ and sparsity level at 5%. **(C)** shows selection performance in homogenuous setting for 5% sparsity level. **(D)** summarizes the simulation results in continuously-varying graphs setting. GraphR is compared with GGMx[10], a MCMC-based method in a low-dimensional scenario where $n = 100$, $p = 20$, $q = 1$ and sparsity level at 5%. **E** illustrates the selection performance of edges in directed acyclic graphs with $p = 50, q = 2$, and varying intrinsic factor types and n/pq ratios. **E** illustrates the selection performance of edges in directed acyclic graphs with $p = 500, q = 2$, and varying intrinsic factor types and n/pq ratios. **F** illustrates the selection performance of edges in directed acyclic graphs with $p = 50$, and varying number of intrinsic factor and n/pq ratios.

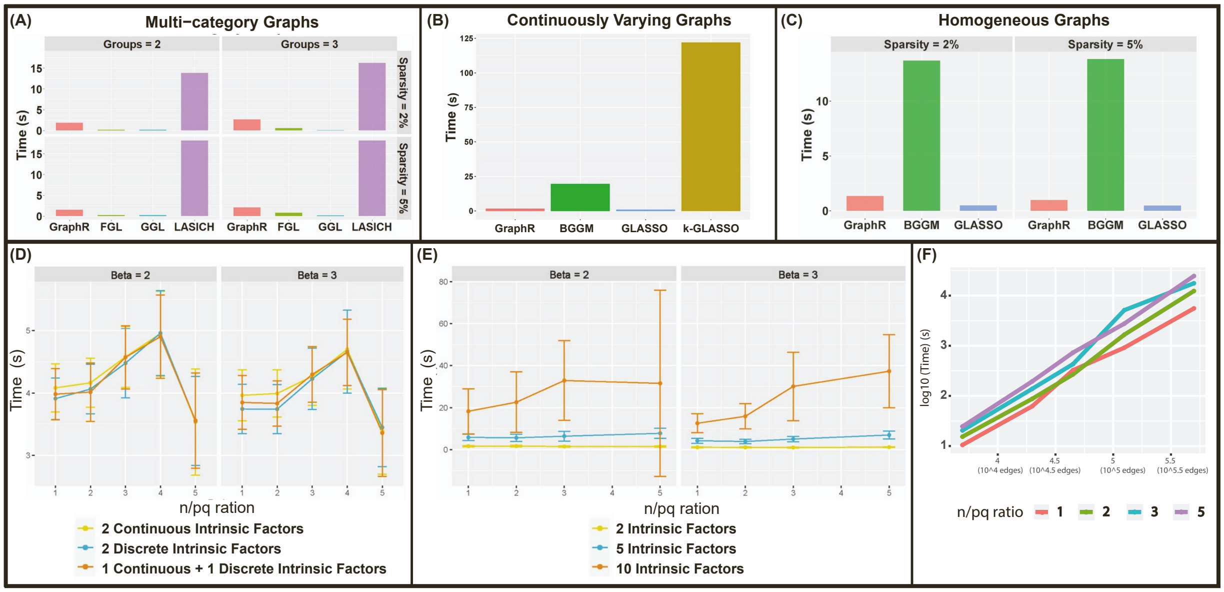

Figure S2: **Supplementary results on computation time under simulation settings, related to Figure 3.** **(A)** shows computation time in multi-category graphs setting with varying number of groups and sparsity. **(B)** shows computation time in continuously-varying graphs setting. **(C)** shows computation time in homogenuous setting for varying sparsity level. **(D)** illustrates computation time for directed acyclic graphs with $p = 50, q = 2$, and varying intrinsic factor types and n/pq ratios, and effect size. **(E)** illustrates computation time for directed acyclic graphs with $p = 50$, and varying number of intrinsic factor and n/pq ratios, and effect size. **(F)** shows computation times in the directed acyclic graph setting varying with the number of edges and n/pq ratios. The x-axis and y-axis represent log10 of the number of edges and computation time respectively, with different colors representing various n/pq ratios.

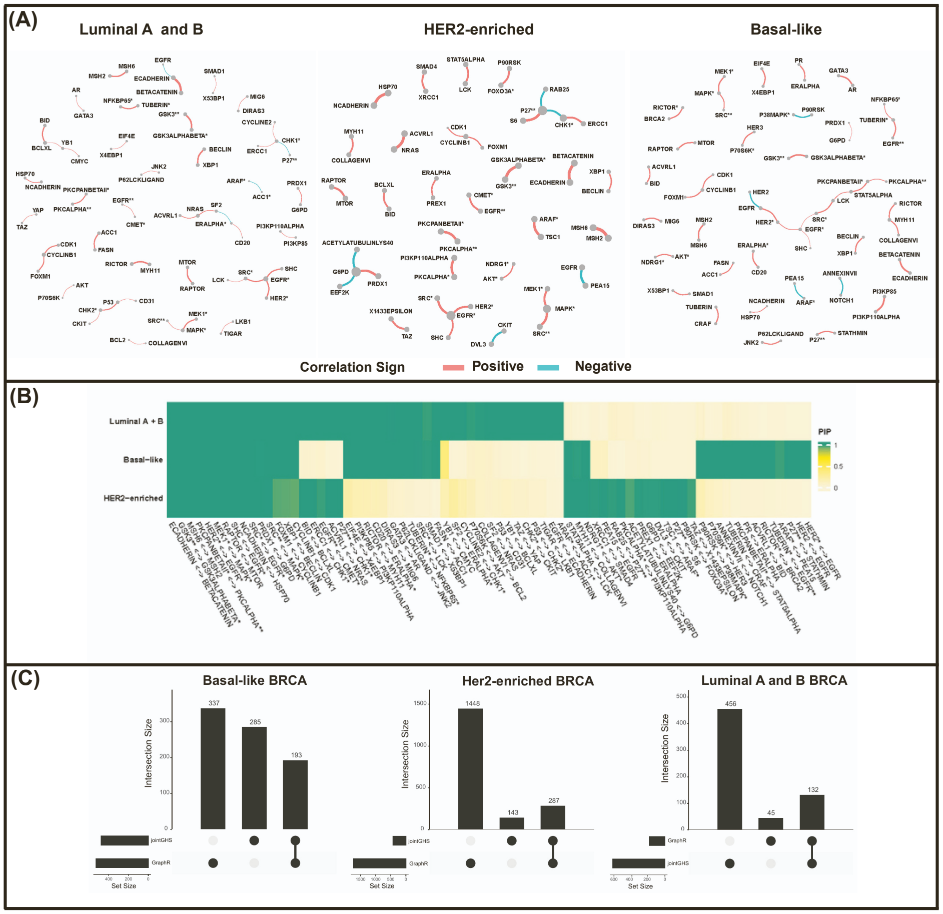

Figure S3: **Supplemental analysis of proteomic network-based characterization of intrinsic subtypes of breast cancer, related to Figure 4.** **(A)** shows networks of selected protein pairs in Luminal A+B, Her2-enriched and Basal-like BRCA respectively. The widths of edges are proportional to partial correlations. The sizes of nodes are proportional to connectivity degrees which are defined as the sum of magnitudes of the partial correlations. Sign of partial correlations are represented by color with red being positive and blue being negative. **(B)** shows heatmap of posterior inclusion probability (PIP) of selected edges in each PAM50 subtype of BRCA. We have compared with jointGHS which demonstrates the highest MCC other than GraphR in the multi-category graphs simulation setting. The upset plot in **(C)** summarizes the finding from intrinsic sybtypes of BRCA data between `GraphR` and jointGHS. For basal-like breast cancer, `GraphR` and jointGHS identify 193 edges in common. `GraphR` uniquely identifies 337 edges that are not selected by jointGHS, while jointGHS uniquely identifies 285 edges that are not selected by `GraphR`. For the Luminal A and B BRCA subtypes, both methods agree on 132 edges. `GraphR` uniquely identifies 45 edges, whereas jointGHS identifies 456 edges that are not found by `GraphR`. In the HER2-enriched BRCA subtype, `GraphR` and jointGHS agree on 287 edges. `GraphR` uniquely identifies 1448 edges, while jointGHS uniquely identifies 143 edges.

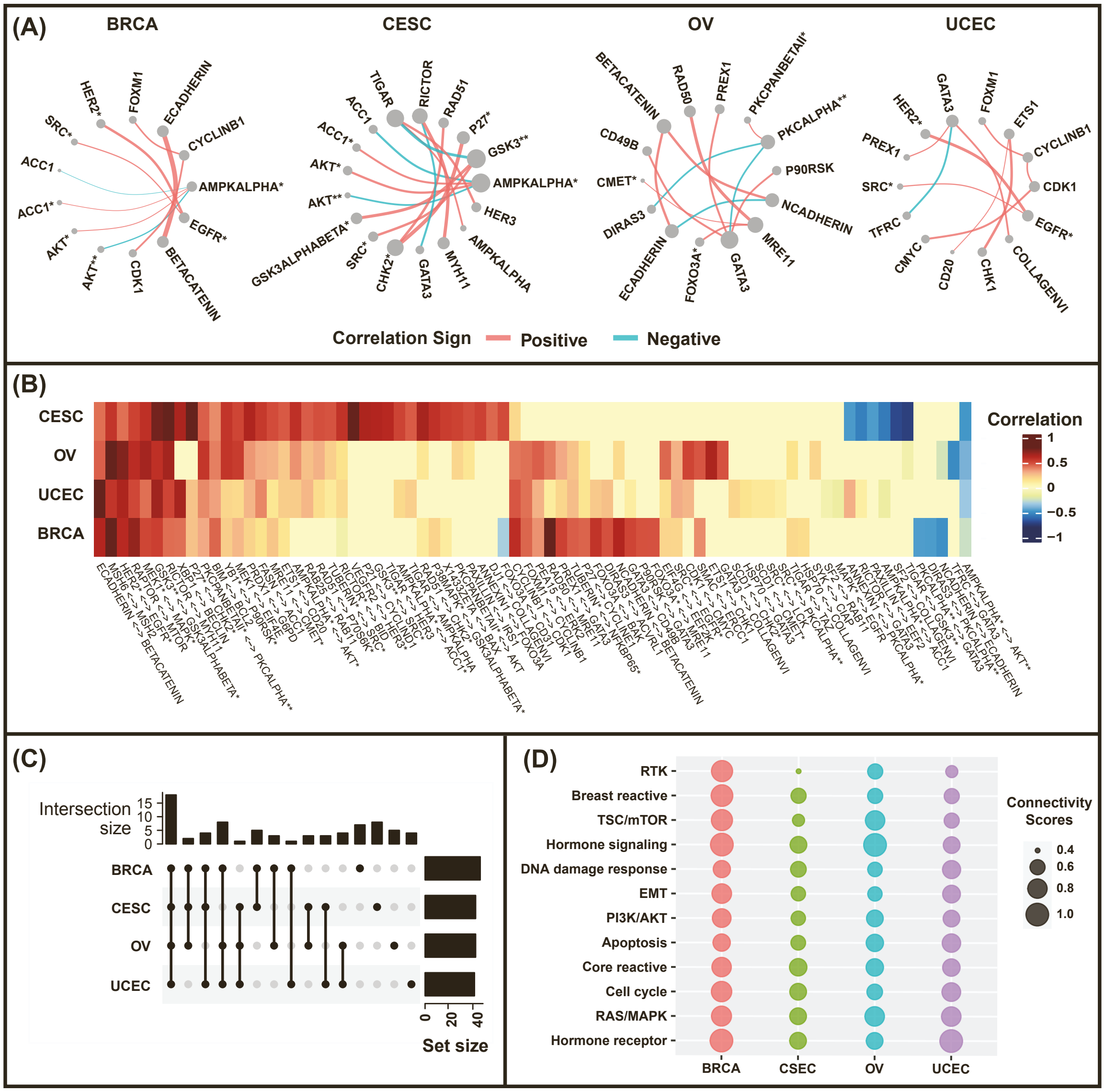

Figure S4: **Pan-Cancer Proteomic Network-based Characterization of Breast and Gynecologic Cancers, related to Figure 4.** The results are based on BRCA and three types of gynecologic cancers including cervical squamous cell carcinoma and endocervical adenocarcinoma (CESC), ovarian serous cystadenocarcinoma (OV) and uterine corpus endometrial carcinoma (UCEC). **(A)** Cancer-specific networks for proteins with top five connectivity degrees. **(B)** Heatmap of partial correlations between protein pairs for each cancer type. We only include significant protein pairs which are highly correlated with FDR based p-values < 0.01 and $|\text{partial correlation}| > 0.45$ for at least one type of cancer. **(C)** Upset plot for numbers of significant connections within or across cancer types based on the protein pairs included in (B). **(D)** Connectivity scores of pathways for each cancer type. Pathways are ordered based on the clustering of connectivity scores.

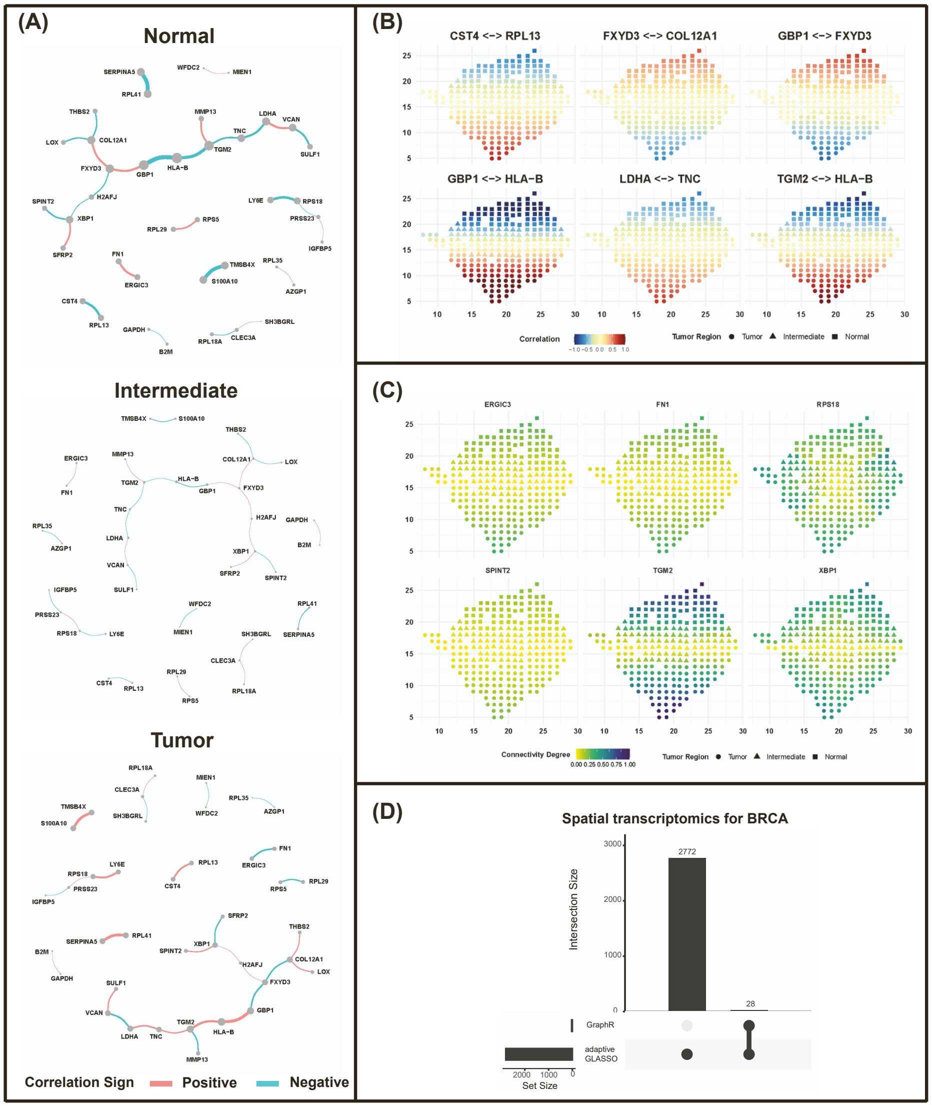

Figure S5: **Additional results for analysis of spatial transcriptomics data, related to Figure 6.** **(A)** shows the whole networks w.r.t. each spatial regions. The edges are proportional to the weighted average of partial correlations and nodes are proportional to the weighted connectivity degrees. We also display more spatial patterns of partial correlations**(B)** and connectivity degrees **(C)** for gene and gene pairs. The color bar indicates the values of correlations and connectivity degrees while shapes of point representing the spatial region. We implemented an adaptive GLASSO based approach for comparative study of findings from sptial transcriptomics data. For each spot, neighboring spots within a pre-specified radius of 1 are included, and we apply GLASSO within that neighborhood. `GraphR` identifies 28 edges, all of which are also detected by the adaptive GLASSO. However, the adaptive GLASSO identifies an addition 2,772 edges as significant, indicating a potential high rate of false positives. We summarize these results through the upset plot in **(D)**.

References

[1] Baladandayuthapani, V., Ji, Y., Talluri, R., Nieto-Barajas, L. E., and Morris, J. S. (2010). Bayesian random segmentation models to identify shared copy number aberrations for array cgh data. *Journal of the american statistical association*, 105(492):1358–1375.

[2] Danaher, P., Wang, P., and Witten, D. M. (2013). The Joint Graphical Lasso for Inverse Covariance Estimation Across Multiple Classes. *Journal of the Royal Statistical Society Series B: Statistical Methodology*, 76(2):373–397.

[3] Ji, Y., Yin, G., Tsui, K.-W., Kolonin, M. G., Sun, J., Arap, W., Pasqualini, R., and Do, K.-A. (2007). Bayesian mixture models for complex high dimensional count data in phage display experiments. *Journal of the Royal Statistical Society: Series C (Applied Statistics)*, 56(2):139–152.

[4] Li, Z., Mccormick, T., and Clark, S. (2019). Bayesian joint spike-and-slab graphical lasso. In *International Conference on Machine Learning*, pages 3877–3885. PMLR.

[5] Lingjærde, C., Fairfax, B. P., Richardson, S., and Ruffieux, H. (2022). Scalable multiple network inference with the joint graphical horseshoe. *arXiv preprint arXiv:2206.11820.*

[6] Liu, H., Roeder, K., and Wasserman, L. (2010). Stability approach to regularization selection (stars) for high dimensional graphical models. *Advances in neural information processing systems*, 23.

[7] Meinshausen, N. and Bühlmann, P. (2010). Stability selection. *Journal of the Royal Statistical Society: Series B (Statistical Methodology)*, 72(4):417–473.

[8] Morris, J. S., Brown, P. J., Herrick, R. C., Baggerly, K. A., and Coombes, K. R. (2008). Bayesian analysis of mass spectrometry proteomic data using wavelet-based functional mixed models. *Biometrics*, 64(2):479–489.

[9] Ni, Y., Stingo, F. C., and Baladandayuthapani, V. (2019). Bayesian Graphical Regression. *Journal of the American Statistical Association*, 114(525):184–197.

[10] Ni, Y., Stingo, F. C., and Baladandayuthapani, V. (2022). Bayesian Covariate-Dependent Gaussian Graphical Models with Varying Structure. *J. Mach. Learn. Res.*, 23(1).

[11] Peterson, C., Stingo, F. C., and Vannucci, M. (2015). Bayesian inference of multiple gaussian graphical models. *Journal of the American Statistical Association*, 110(509):159–174.

[12] Saegusa, T. and Shojaie, A. (2016). Joint estimation of precision matrices in heterogeneous populations. *Electronic Journal of Statistics*, 10(1):1341 – 1392.

[13] Storey, J. D. (2003). The positive false discovery rate: a bayesian interpretation and the q-value. *The Annals of Statistics*, 31(6):2013–2035.

[14] Titsias, M. and Lázaro-Gredilla, M. (2011). Spike and slab variational inference for multi-task and multiple kernel learning. *Advances in neural information processing systems*, 24.

[15] Wang, Z., Baladandayuthapani, V., Kaseb, A. O., Amin, H. M., Hassan, M. M., Wang, W., and Morris, J. S. (2022). Bayesian Edge Regression in Undirected Graphical Models to Characterize Interpatient Heterogeneity in Cancer. *Journal of the American Statistical Association*, 117(538):533–546.

[16] Yang, X., Gan, L., Narisetty, N. N., and Liang, F. (2021). Gembag: Group estimation of multiple bayesian graphical models. *Journal of machine learning research*, 22(54):1–48.

[17] Zhang, J. and Li, Y. (2022). High-Dimensional Gaussian Graphical Regression Models with Covariates. *Journal of the American Statistical Association*, 0(0):1–13.
